# Molecular solar thermal energy storage in photoswitch oligomers increases energy densities and storage times

**DOI:** 10.1038/s41467-018-04230-8

**Published:** 2018-05-16

**Authors:** Mads Mansø, Anne Ugleholdt Petersen, Zhihang Wang, Paul Erhart, Mogens Brøndsted Nielsen, Kasper Moth-Poulsen

**Affiliations:** 1 Department of Chemistry and Chemical Engineering, Chalmers University of Technology, Kemivägen 10, 412 96 Gothenburg, Sweden; 2 Department of Chemistry, University of Copenhagen, Universitetsparken 5, 2100 Copenhagen Ø, Denmark; 3 Department of Physics, Chalmers University of Technology, Kemivägen 10, 412 96 Gothenburg, Sweden

## Abstract

Molecular photoswitches can be used for solar thermal energy storage by photoisomerization into high-energy, meta-stable isomers; we present a molecular design strategy leading to photoswitches with high energy densities and long storage times. High measured energy densities of up to 559 kJ kg^−1^ (155 Wh kg^−1^), long storage lifetimes up to 48.5 days, and high quantum yields of conversion of up to 94% per subunit are demonstrated in norbornadiene/quadricyclane (**NBD/QC**) photo-/thermoswitch couples incorporated into dimeric and trimeric structures. By changing the linker unit between the **NBD** units, we can at the same time fine-tune light-harvesting and energy densities of the dimers and trimers so that they exceed those of their monomeric analogs. These new oligomers thereby meet several of the criteria to be met for an optimum molecule to ultimately enter actual devices being able to undergo closed cycles of solar light-harvesting, energy storage, and heat release.

## Abstract (web summary)

Molecular solar thermal systems are promising for storing solar energy but achieving high energy storage densities and absorption characteristics matching the solar spectrum is challenging. Here the authors present a design strategy for electronically coupled photoswitches which allow for high energy density storage for solar energy storage applications.

## Introduction

**Fig. 1:**
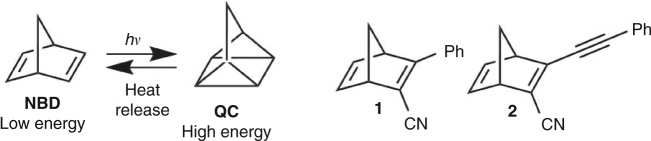
Molecular system. The norbornadiene (**NBD**)–quadricyclane (**QC**) couple and the previously reported monomeric norbornadienes **1** and **2**

Solar energy is a viable and inexhaustible source of energy for both electricity and heat production. In this context energy storage is a major challenge due to strong daily and seasonal variations in the availability of sunlight^1^. Molecular solar thermal (MOST) systems represent a promising avenue for harvesting and storing solar energy. In this approach a molecule is converted by photoisomerization into a higher-energy isomer, which is capable of storing the energy until released by a heat trigger or catalyst, converting the meta-stable isomer to the original light-harvesting isomer^2–10^. Norbornadienes have shown to be a promising candidate for MOST due to the high energy difference between the norbornadiene (**NBD**) and quadricyclane (**QC**) photoisomer of approximately 96 kJ mol^−1^ (Fig. 1), and the system has been shown to undergo heat-release by the action of cobalt-based catalysts^11–13^. For these systems it has been previously shown that donor–acceptor substitutions provide an effective means for red-shifting the longest-wavelength absorption, improving the solar spectrum match; compounds **1** and **2** present examples of such derivatives^14^.

Two crucial challenges for a useful MOST system are the achievement of a sufficiently high energy storage density, ideally higher than 300 kJ kg^−1^ and light-harvesting in the visible region^15^. Functionalization of the norbornadiene with donor and acceptor units has been used to tune absorption maxima, but this positive effect on solar absorption is counter-balanced by higher molecular weights, and hence lower energy densities^11,16^. Moreover, the positive effect on solar absorption typically also has a detrimental effect on the energy storage time, which is lowered when absorption is redshifted^14^. One possible solution to overcome this anti-correlation between redshifting and energy density that we present here is to couple one chromophore unit to several photoswitches. Here, in the context of **NBD**/**QC** systems, we find that it is attractive to form dimers or trimers, where the **NBD** units share the same donor and/or acceptor. Previously dimers of photoswitches such as dihydroazulenes^17^, dithienylethenes^18,19^, azobenzenes^20,21^, and spiropyranes^22,23^ have been reported. Even other norbornadiene dimers, linked via phthalatamide bonds or conjugated ketones, have been prepared and studied, but showed very low quantum yields^24,25^.

**Fig. 2:**
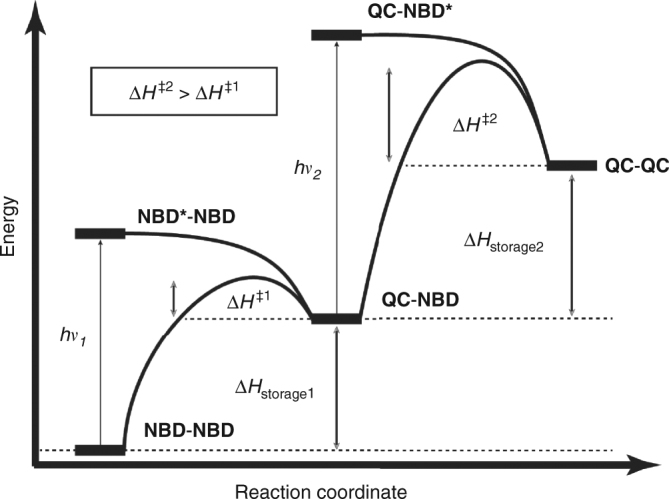
Energy diagram. Schematic representation of the difference in half-life and irradiation energy for the two switching events

The idea for the present work was to engineer the stability of the high energy photoisomer by having two electronically coupled photoswitches with separate barriers for thermal conversion (Fig. 2). The reasoning for this is that there is a blue shift after the first isomerisation (**NBD–NBD** to **QC−NBD**), leading to a higher energy of isomerisation of the second switching event (**QC−NBD** to **QC−QC**). Simultaneously having a shared donor reduces the molecular weight per norbornadiene unit. We chose to synthesize two series of dimers as well as one trimer of **NBD** that resemble the previously synthesized donor−acceptor monomers **1** and **2**, with absorption onsets of 358 and 374 nm, respectively^14^.

## Results

### Synthesis

**Fig. 3:**
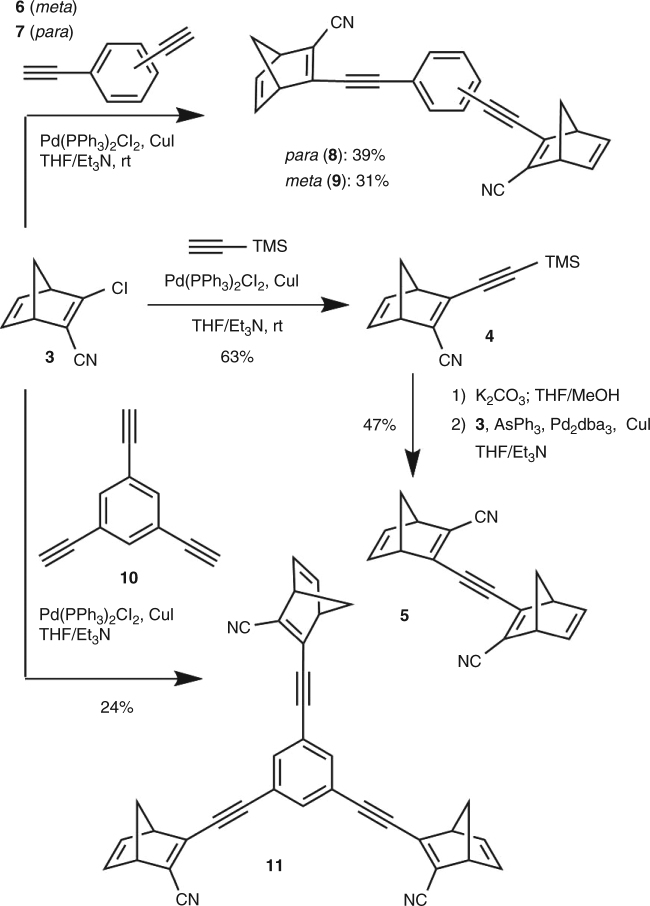
Synthesis of alkyne-linked oligomers. Synthesis of acetylene-linked dimers **5**, **8**, and **9** as well as the trimer **11**

The synthesis of NBD dimers and trimers is shown in Fig. 3 (all isolated as mixtures of diastereoisomers; only one of these is shown in each case). It was possible to obtain three alkyne-linked dimers as well as one trimer by Sonogashira couplings using the previously reported 2-chloro-3-cyanonorbornadiene **3** as substrate^14^. The simplest dimer was synthesized by Sonogashira coupling with trimethylsilylacetylene, affording **4** in a yield of 63%. The silyl protecting group was removed with potassium carbonate in THF/MeOH, and the resulting terminal alkyne was subjected to a second Sonogashira coupling with **3** to afford the acetylene-linked NBD dimer **5** in 47% yield. In the second Sonogashira reaction, standard conditions using phosphine ligands did not give any of the wanted products due to instability of the terminal acetylene. Instead, the Pd_2_dba_3_/AsPh_3_ catalyst system^26^ was used as it improved the reaction rate. Utilizing the same reaction conditions as for the synthesis of **4** with either 1,3- or 1,4-diethynylbenzene (**6** or **7**) gave the two NBD dimers **8** and **9** in 39 and 31% yield, respectively. Similarly, with 1,3,5-triethynylbenzene **10** as substrate, a triple Sonogashira coupling furnished **11** in a yield of 24%.

**Fig. 4:**
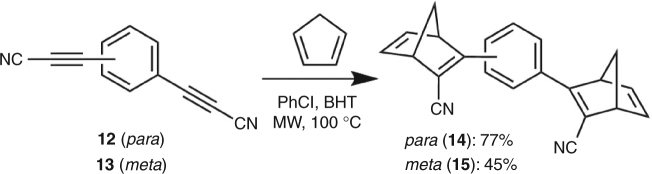
Synthesis of phenylene-linked dimers. Synthesis of phenylene-linked norbornadiene dimers **14** and **15**. BHT “butylated hydroxytoluene”, i.e., 2,6-di(tert-butyl)-4-methylphenol; MW microwave heating

To further investigate the effect of the spacer group, a second series with two **NBD** units directly linked to a phenylene spacer was synthesized (Fig. 4). Initial attempts employed **3** as substrate. Yet, a Suzuki cross-coupling with a double boronic acid did not yield any product (see Supplementary Figs. 6 and 8 for conditions). Instead, it was found that the desired product could easily be obtained from a Diels-Alder reaction between the known^27^ *para* and *meta* bis(cyanoethynyl)benzenes **12** and **13**, respectively, with cyclopentadiene (under microwave heating) affording the dimers **14** and **15** in yields of 77 and 45%.

### Spectroscopy

**Fig. 5:**
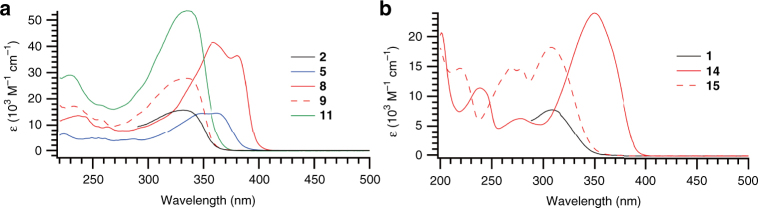
Optical absorption spectra. **a** UV−Vis absorption spectra of acetylene-linked norbornadienes. Black line: **2**, blue line: **5**, red line: **8**, red dashed line**: 9**, green line: **11**. **b** UV−Vis absorption spectra of phenylene-linked norbornadienes. Black line: **1**, red line: **14**, red dashed line: **15**. The spectra of monomers **1** and **2** were recorded in toluene

The new norbornadiene compounds were investigated with UV-Vis absorption spectroscopy, and the spectra are shown in Fig. 5. All of the spectroscopic studies were performed in cyclohexane as this solvent gave a larger spectral window for analysis of the switching events than toluene. As expected there is a large difference in the absorption onset and maxima between the *meta* and *para* isomers. The systems that are *para* substituted (**5**, **8**, and **14**) give the highest onset of absorbance, which can be explained by the linear conjugation pathway between the two **NBD** chromophores, providing a larger conjugated system compared to the meta substituted compounds. The spectra of the *meta* substituted dimer **9** and the trimer **11** have very similar absorption onsets; yet, the trimer with three **NBD** units has a higher molar absorptivity. Interestingly the spectra are also similar to that of the corresponding monomer **2** (Fig. 5) in regard to longest-wavelength absorbance maxima and onset. This trend is also evident in the molar absorptivity, which for compound **2** is approximately half of the value for **9**. The same trend is seen for compounds **1** and **15**, which have comparable absorption onsets and the longest-wavelength absorption maxima. The molar absorptivity of **15** is more than twice that of **1**.

**Fig. 6:**
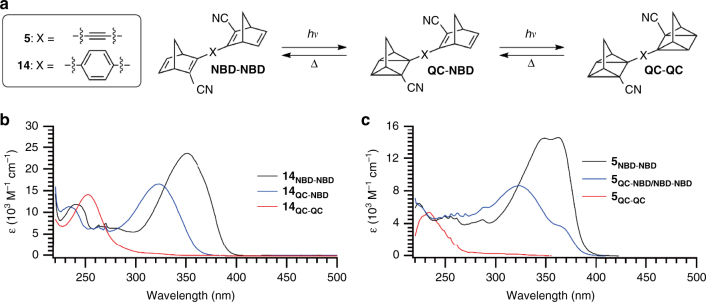
Stepwise conversion of dimers. **a** Stepwise isomerization of **5** and **14** to the corresponding **QC−NBD** and **QC−QC** isomers. **b** UV−Vis spectra of **14** and its isomers. Black line: **14**_**NBD−NBD**_, blue line: **14**_**QC−NBD**_, red line: **14**_**QC−QC**_. **c** UV−Vis spectra of **5**, **5**_**QC−QC**_ and the thermal-photostationary state between **5** and **5**_**QC−NBD**_. Black line: **5**_**NBD−NBD**_, blue line: the thermal-photostationary state between **5** and **5**_**QC−NBD**_, red line: **5**_**QC−QC**_

The next objective was to study the switching properties of the new **NBD−NBD** derivatives. Upon irradiation close to the absorbance maxima (365 or 340 nm), the dimers and trimer could all be fully converted to the corresponding **QC−QC** dimers, data shown in Table 1. It was attempted to isomerize all of the norbornadiene dimers selectively to the intermediate **QC−NBD** form by irradiating around the onset of absorption. Switching to the **QC−NBD** could be achieved for compounds **5** and **14** by irradiation at 405 nm. In addition the switching events were also studied by NMR spectroscopic analysis. For compound **5** it was found that irradiation at 405 nm forms a thermal-photostationary state between **5**_**NBD−NBD**_ and **5**_**QC−NBD**_, which is dependent on the intensity of the lamp and the temperature of the sample, due to fast back conversion of **5**_**QC−NBD**_ (vide infra). The ratio found from NMR studies was 54:46 (see Supplementary Fig. 118). For **14**, the isomerization to **QC−NBD** form could be obtained almost quantitatively by irradiation at 405 nm, and the spectra of **14**_**NBD−NBD**_, **14**_**QC−NBD**_ and **14**_**QC−QC**_ are shown in Fig. 6. From NMR spectroscopic studies it was found that upon irradiation at 405 nm, small amounts of **14**_**QC−QC**_ were formed together with **14**_**QC−NBD**_, and it was ultimately possible to convert the system to **14**_**QC−QC**_ although this took over 40 h (see Supplementary Fig. 121 for details). Looking at the absorbance spectrum it is evident that even though it is possible to photoisomerize **14**_**QC−NBD**_ at 405 nm, the absorbance at 405 nm is close to non-existent, and thereby giving a spectral window for the sequential switching.

**Table 1:** Physical data.

Compound	*ε*_max_ (*λ*_max_) (NBD−NBD)	*ε*_*max*_ *(λ*_max_) (QC−QC)	*A*_Onset_ (nm) (NBD−NBD)	*t*_½ (QC−QC 25 °C)_	*t*_½(QC−NBD 25 °C)_	*Φ* (per photo-conversion event)	*Φ*_NBD−NBD→QC−NBD_ (*Φ*_QC−NBD→QC−QC_)
**5**	14,766 (362 nm)	5379 (234 nm)	404	2.88 days	7.08 min	–	47%^a^
**8**	41,005 (359 nm)	34,014 (300 nm)	411	4.33 h	–	94%^b^ 85%^a^	–
**9**	28,131 (334 nm)	24,191 (258 nm)	381	14.2 h	–	83%^b^	–
**11**	53,355 (336 nm)	43,821 (264 nm)	376	13.2 h	–	71%^b^	–
**14**	23,578 (350 nm)	14,175 (252 nm)	400	10.6 days	2.08 days	–	73%^a^ (51%)
**15**	18,316 (308 nm)	16,612 (224 nm)	362	48.5 days	–	53%^c^ 52%^b^	–

Absorption profile, kinetic parameters for **QC**-to-**NBD** conversions, and photoisomerization (**NBD**-to-**QC**) quantum yields in cyclohexane

Quantum yield measured at

^a^365 nm; ^b^340 nm; ^c^310 nm

### Quantum yields

Quantum yields of the photoisomerization events in cyclohexane were also measured for all the molecules using a reference procedure (Table 1)^28^. For some of the molecules, when the absorbance profile allowed it, the quantum yield was measured at different wavelengths. This was done in order to independently probe the quantum yields of the two consecutive photoisomerization processes, ring closure of **NBD−NBD** and subsequently of **QC−NBD** (where their absorption profiles were taken into consideration). For compounds **8**, **9**, **11**, and **15** the quantum yields are calculated based on the number of photoconversion events, taking into account that two or three **NBD** subunits are present per oligomer (see Table 1). The assumption is made that the units are behaving like independent **NBD** units and are having a similar absorption profile. For **14** and **5**, which exhibit sequential switching, it was possible to measure the quantum yield for the **NBD−NBD** to **QC−NBD** process selectively. For **5**_**NBD−NBD**_ it was only possible to measure the quantum yield of the first photoconversion into intermediate **5**_**QC−NBD**_ due to the fast rate of backreaction. In the case of **14** it is mainly the **14**_**NBD−NBD**_ form that absorbs, confirmed by the presence of an isosbestic point in the measurement, allowing the formation of **14**_**QC−QC**_ to be neglected (see Supplementary Fig. 129). Furthermore, **14** could be irradiated at 405 nm to form **14**_**QC−NBD**_, and subsequently the quantum yield at 340 nm could be measured (see Table 1).

### Kinetics

The kinetics of the **QC−QC** to **NBD−NBD** backreactions were determined at three different temperatures and the half-life at 25 °C, *t*_½_ (25  °C), was determined from an Arrhenius analysis (Table 1).

Interestingly, two markedly different rates of back conversion could be measured for both **5** and **14** (see Table 1). The half-life for the conversion of **QC−NBD** to **NBD−NBD** is remarkably faster than that for the backreaction of the fully isomerized **QC–QC** isomers. It was possible to fit the data for conversion of **5**_**QC−QC**_ and 14_**QC−QC**_ all the way back to the corresponding **NBD−NBD** isomers by single-exponential fits, due to the significantly faster conversion of **QC−NBD** to **NBD−NBD**. Fascinatingly, the first conversion from **QC−QC** to **QC−NBD** exhibits a higher barrier compared to the **QC−NBD** to **NBD−NBD** conversion, which we ascribe to the coupled nature of the two different electronic systems. For compounds **8**, **9**, **11**, and **15** a discrimination of the different barriers could not be carried out due to similarities in the electronic properties of the **QC−NBD** and **NBD−NBD** forms of the molecules.

### Cyclability

**Fig. 7:**
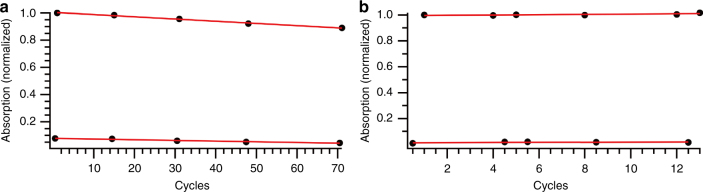
Cyclability test. **a** Cyclability for **8** showing the normalized absorbance at 362 nm in cyclohexane at 50 °C irradiated at 365 nm. **b** Cyclability for **14** showing the normalized absorbance at 350 nm in cyclohexane at 70 °C irradiated at 340 nm

To investigate the stability over several cycles of photoisomerization followed by thermal back-reaction, a cycling test of **8** and **14** was performed (Fig. 7) by monitoring the UV−Vis absorption of the compounds at 362 and 350 nm respectively. The cyclability of **8** was tested over 71 cycles at 50 °C giving a decomposition of 0.16% per cycle, and for **14** the decomposition per cycle at 70 °C was found to be 0.11%, showing that the decomposition for both these molecules is very small. This decomposition is shown as a decrease in the absorbance for **8**, while for **14** there is an increase in the absorbance. This increase can be caused by evaporation of the solvent, or a decomposition product with a higher absorptivity. As some evaporation of the solvent could not be avoided, the cyclability of **14** is likely better than observed. Since the backreaction is relatively slow it was not possible to do the experiment at a lower temperature.

### Energy densities

To obtain the energy densities of the high-energy **QC** isomers, the norbornadiene derivatives were irradiated at either 365 or 340 nm in CDCl_3_ (CDCl_3_ was selected due to the low solubility in cyclohexane), until fully converted; the solutions were concentrated by a stream of nitrogen and the resulting **QC** subjected to differential scanning calorimetry (DSC) (Table 2). Compounds **11** and **14** partly decomposed at the temperatures of the DSC experiment (>140 °C), so the energy densities of these compounds are not included. Isomerization of **5** in either CDCl_3_ or toluene-*d8* for DSC experiments (~1 M) could not be achieved by irradiation at either 365 or 340 nm, possibly due to lower quantum yields in these solvents as well as the short lifetime of the **QC−NBD** form (minutes) making quantitative conversions impractical. The heat release data for **QC−QC** dimers of **8**, **9**, and **15** are listed in Table 2. For all of the molecules the heat release was also calculated using density functional theory (DFT) calculations (coordinates listed in Supplementary Data)^19,20^. The DSC sample for **9** was a mixture of **9**_**QC−QC**_ and **9**_**QC−NBD**_, which results in a large uncertainty, even though a correction taking this into account was performed. This is probably the reason for the large difference between calculated and measured values.

**Table 2:** Energy densities.

Compound	Δ*H* (kJ mol^−1^)	Δ*H* (kJ kg^−1^)	Δ*H*_calc_ (kJ mol^−1^)	Δ*H*_calc_ (kJ kg^−1^)
**1**	122	629	114.3	591.5
**2**	–	–	118.5	545.9
**5**	–	–	237.6	927.0
**8**	183.3	514.2	231.1	648.5
**9**	98.7	276.8	239.5	671.9
**11**	–	–	362.4	731.3
**14**	–	–	237.9	771.4
**15**	172.5	559.3	232.0	752.3

Measured and calculated heat release of the **QC−QC** isomers

## Discussion

In conclusion, we have developed efficient synthetic protocols for the preparation of high-performance norbornadiene dimers and trimers. The quantum yield of photoconversion is near quantitative, up to 94% per NBD subunit. For the *para* phenylene-bridged **NBD** dimer **14**, conversions between isomers occurred stepwise—both in the forward and backwards direction—and with significantly different **NBD** absorption maxima for the **NBD−NBD** and **NBD−QC** states. Our molecular design demonstrates the attractive feature of blueshifting the **NBD** absorption after the first **NBD**-to-**QC** photoisomerization as it ultimately furnishes a very energetic **QC−QC** state after the second photoisomerization as well as a prolonged storage time. As the first energy discharge (**QC−QC** to **QC−NBD** backreaction) is the rate-determining step, full discharge of the system will conveniently be promoted by triggering this event. The system contrasts in this respect related dihydroazulene-vinylheptafulvene dimers incorporated in macrocyclic structures for which instead the first discharge is fast and the second slow^29^.

The calculated energy densities of the dimer and trimer systems of up to 927 kJ kg^−1^ (257 Wh kg^−1^) and measured densities up to 559 kJ kg^−1^ (155 Wh kg^−1^) greatly exceed the original targets of 300 kJ kg^-1^^15^ highlighting the potential of applying molecular photoswitches in future solar thermal energy storage technologies. As the strongly coupled dimers **5** and **14** show a higher barrier for the first step in the thermal conversion compared to the second thermal conversion, this molecular motif can be used in future design of high-performance molecular switches for solar thermal energy storage as well as other applications. In future work a donor group could be attached to the benzene ring to give an enhanced redshifting of the absorbance spectra, or changing the benzene ring to more electron-rich aromatics. Another interesting approach would be to have two **NBD** units directly linked, in an attempt to increase the multimode switching by a greater coupling between the **NBD** subunits.

## Methods

### Materials

Cyclopentadiene was distilled by cracking dicyclopentadiene over iron filings and stored at −80 °C, prior to use. Tetrahydrofuran used for Sonogashira coupling reactions was distilled over a sodium/benzophenone couple. All other chemicals were used as purchased from commercial sources. Purification of products was carried out by flash chromatography on silica gel (40–63 μm, 60 Å). Thin-layer chromatography was carried out using aluminum sheets precoated with silica gel.

### Measurements

Infrared (IR) spectra recorded on a Perkin-Elmer Frontier FT-IR instrument as films evaporated from CDCl_3_ onto an ATR attachment, where relative intensities are denoted as vw (very weak), w (weak), m (medium), s (strong), and sh (shoulder). All melting points and heat release of neat quadricyclanes were recorded on a Mettler Toledo DSC 2 apparatus. ^1^H NMR (400 MHz) and ^13^C NMR (100 MHz) spectra were recorded on a Varian 400 MHz instrument or a Bruker 500 MHz instrument with a noninverse cryoprobe using the residual solvent as the internal standard (CDCl_3_, ^1^H 7.26 ppm and ^13^C 77.16 ppm or *d12*-cyclohexane, ^1^H 1.38 ppm and ^13^C 26.43 ppm). All chemical shifts are quoted on the δ scale (ppm), and all coupling constants (J) are expressed in Hz. All solution-based spectroscopic measurements were performed in cyclohexane in a 1-cm path length cuvette on either a Cary 60 or a Cary 100 UV−Vis spectrophotometer, scanning the wavelength from 600 to 200 nm coupled with Peltier temperature control. Photoswitching for wavelengths 310, 340, 365 and 405 nm were performed using Thorlabs LED lamps M310L3, M340L4, M365F1 and M405LP1 respectively. The thermal back reaction was performed by heating the sample (cuvette) by a Peltier unit in the UV−Vis spectrophotometer. Quantum yields were measured by the published procedure^28^ using a high concentration regime (absorption above 2 at the irradiation wavelength) using potassium ferrioxalate and tris-phenanthroline iron (II) complex as a chemical actinometer and Thorlabs LED lamps M310L3, M340L4 and a fiber-coupled LED (M365F1) respectively for irradiation. Irradiation was done perpendicular to the cuvette in a fixed setup approximately 5 cm distance between LED and sample, with stirring, ensuring no movement of the lamp during the experiment. Mass spectra (HRMS) were either acquired by electron spray ionization (ESI) using an Agilent 1260 Infinity fitted with an Agilent 6120 quadrupole for or an ESP-MALDI-FT-ICR spectrometer equipped with a 7 T magnet (calibration of the instrument was done with NaTFA cluster ions). Elemental analyses were performed at London Metropolitan University. Compounds **3**^14^, **12**^27^, and **13**^27^ were made by their respective literature methods.

### Computational methods

Calculations for the heat release from QC to NBD were performed at the B3LYP/6-311+G* level  using the NWChem package^30,31^. All norbornadiene and quadricyclane compounds were fully relaxed. The storage energy was computed as the energy difference between the norbornadiene and quadricyclane forms.

### Data availability

Data supporting this study can be found in the Supplementary Information file. Upon request to the corresponding author, further information supporting this study can be made available.

## Electronic supplementary material

Supplementary Information
