# Supplementary material for: Molecular solar thermal energy storage in photoswitch oligomers increases energy densities and storage times
Source: Nat Commun. 2018 May 16;9:1945. doi: 10.1038/s41467-018-04230-8 (PMC5956078; doi:10.1038/s41467-018-04230-8)
Supplement: Supplementary file 1 — Supplementary Information [file 41467_2018_4230_MOESM1_ESM.pdf]

Molecular Solar Thermal Energy Storage: high energy densities and long storage times by photoswitch oligomers

MADS MANSØ, ANNE UGLEHOLDT PETERSEN, ZHIHANG WANG, PAUL ERHART, MOGENS BRØNDSTED NIELSEN, KASPER MOTH-POULSEN.

Contents

Experimental **page S2**

NMR Spectra **page S8**

UV-Vis Absorption and Switching Studies **page S31**

Heat release (DSC) **page S47**

NMR studies of photo conversion **page S53**

Photoisomerisation quantum yields **page S59**

Cyclability test **page S71**

Coordinates from DFT calculations **page S72**

Experimental

Compound 4

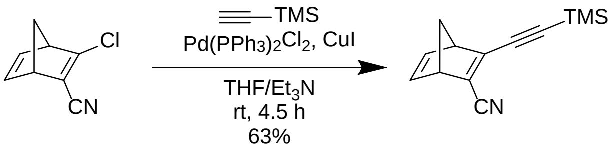

Supplementary Figure 1. Synthesis. Synthesis of compound **4**.

To an argon flushed solution of 2-chloro-3-cyano norbornadiene (604 mg, 3.98 mmol) in anhydrous THF (40 mL) was added $Pd(PPh_3)_2Cl_2$ (143 mg, 204 μmol) and CuI (75 mg, 394 μmol). Et_3N (15 mL) was added slowly followed by trimethylsilylacetylene (1.7 mL, 12 mmol) and the reaction was stirred at rt for 4 h. The reaction mixture was poured into H_2O (100 mL), extracted with CH_2Cl_2 (3 x 100 mL). The combined organic phases were dried over Na_2SO_4, filtered and concentrated *in vacuo*. Flash column chromatography (5% EtOAc/Heptane) gave **4** as a slightly orange oil (535 mg, 63%). ^{1}H NMR (500 MHz, $CDCl_3$): δ = 6.87–6.79 (m, 2H), 3.88–3.82 (m, 1H), 3.78–3.72 (m, 1H), 2.26 (dt, J = 7.0, 1.6 Hz, 1H), 2.18 (dt, J = 7.0, 1.6 Hz, 1H), 0.24 (s, 9H) ppm. ^{13}C NMR (126 MHz, $CDCl_3$): δ = 154.09, 142.01, 141.48, 129.78, 116.12, 115.04, 97.63, 73.10, 57.32, 54.19, -0.21 ppm. HR-MS (ESI+ FT-ICR): m/z = 236.08583 [$M+Na^+$], calcd. for [$C_{13}H_{15}NNaSi^+$]: m/z = 236.08660.

Compound 5

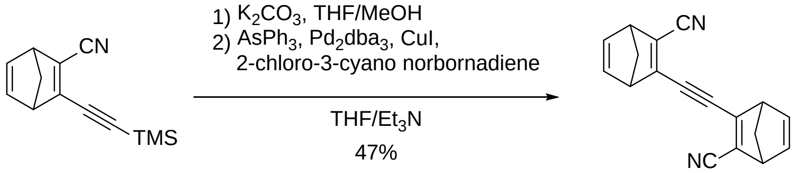

Supplementary Figure 2. Synthesis. Synthesis of compound **5**.

To a solution of **4** (203 mg, 951 μmol) in THF/MeOH (20 mL, 1:1) was added K_2CO_3 (500 mg, 3.62 mmol). After 20 min of stirring at rt the mixture was pulled through a plug of SiO_2 (40-63 μm, CH_2Cl_2). The mixture was divided into two. Half of the mixture was concentrated to approximately 10 ml *in vacuo*, before 25 mL of Et_3N (25 mL) was added and the mixture was concentrated to approximately 10 mL. Freshly distilled THF (10 mL) and 2-chloro-3-cyanonorbornadiene (110 mg, 726 μmol) were added and the mixture was flushed with argon. $AsPh_3$ (57 mg, 186 μmol), Pd_2dba_3 (21 mg, μmol) and CuI (9 mg, 47 μmol) were added and the mixture was stirred at rt for 17 h. The mixture was poured into brine/H_2O (100 mL, 1:1) and extracted with CH_2Cl_2 (3 x 30 mL). The combined organic phases were dried over Na_2SO_4, filtered and concentrated *in vacuo*. The crude mixture was purified by flash column chromatography (15% EtOAc/Heptane) furnishing **5** as a yellow solid (57 mg, 47%). IR = 3074w, 2997m, 2987sh, 2949m, 2919sh, 2874m, 2850w, 2205s, 1597m, 1560w, 1538w cm^{-1}. M.p.: 152–155 °C. 15% EtOAc/Heptane: R_f = 0.26. ^{1}H NMR (500 MHz, $CDCl_3$): δ = 6.88 – 6.84 (m, 2H), 3.93 – 3.90 (m, 1H), 3.88–3.85 (m, 1H), 2.34–2.30 (m, 1H), 2.25–2.22 (m, 1H) ppm. (All multiplets. Due to diastereoisomers). ^{13}C NMR (126 MHz, $CDCl_3$): δ = 152.53, 142.08,

142.06, 141.62, 141.61, 131.43, 131.42, 115.86, 99.67, 99.66, 73.17, 57.36, 57.35, 54.65 ppm. MS (ESI^+): *m/z* = 279 $[M+Na]^+$. EA ($C_{18}H_{12}N_2$): calcd. C 84.35, H 4.72, N 10.93; found C 84.30, H 4.81, N 10.86.

Compound 8

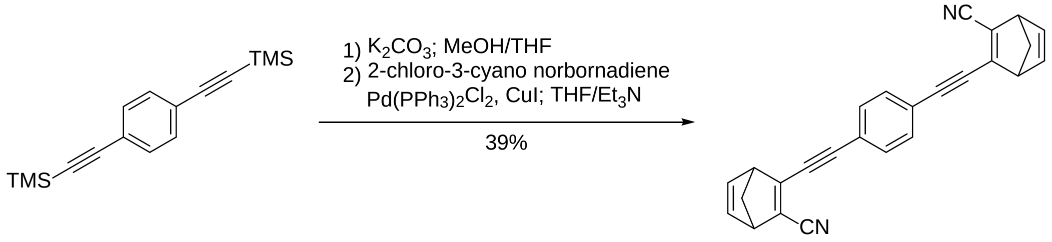

Supplementary Figure 3. Synthesis. Synthesis of compound **8**.

To a solution of 1,4-bis((trimethylsilyl)ethynyl)benzene (237 mg, 876 µmol) in MeOH/THF (20 mL, 1:1) was added K_2CO_3 (495 mg, 3.58 mmol) and the mixture was stirred for 30 min before it was pulled through a plug of silica (43-60 µm, CH_2Cl_2) and concentrated *in vacuo* to approximately 2 mL. Et_3N (25 mL) was added and once again the mixture was concentrated *in vacuo* to approximately 10 mL. Freshly distilled THF (10 mL) and 2-chloro-3-cyanonorbornadiene (436 mg, 2.88 mmol) was added and the mixture was flushed with argon. CuI (25 mg, 131 µmol) and $Pd(PPh_3)_2Cl_2$ (99 mg, 141 µmol) was added and the reaction mixture was stirred at rt for 7h. The mixture was pulled through a plug of silica (40-63 µm, CH_2Cl_2) and concentrated *in vacuo*. Flash column chromatography (toluene) gave **8** as a slightly impure yellow solid (225 mg, ~72%). Recrystallization (CH_2Cl_2/heptane) gave pure **8** as a mixture of diastereoisomers (122 mg, 39%). IR = 3110w, 3076w, 3038w, 3004sh, 2985m, 2948m, 2873m, 2207s, 2190s, 1602w, 1578s, 1571sh, 1557m cm^{-1}. M.p. = Decomposes slowly above 140 °C. ^1H NMR (400 MHz, $CDCl_3$): δ = 7.49 (s, 4H), 6.89 (ddd, J = 5.1, 3.0, 0.8 Hz, 2H), 6.86 (ddd, J = 5.1, 3.0, 0.9 Hz, 2H), 3.91 (ddtd, J = 3.0, 2.5, 1.6, 0.9 Hz, 2H), 3.86 (ddtd, J = 3.0, 2.5, 1.6, 0.8 Hz, 2H), 2.33 (dt, J = 7.1, 1.6 Hz, 2H), 2.24 (dt, J = 7.1, 1.6 Hz, 2H). ^{13}C NMR (101 MHz, $CDCl_3$): δ = 153.73, 142.26, 141.41, 132.00, 129.46, 123.11, 116.31, 106.99, 85.48, 73.01, 57.26, 54.29. HR-MS (ESI+ FT-ICR): *m/z* = 357.13899 [M+H+], calcd. for $[C_{26}H_{17}N_2^+]$: *m/z* = 357.13862. EA ($C_{36}H_{21}N_3$): calcd. C 87.62, H 4.52, N 7.86; found C 87.79, H 4.60, N 7.82.

Compound 9

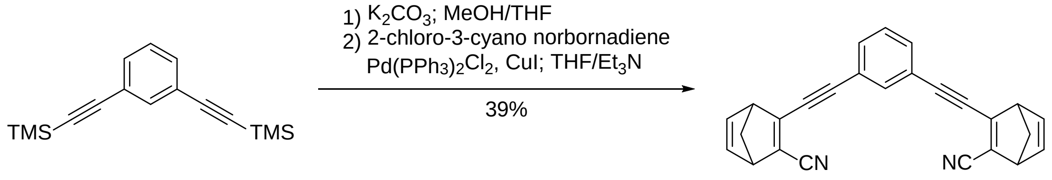

Supplementary Figure 4. Synthesis. Synthesis of compound **9**.

To a solution of 1,3-bis((trimethylsilyl)ethynyl)benzene (433 mg, 1.60 mmol) in MeOH/THF (30 mL: 1:1) was added K_2CO_3 (516 mg, 3.73 mmol) and the mixture was stirred for 30 min before it was pulled through a plug of silica (43-60 µm, CH_2Cl_2) and concentrated *in vacuo* to approximately 2 mL. Et_3N (25 mL) was added and once again the mixture was concentrated *in vacuo* to approximately 15 mL. Freshly distilled THF (15 mL) and 2-chloro-3-cyanonorbornadiene (910 mg, 6.00 mmol) was added and the mixture was flushed with argon. CuI (43 mg, 226 µmol) and $Pd(PPh_3)_2Cl_2$ (225 mg, 141 µmol) was added and the reaction mixture was stirred at rt for 17h. The mixture was pulled through a plug of silica (40-63 µm, CH_2Cl_2) and concentrated *in vacuo*. Flash column chromatography (toluene) gave **9** as a mixture of diastereoisomers, a light brown semicrystalline oil (163 mg, 29%). IR = 3072w, 2997m, 2985sh, 2947m, 2873m, 2207s, 2190s,

1598s, 1586sh, 1571w, 1557m cm^{-1}. ^{1}H NMR (400 MHz, $CDCl_3$): δ = 7.68–7.62 (m, 1H), 7.53–7.45 (m, 2H), 7.37–7.33 (m, 1H), 6.91–6.82 (m, 4H), 3.92–3.88 (m, 2H), 3.86–3.83 (m, 2H), 2.32 (dt, J = 7.1, 1.7 Hz, 2H), 2.23 (dt, J = 7.1, 1.6 Hz, 2H). ^{13}C NMR (100 MHz, $CDCl_3$): δ = 153.83, 142.25, 141.44, 135.07, 132.73, 129.40, 128.95, 122.74, 116.32, 106.34, 83.98, 73.04, 57.27, 54.28. HR-MS (ESI+): m/z = 357.1384 [M+H$^+$], calcd. For [$C_{26}H_{17}N_2^+$]: m/z = 357.1386.

Compound 11

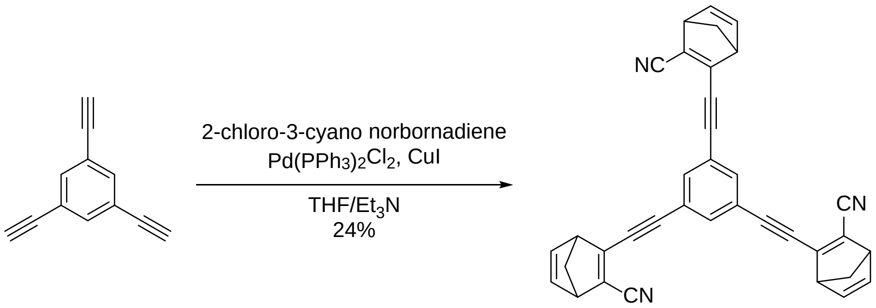

Supplementary Figure 5. Synthesis. Synthesis of compound **11**.

To an argon flushed solution of 2-chloro-3-cyanonorbornadiene (633 mg, 4.18 mmol) in THF/Et_3N (25 mL, 3:2) was added $Pd(PPh_3)_2Cl_2$ (150 mg, 214 μmol) and CuI (41 mg, 215 μmol). After 5 min of stirring at rt, the 1,3,5-ethynylbenzene (99 mg, 659 μmol) was added and the mixture was stirred for 24 h. The mixture was filtered through a plug of silica (40-63 μm, CH_2Cl_2), before being subjected to flash column chromatography (20% EtOAc/Heptane to 35% EtOAc/Heptane, loaded in CS_2) followed by flash column chromatography (50% CH_2Cl_2/Heptane to 100% CH_2Cl_2) yielding **11** as a mixture of diastereoisomers, a slightly yellow solid (80 mg, 24%). IR = 3972w, 2996m, 2984sh, 2947m, 2873m, 2208s, 2193sh, 1593s, 1555m cm^{-1}. M.p.: 169–172 °C. CH_2Cl_2: R_f = 0.56. 35%EtOAc/heptanes: R_f = 0.47. ^{1}H NMR (500 MHz, $CDCl_3$): δ = 7.63 (s, 3H), 6.91 – 6.87 (m, 6H), 3.93 – 3.91 (m, 3H), 3.88 – 3.86 (m, 3H), 2.34 (dt, J = 7.1, 1.7 Hz, 3H), 2.25 (dt, J = 7.1, 1.7 Hz, 3H) ppm. ^{13}C NMR (126 MHz, $CDCl_3$): δ = 153.31, 142.07, 141.35, 135.18, 130.15, 123.28, 116.01, 104.83, 84.56, 73.01, 57.08, 54.23 ppm. HR-MS (ESI+ FT-ICR): m/z = 496.18209 [M+H+], calcd. for [$C_{36}H_{22}N_3^+$]: m/z = 496.18082. EA ($C_{36}H_{21}N_3$): calcd. C 87.25 , H 4.27, N 8.48; found C 87.29, H 4.19, N 8.56.

Compound 14

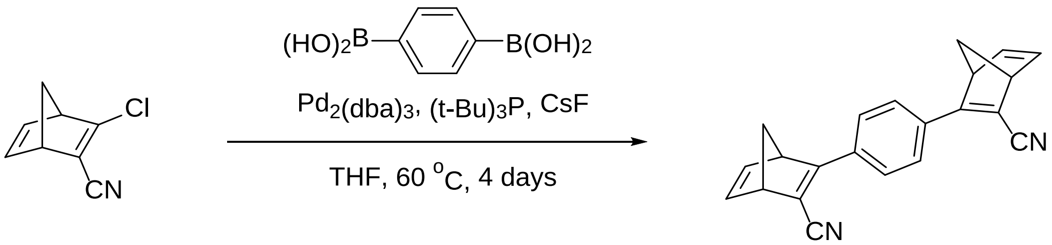

Supplementary Figure 6. Synthesis. Synthesis of compound **14**.

Method 1: To a nitrogen flushed mixture of 2-chloro-3-cyanonorbornadiene (354 g, 2.34 mmol) in THF (25 mL) was added 1,4-phenylenediboronic acid (186 mg, mmol), cesium fluoride (547 mg, mmol), Bis(dibenzylideneacetone)palladium(0) (104 mg, 0.114 mmol) and tri-tert-butylphosphine (0.34, 1 M, 0.34

mmol) under nitrogen, and the mixture was stirred at 60 °C for 4 days. After which the mixture was cooled to ambient temperature, and quenched with saturated aqueous NH_4Cl (40 mL), diluted with water (20 mL) and the mixture was extracted with CH_2Cl_2 (3 x 50 mL). The combined organic phases were dried over Mg_2SO_4, follow by removal of the solvent under reduced pressure. The residue was subjected to flash column chromatography (toluene) which did not give any product.

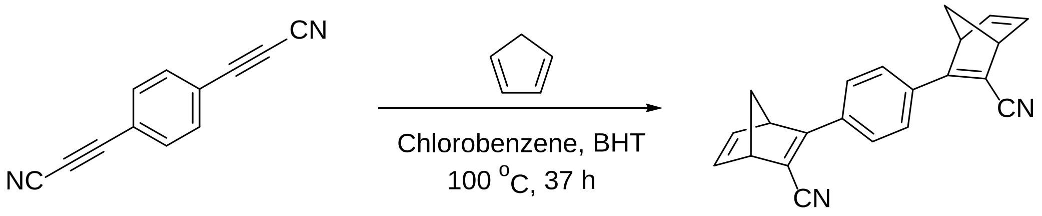

Supplementary Figure 7. Synthesis. Synthesis of compound **14**.

Method 2: A sealed tube containing 3,3'-(1,4-phenylene)dipropiolonitrile (152 mg, 0.863 mmol) and cyclopentadiene (0.5 mL, 5.95 mmol) and BHT (~5 mg) dissolved in chlorobenzene (3 mL) was heated in a microwave for 37 h at 100 °C. The resulting mixture was directly subjected to flash column chromatography (CH_2Cl_2) followed by recrystallization from CH_2Cl_2/heptane to give the product as a diasteomeric mixture which is a slightly yellow solid (206 mg, 77%).

3,3'-(1,4-phenylene)bis(bicyclo[2.2.1]hepta-2,5-diene-2-carbonitrile) 14: R_f=0.51 (CH_2Cl_2). IR = 3123w, 3072w, 2991m, 2946m, 2872m, 2251w, 2195s, 1585m, 1558m cm^{-1}. M.p. = Decomposes slowly above 160 °C. ^{1}H NMR (400 MHz, $CDCl_3$): δ = 7.78 (br s, 4H), 6.95 – 9.93 (m, 2H), 6.88 – 6.85 (m, 2H), 4.13 (ddtd, J = 3.2, 2.5, 1.6, 0.9 Hz, 2H), 3.96 (ddtd, J = 3.1, 2.5, 1.6, 0.9 Hz, 2H), 2.29 (dt, J = 6.9, 1.6 Hz, 2H), 2.22 (dt, J = 6.9, 1.6 Hz, 2H) ppm. ^{13}C NMR (100 MHz, $CDCl_3$): δ = 169.72, 169.71, 143.20, 143.20, 140.41, 134.43, 134.43, 127.00, 118.62, 118.27, 71.43, 71.40, 55.27, 54.10 ppm. MS (ESI$^+$): m/z = 331 $[M+Na]^+$. Analysis calcd (%) for $C_{22}H_{16}N_2$ (308.38): C 85.69, H 5.23, N 9.08; found: C 85.55, H 5.07, N 9.18.

Compound 15

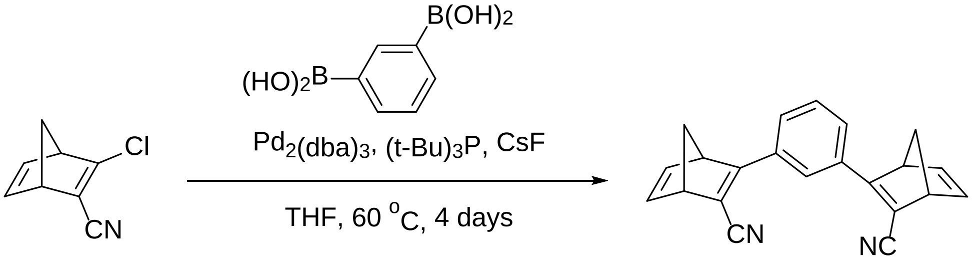

Supplementary Figure 8. Synthesis. Attempted synthesis of compound **15** by Suzuki coupling.

Method 1: To a nitrogen flushed mixture of 2-chloro-3-cyanonorbornadiene (346 g, 2.28 mmol) in THF (25 mL) was added 1,3-phenylenediboronic acid (185 mg, 1.12 mmol), cesium fluoride (544 mg, 3.58 mmol), Bis(dibenzylideneacetone)palladium(0) (102 mg, 0.114 mmol) and tri-tert-butylphosphine (0.34, 1 M, 0.34 mmol) under nitrogen, and the mixture was stirred at 60 °C for 4 days. After which the mixture was cooled to ambient temperature, and quenched with saturated aqueous NH_4Cl (40 mL), diluted with water (20 mL) and extracted with CH_2Cl_2 (3 x 50 mL). The combined organic phases were dried over Mg_2SO_4, follow by

removal of the solvent under reduced pressure. The residue was subjected to flash column chromatography (toluene) which did not give any product.

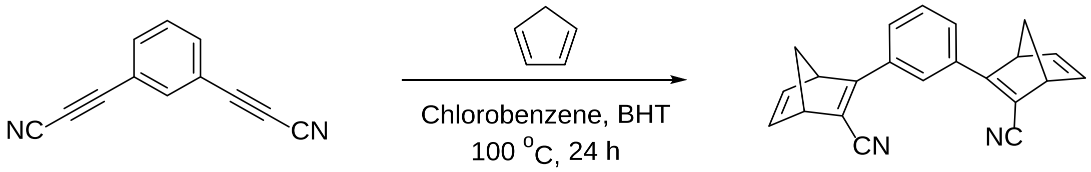

Supplementary Figure 9. Synthesis. Synthesis of compound **15**.

Method 2: A sealed tube containing 3,3'-(1,3-phenylene)dipropiolonitrile (303 mg, 1.72 mmol), cyclopentadiene (1 mL, 11.9 mmol), chlorobenzene (1 mL) and BHT (~5mg) was heated for 24 hours at 100 °C. The resulting mixture was purified by flash column chromatography (eluent CH_2Cl_2:hexane 1:1) followed by crystallization (CH_2Cl_2/heptane) to give the product as a diasteriomeric mixture which are white needles (237 mg, 45 %).

3,3'-(1,3-phenylene)bis(bicyclo[2.2.1]hepta-2,5-diene-2-carbonitrile) 15: R_f = 0.68 (CH_2Cl_2). IR = 3070w, 2993m, 2946m, 2872m, 2251w, 2197s, 1578sh, 1568m, 1558m cm^{-1}. M.p. = 169-170 °C. ^1H NMR (500 MHz, $CDCl_3$): δ = 8.08 (td, *J* = 1.8, 0.4 Hz, 1H), 8.05 (td, *J* = 1.8, 0.4 Hz, 1H), 7.77 (dd, *J* = 7.8, 1.8 Hz, 2H), 7.76 (dd, *J* = 7.8, 1.8 Hz, 2H), 7.50 (td, *J* = 7.8, 0.4 Hz, 1H), 7.50 (td, *J* = 7.8, 0.4 Hz, 1H), 7.14 – 6.73 (m, 8H), 4.19 – 4.16 (m, 4H), 3.97 – 3.95 (m, 4H), 2.32 – 2.28 (m, 4H), 2.22 (br dt, *J* = 6.9, 1.5 Hz, 4H) ppm. ^{13}C NMR (125 MHz, $CDCl_3$): δ = 170.35, 170.27, 143.09, 143.08, 140.57, 140.54, 133.75, 133.74, 129.67, 127.81, 127.80, 124.30, 118.38, 118.35, 118.31, 118.29, 71.56, 71.45, 55.15, 55.14, 54.25, 54.21 ppm. MS (ESI^+): *m/z* = 331 $[M+Na]^+$. Analysis calcd (%) for $C_{22}H_{16}N_2$ (308.38): C 85.69, H 5.23, N 9.08; found: C 85.53, H 5.41, N 9.07.

General procedure for generation of QC isomers

The corresponding **NBD** dimer or trimer in $CDCl_3$ in an NMR tube was irradiated[3] at either 340 or 365 nm overnight, before the solvent was removed by a stream of nitrogen.

$\mathbf{8_{QC\text{-}QC}}$

^1H NMR (400 MHz, $CDCl_3$): δ = 7.33 (s, 4H), 2.58 (dd, *J* = 5.0, 2.6 Hz, 2H), 2.45 (dt, *J* = 12.1, 1.5 Hz, 2H), 2.38 (dd, *J* = 5.0, 2.6 Hz, 2H), 2.33 (dq, *J* = 5.0, 1.5 Hz, 2H), 2.21 (dt, *J* = 12.1, 1.5 Hz, 2H), 2.10 (dq, *J* = 5.0, 1.5 Hz, 2H). ^{13}C NMR (100 MHz, $CDCl_3$): δ = 131.60, 122.72, 118.15, 85.98, 85.49, 33.50, 32.56, 32.38, 25.65, 25.33, 18.42, 14.77.

$\mathbf{9_{QC\text{-}QC}}$

^1H NMR (400 MHz, $CDCl_3$): δ = 7.49–7.47 (m, 1H), 7.35–7.31 (m, 2H), 7.24–7.19 (m, 1H), 2.58 (dd, *J* = 5.0, 2.6 Hz, 2H), 2.46 (dt, *J* = 12.1, 1.5 Hz, 2H), 2.38 (dd, *J* = 5.0, 2.6 Hz, 2H), 2.33 (dq, *J* = 5.0, 1.5 Hz, 2H), 2.21 (dt, *J* = 12.1, 1.5 Hz, 2H), 2.10 (dq, *J* = 5.0, 1.5 Hz, 2H). Not fully isomerized in $CDCl_3$. Distinguishing the carbon peaks were not possible.

$\mathbf{11_{QC\text{-}QC\text{-}QC}}$

^1H NMR (400 MHz): δ = 7.37 (s, 3H), 2.58 (dd, *J* = 5.0, 2.5 Hz, 6H), 2.45 (dt, *J* = 12.1, 1.4 Hz, 6H), 2.39 (dd, *J* = 4.9, 2.5 Hz, 6H), 2.32 (dq, *J* = 5.0, 1.4 Hz, 6H), 2.21 (dt, *J* = 12.1, 1.5 Hz, 6H), 2.11 (dq, *J* = 4.9, 1.4 Hz, 6H). ^{13}C

NMR (100 MHz, $CDCl_3$): δ = 133.97, 123.60, 118.07, 85.35, 84.26, 33.47, 32.58, 32.39, 25.65, 25.35, 18.27, 14.67.

$14_{QC\text{-}QC}$

^{1}H NMR (400 MHz, $CDCl_3$): δ = 7.17 (s, 4H), 2.64 (dd, *J* = 4.9, 2.6 Hz, 2H), 2.46 – 2.41 (m, 2H), 2.38 (dq, *J* = 4.9, 1.4 Hz, 2H), 2.26 – 2.23 (m, 4H), 1.90 – 1.87 (m, 2H) ppm. ^{13}C NMR (100 MHz, $CDCl_3$): δ = 170.35, 170.27, 143.09, 143.08, 140.57, 140.54, 133.75, 133.74, 129.67, 127.81, 127.80, 124.30, 118.38, 118.35, 118.31, 118.29, 71.56, 71.45, 55.15, 55.14, 54.25, 54.21 ppm

$15_{QC\text{-}QC}$

IR = 3060w, 2932w, 2861w, 2217s, 1606m, 1580w. ^{1}H NMR (400 MHz, $CDCl_3$): δ = 7.30 – 7.23 (m, 1H), 7.10 (d, *J* = 1.8 Hz, 1H), 7.08 (dd, *J* = 1.9, 0.5 Hz, 1H), 6.99 (dtd, *J* = 8.1, 1.8, 0.5 Hz, 1H), 2.64 (dt, *J* = 5.2, 2.7 Hz, 1H), 2.46 (dt, *J* = 3.5, 1.4 Hz, 1H), 2.43 (dt, *J* = 3.5, 1.4 Hz, 1H), 2.38 (ddq, *J* = 4.9, 1.4, 0.9 Hz, 2H), 2.29 (dt, *J* = 4.8, 2.4 Hz, 1H), 2.24 (q, *J* = 1.4 Hz, 1H), 2.21 (q, *J* = 1.3 Hz, 1H), 1.96 – 1.92 (m, 1H) ppm. ^{13}C NMR (100 MHz, $CDCl_3$): δ = 170.35, 170.27, 143.09, 143.08, 140.57, 140.54, 133.75, 133.74, 129.67, 127.81, 127.80, 124.30, 118.38, 118.35, 118.31, 118.29, 71.56, 71.45, 55.15, 55.14, 54.25, 54.21 ppm.

NMR Spectra

Compound 4

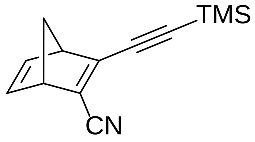

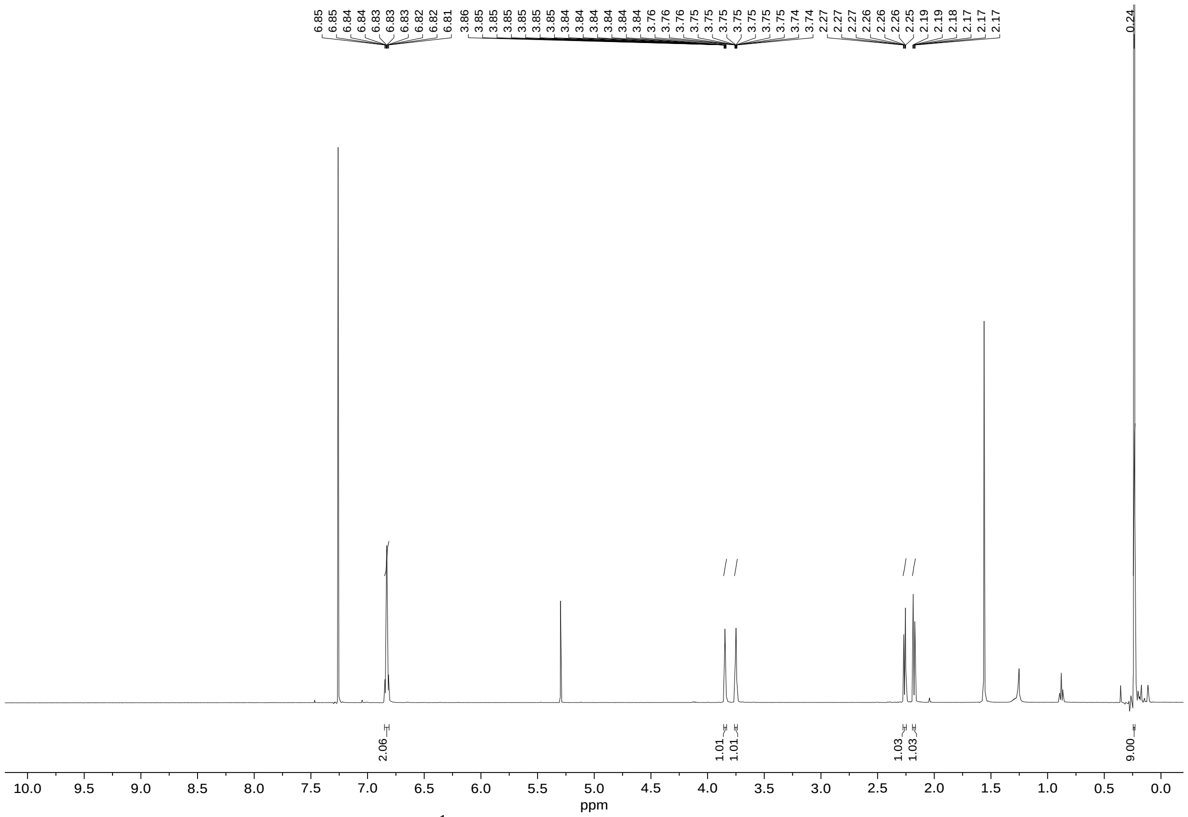

Supplementary Figure 10. NMR spectrum. ^{1}H NMR (500 MHz) of **4** in $CDCl_3$.

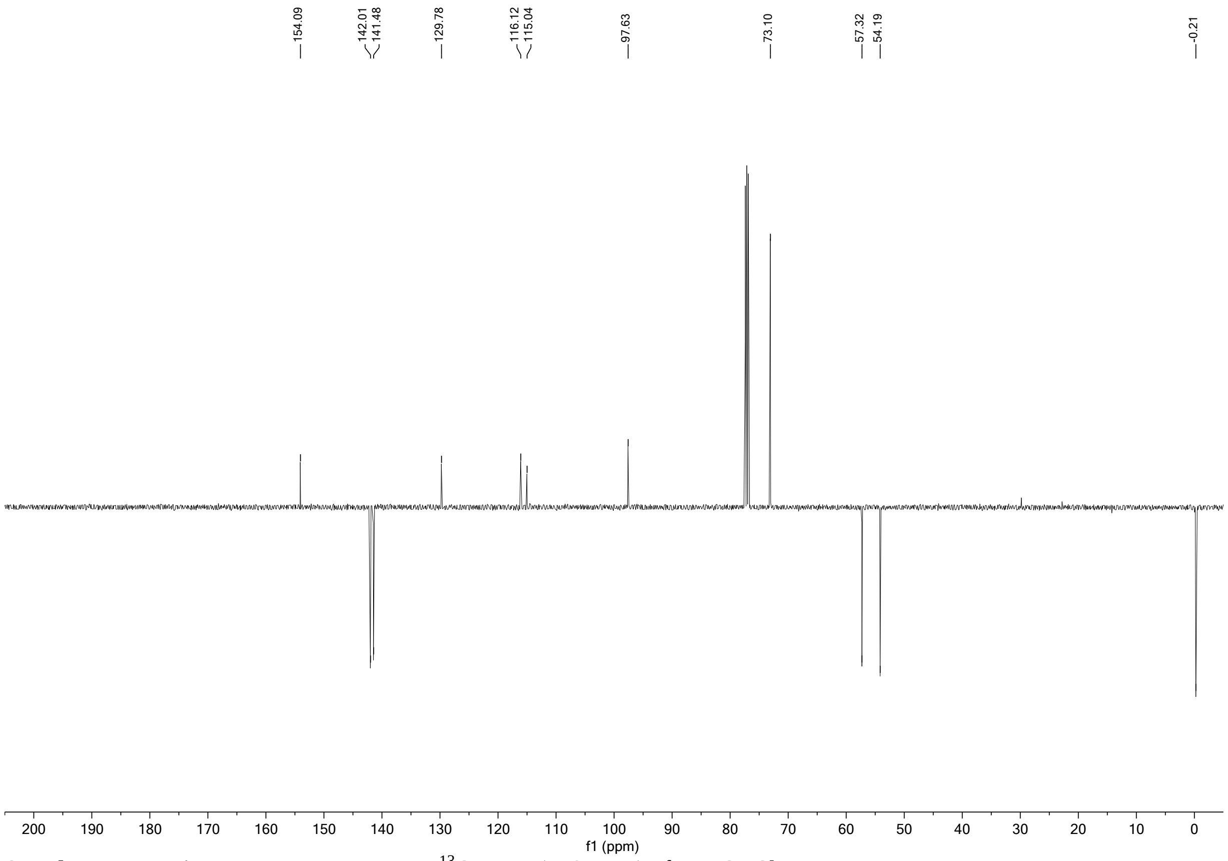

Supplementary Figure 11. NMR spectrum. ^{13}C NMR (126 MHz) of **4** in $CDCl_3$.

Compound 5

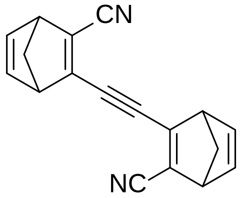

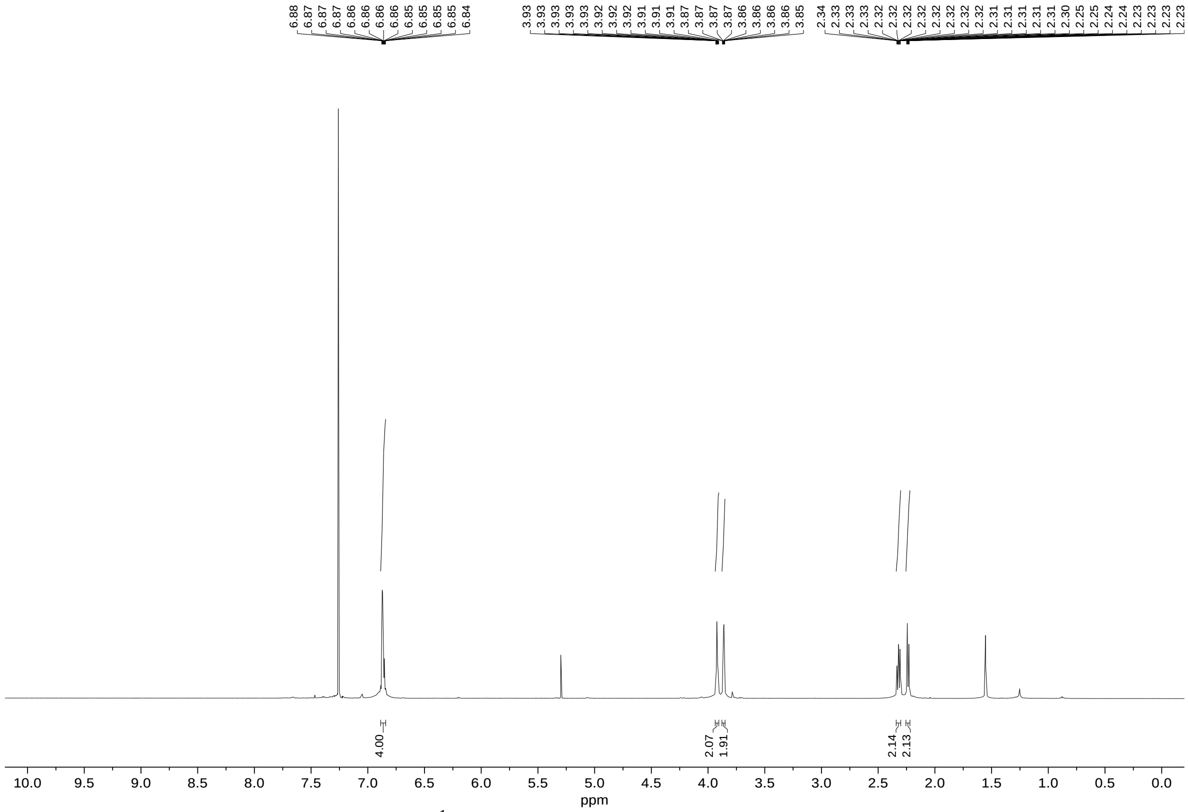

Supplementary Figure 12. NMR spectrum. ^{1}H NMR (500 MHz) of **5** in $CDCl_3$.

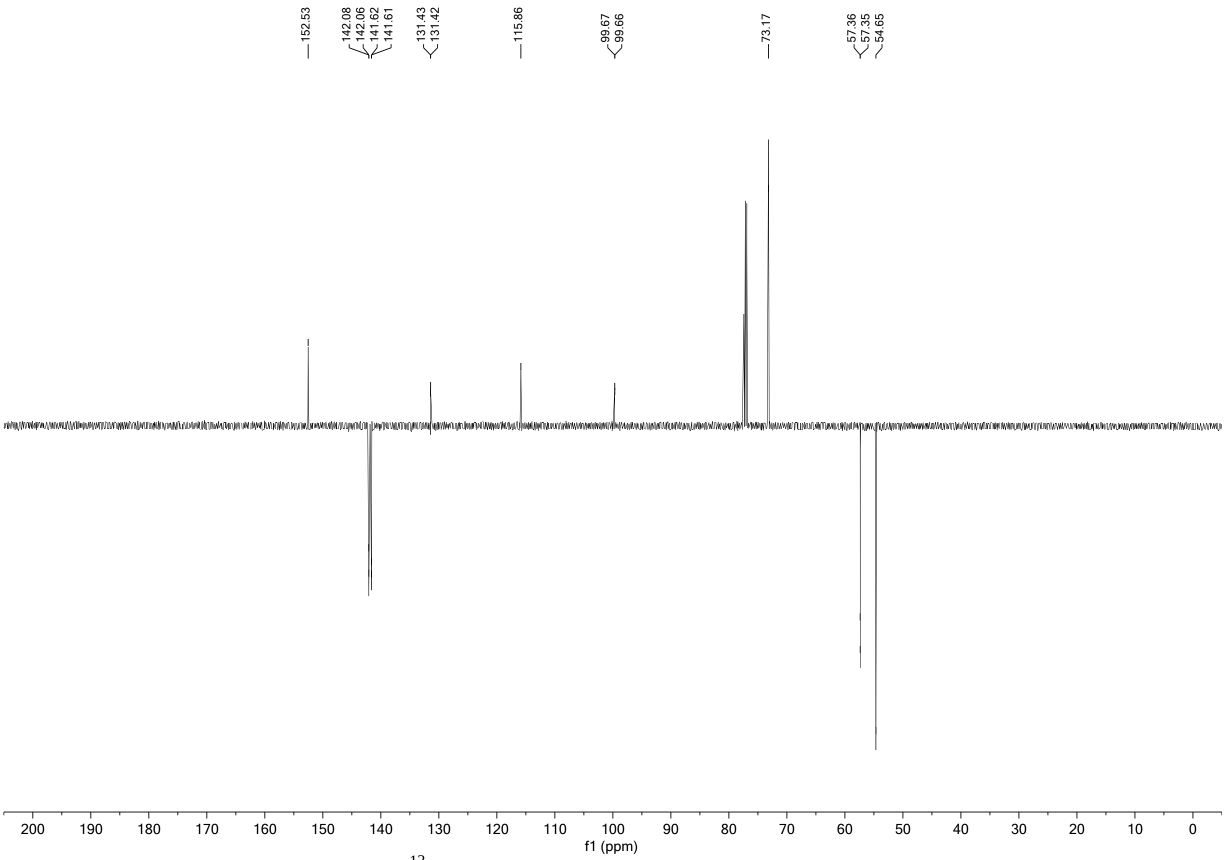

Supplementary Figure 13. NMR spectrum. ^{13}C NMR APT (126 MHz) of **5** in $CDCl_3$.

Compound 8:

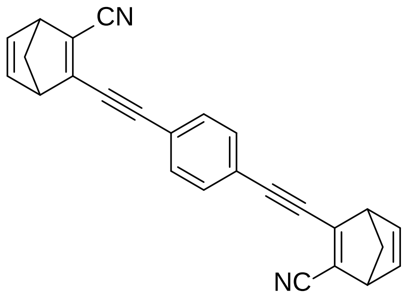

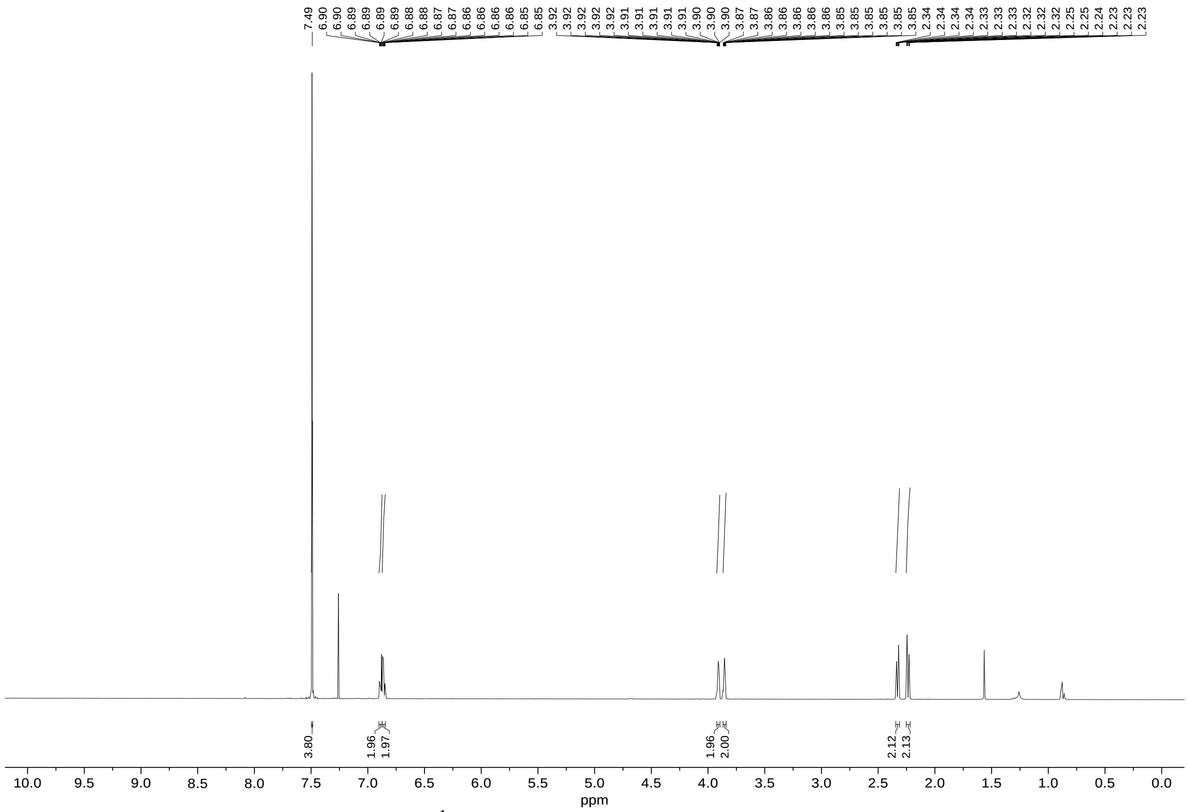

Supplementary Figure 14. NMR spectrum. ^{1}H NMR (400 MHz) of **8** in $CDCl_3$.

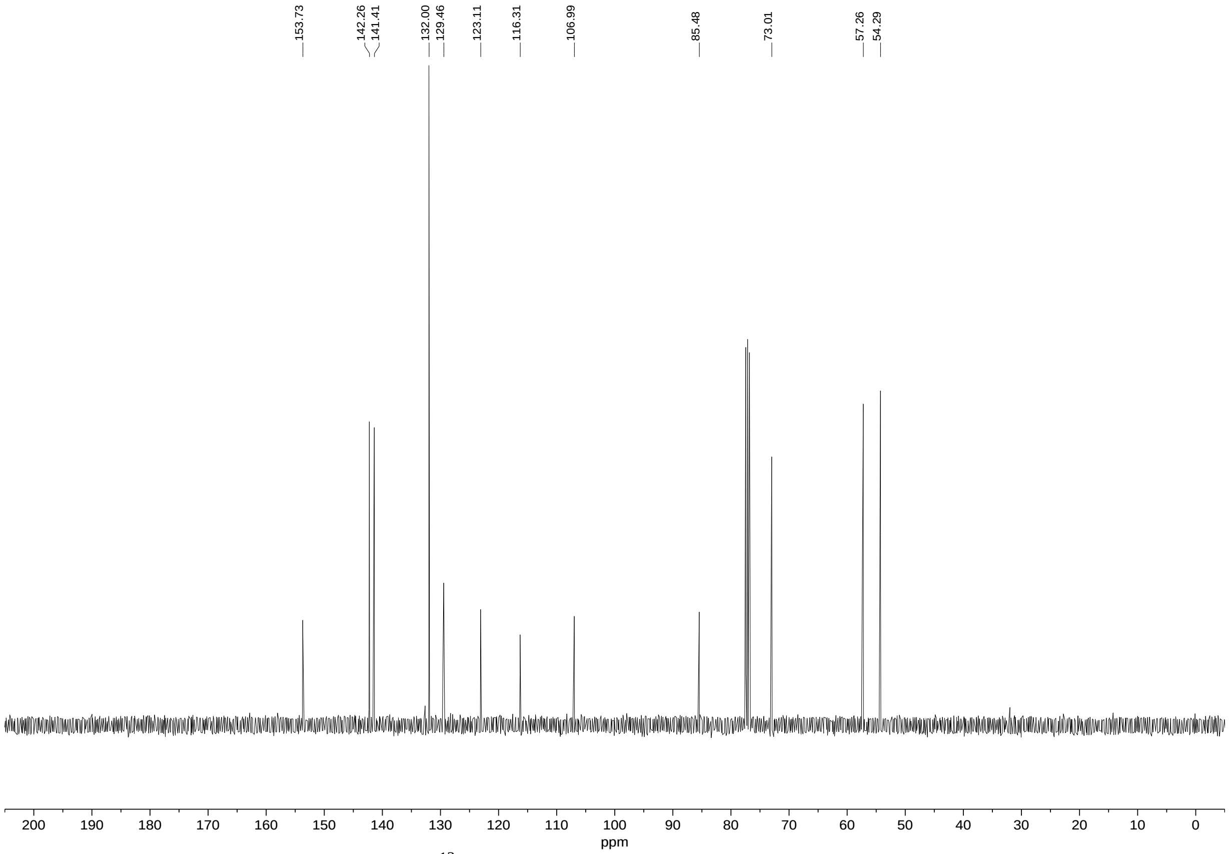

Supplementary Figure 15. NMR spectrum. ^{13}C NMR (100 MHz) of **8** in $CDCl_3$.

Compound 9:

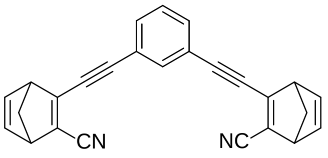

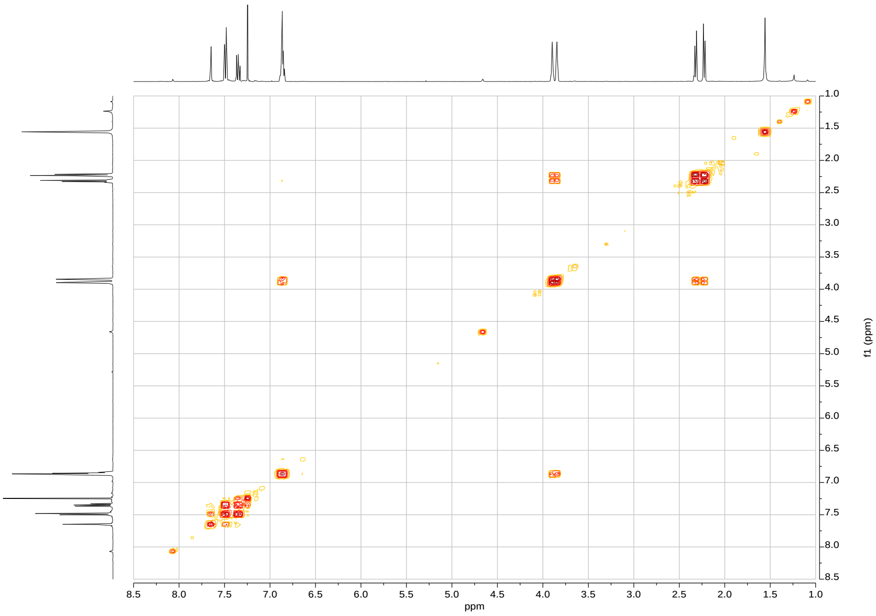

Supplementary Figure 16. NMR spectrum. COSY NMR (400 MHz) of **9** in $CDCl_3$.

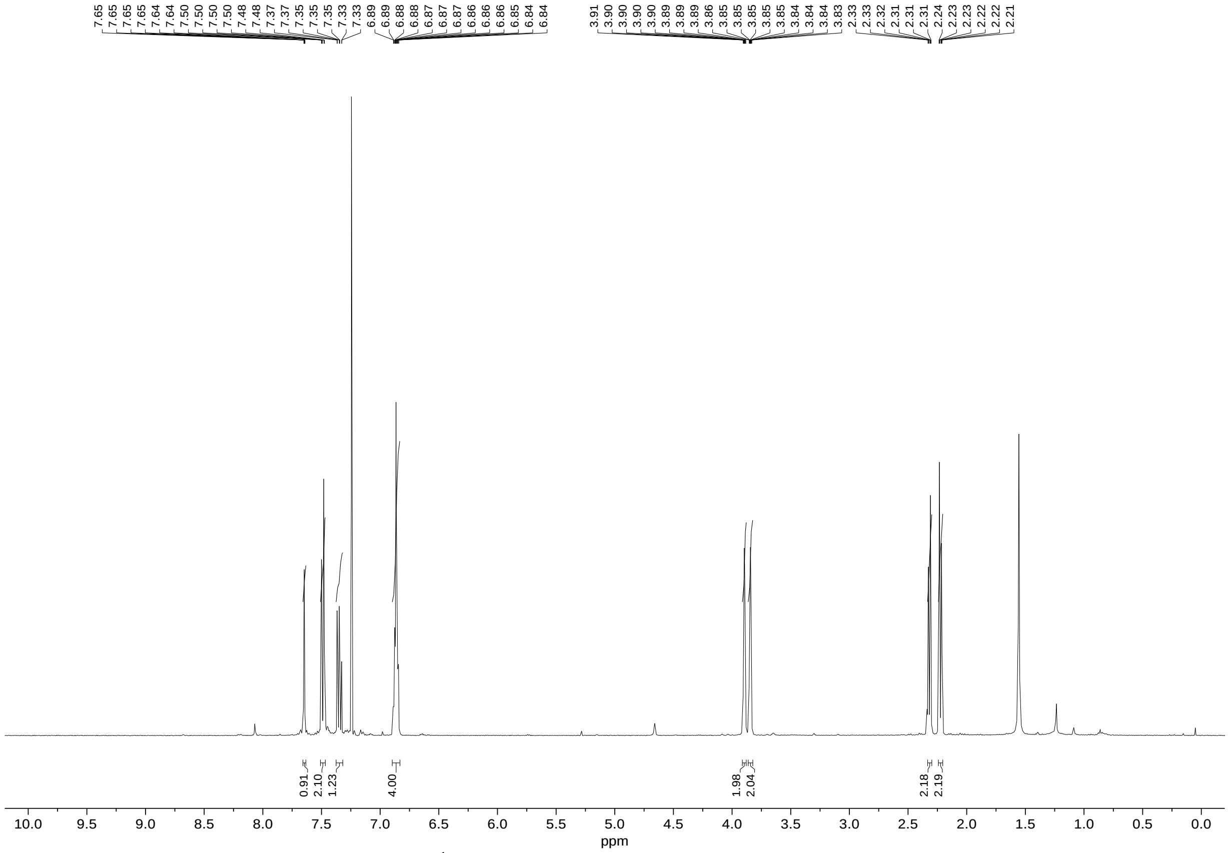

Supplementary Figure 17. NMR spectrum. ^1H NMR (400 MHz) of **9** in $CDCl_3$.

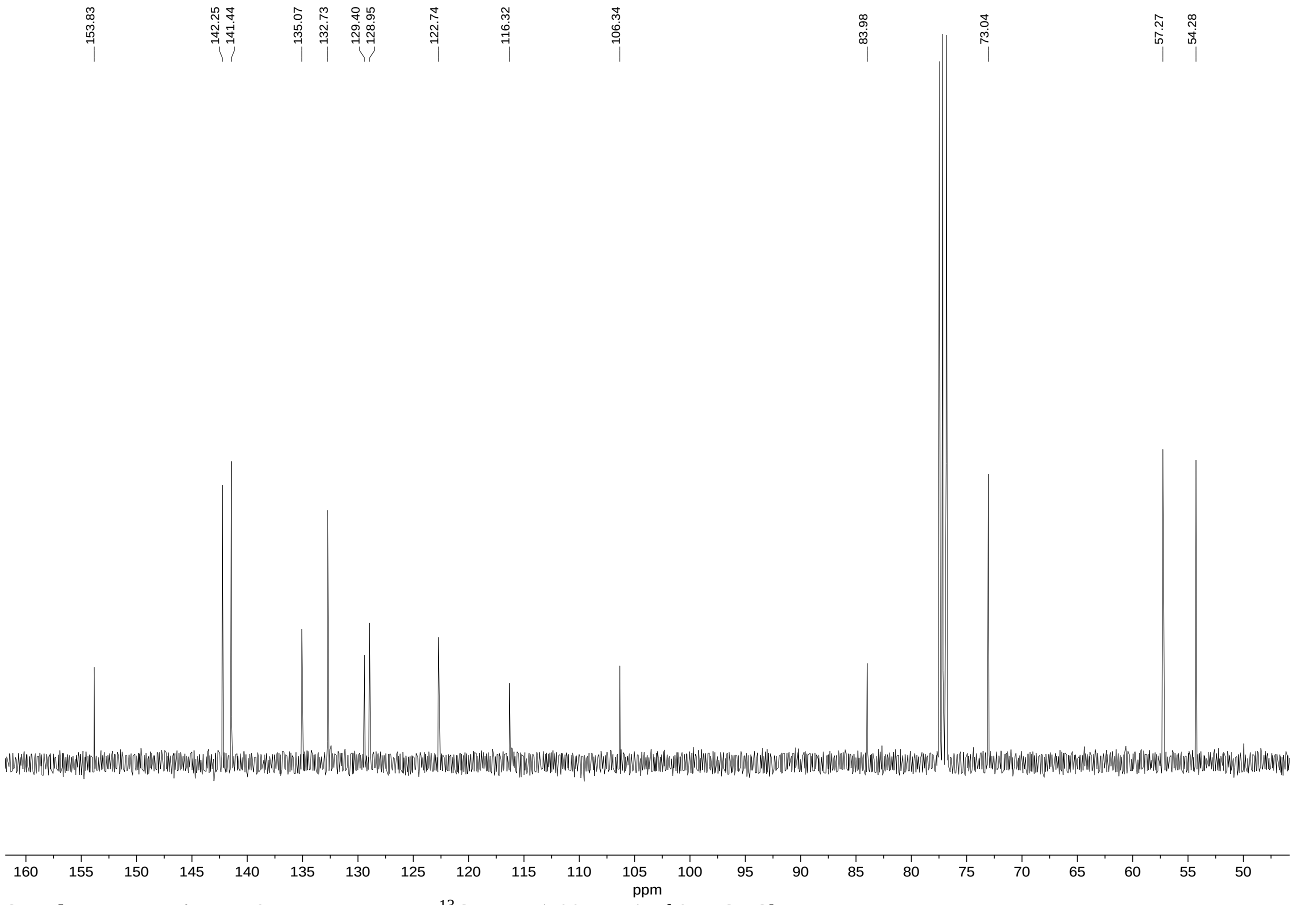

Supplementary Figure 18. NMR spectrum. ^{13}C NMR (100 MHz) of **9** in $CDCl_3$.

Compound 11

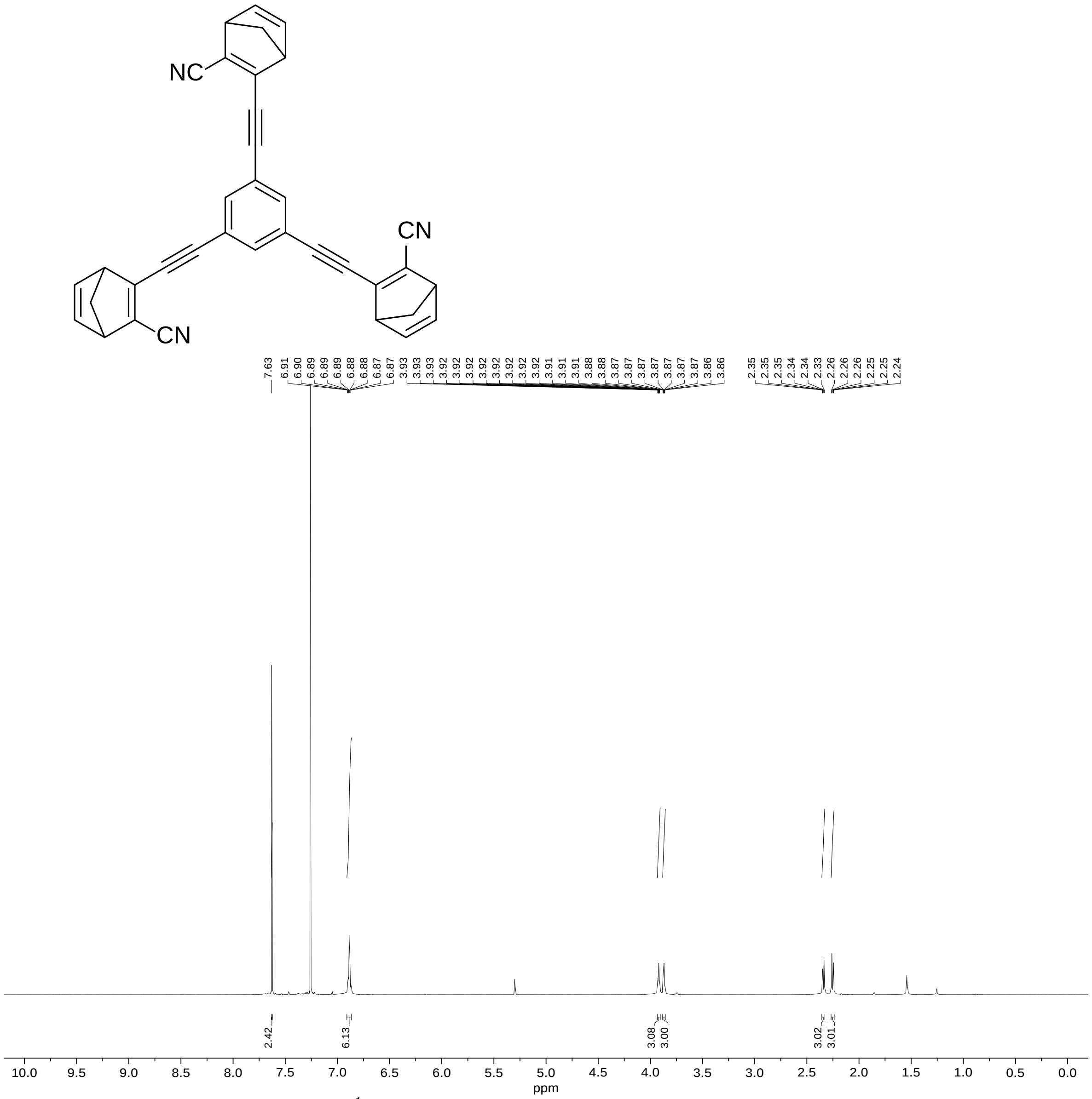

Supplementary Figure 19. NMR spectrum. ^{1}H NMR (500 MHz) of **11** in $CDCl_3$.

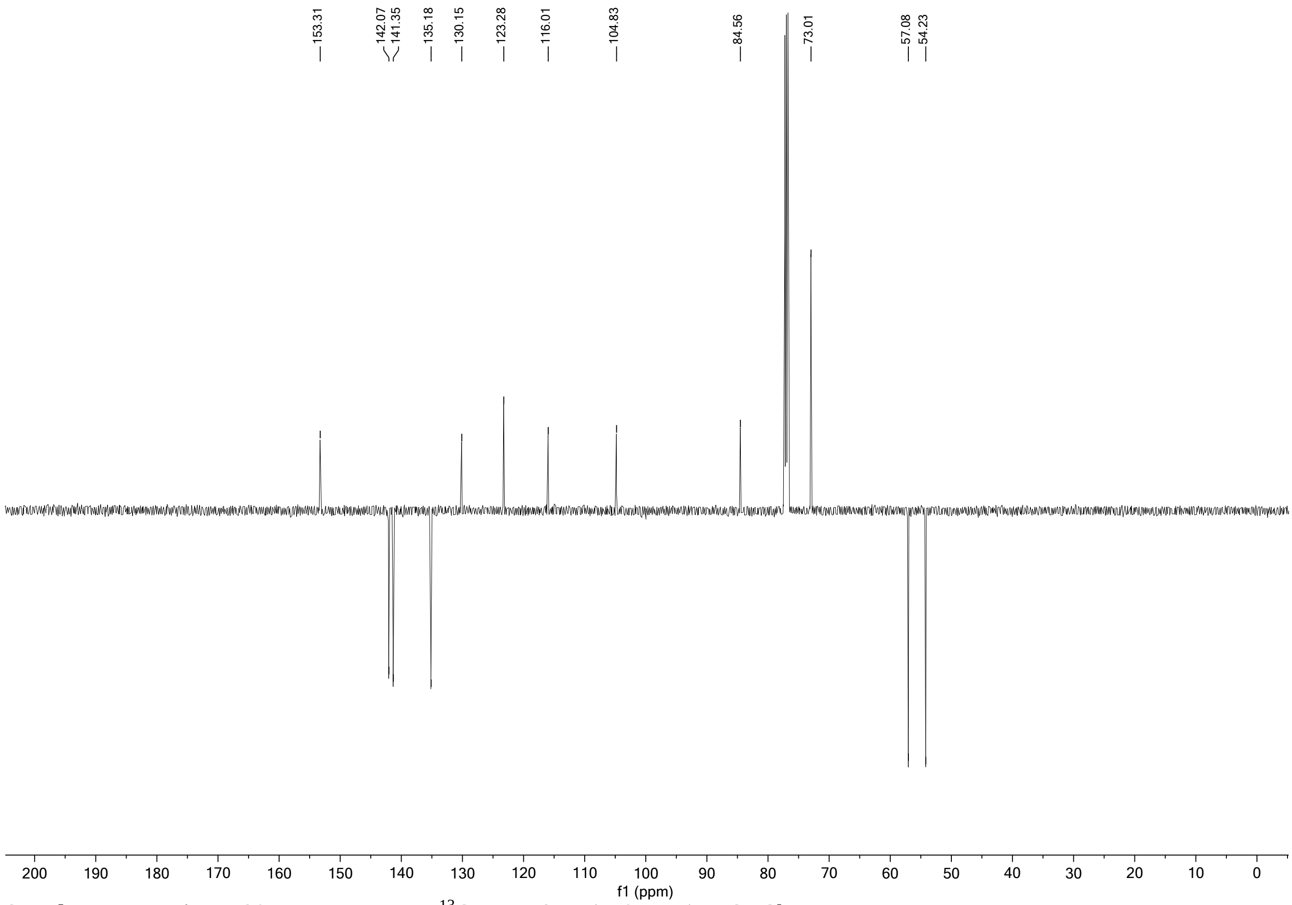

Supplementary Figure 20. NMR spectrum. ^{13}C NMR APT (126 MHz) in $CDCl_3$.

Compound 14

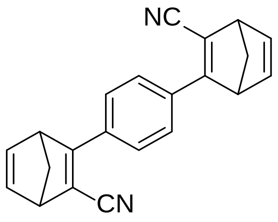

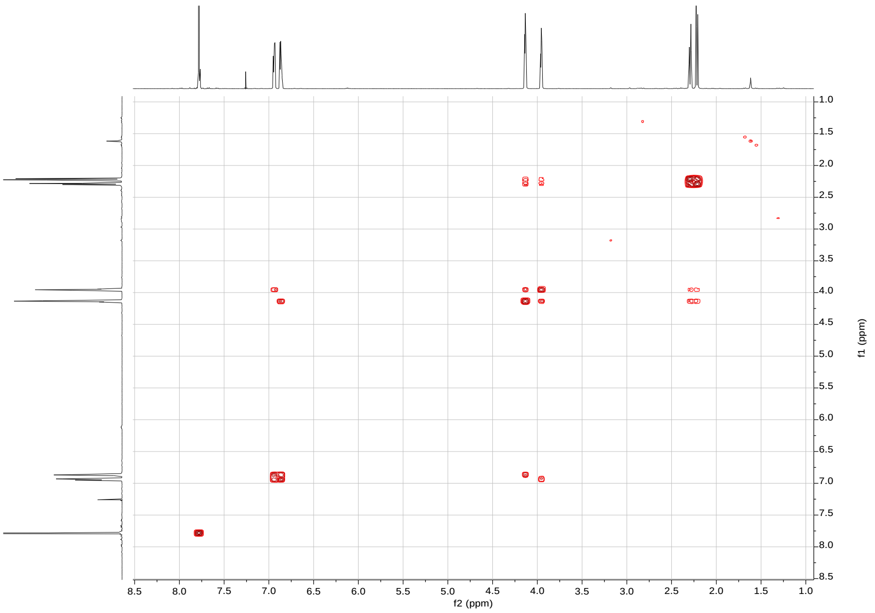

Supplementary Figure 21. NMR spectrum. COSY NMR (400 MHz) of **14** in $CDCl_3$.

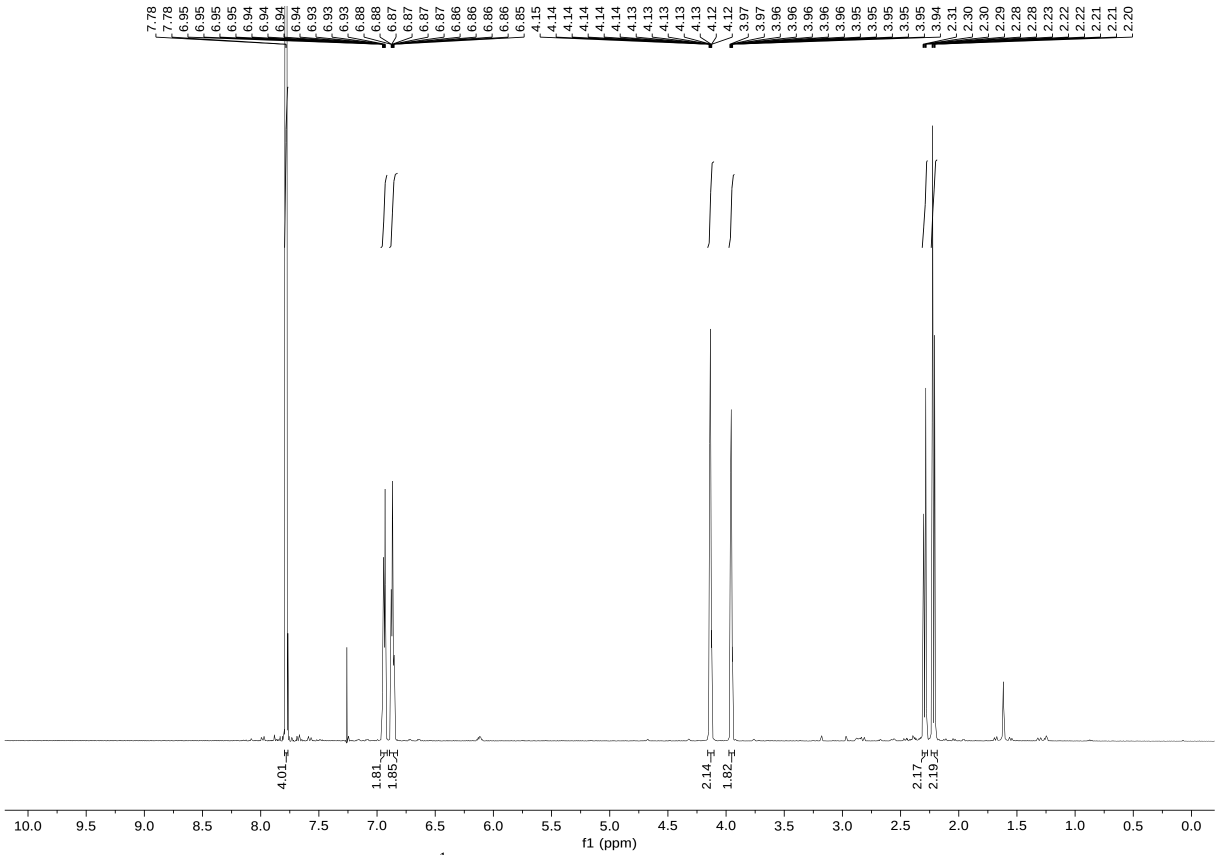

Supplementary Figure 22. NMR spectrum. ^{1}H NMR (400 MHz) of **14** in $CDCl_3$.

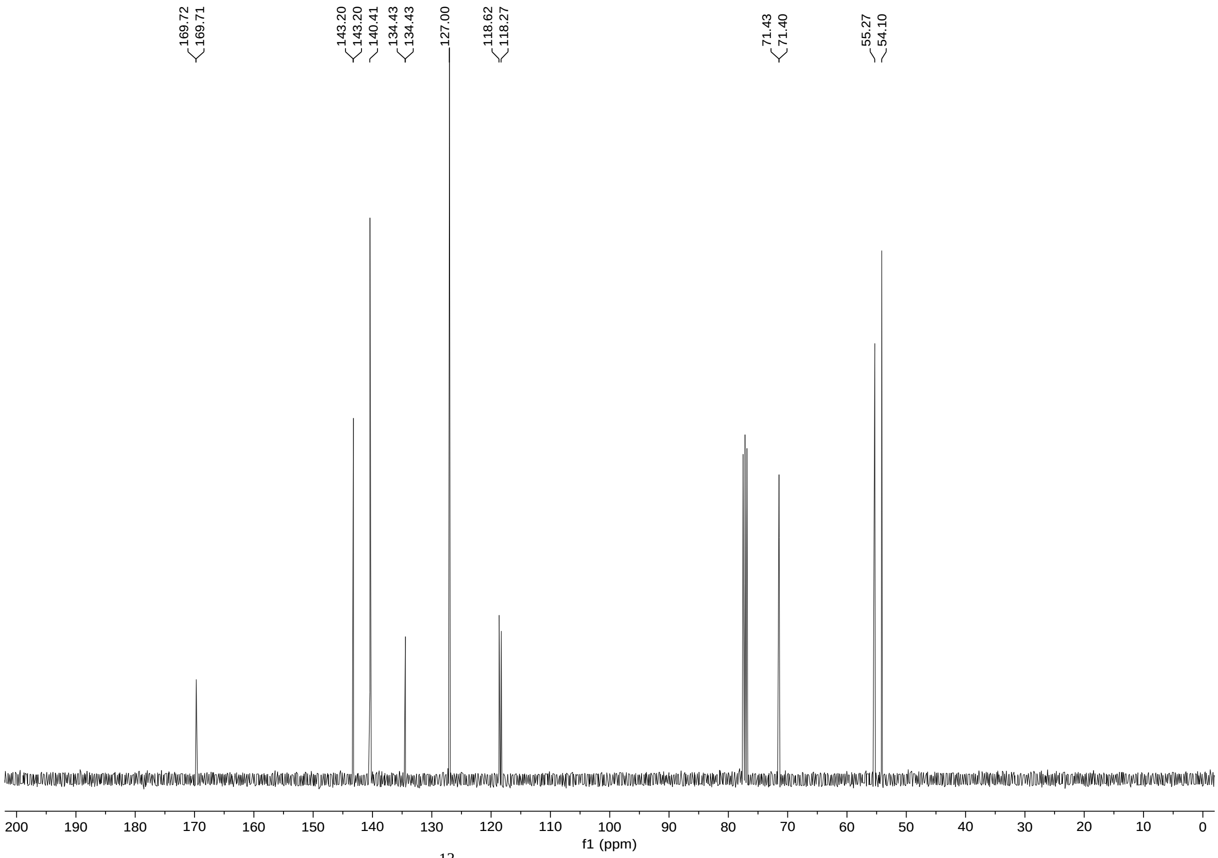

Supplementary Figure 23. NMR spectrum. ^{13}C NMR (100 MHz) of **14** in $CDCl_3$.

Compound 15

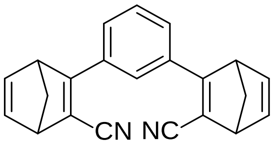

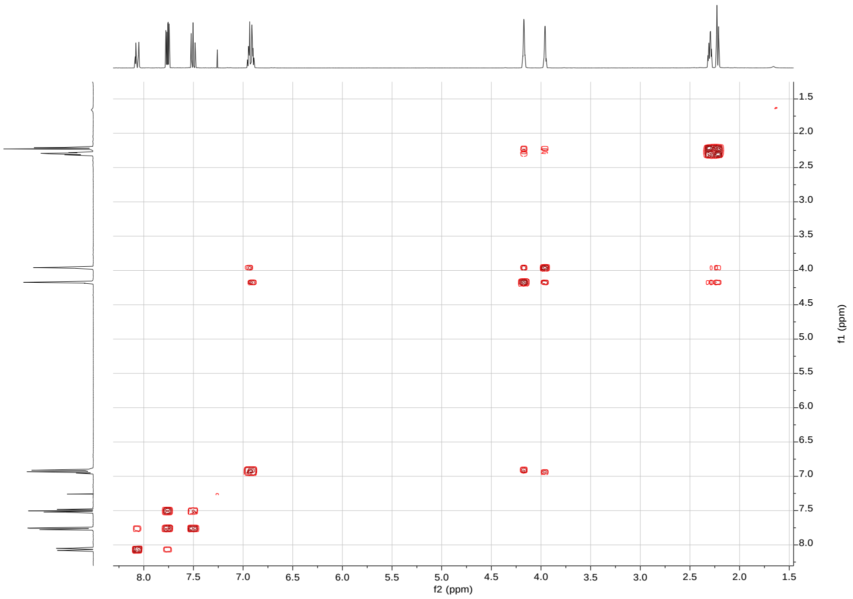

Supplementary Figure 24. NMR spectrum. COSY NMR (400 MHz) of **15** in $CDCl_3$.

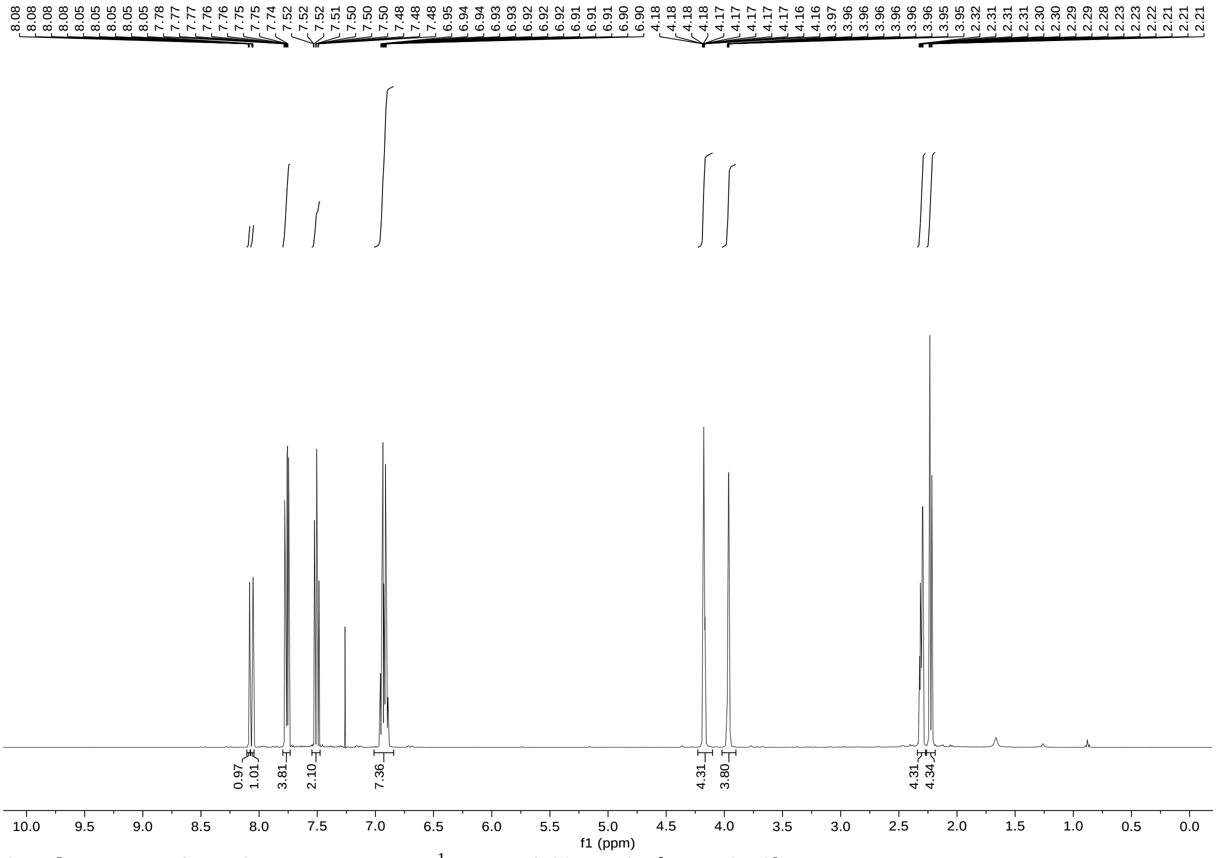

Supplementary Figure 25. NMR spectrum. ^{1}H NMR (400 MHz) of **15** in $CDCl_3$.

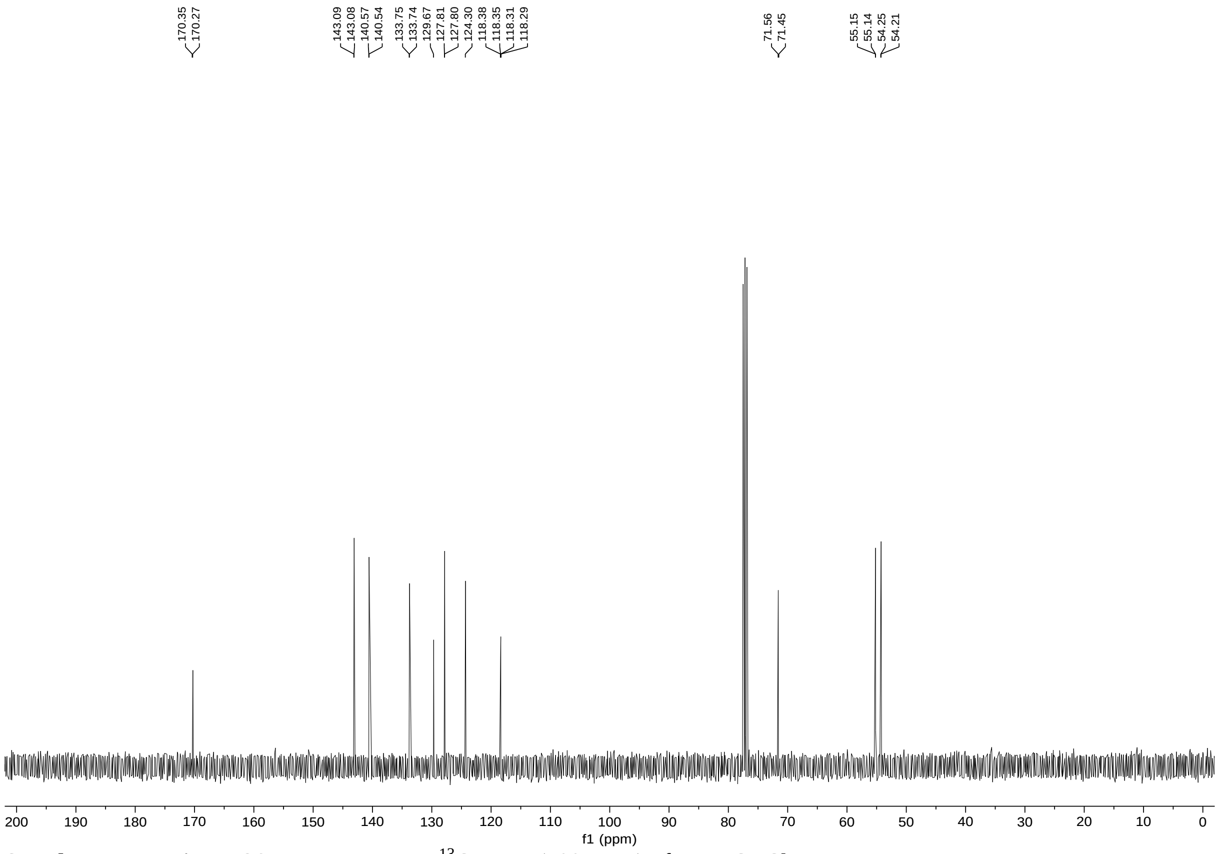

Supplementary Figure 26. NMR spectrum. ^{13}C NMR (100 MHz) of **15** in $CDCl_3$.

Compound $8_{QC\text{-}QC}$

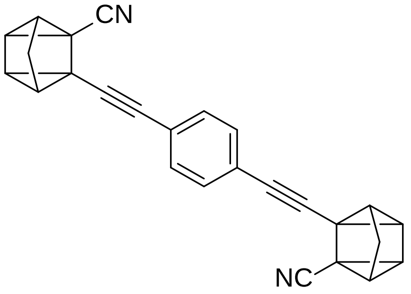

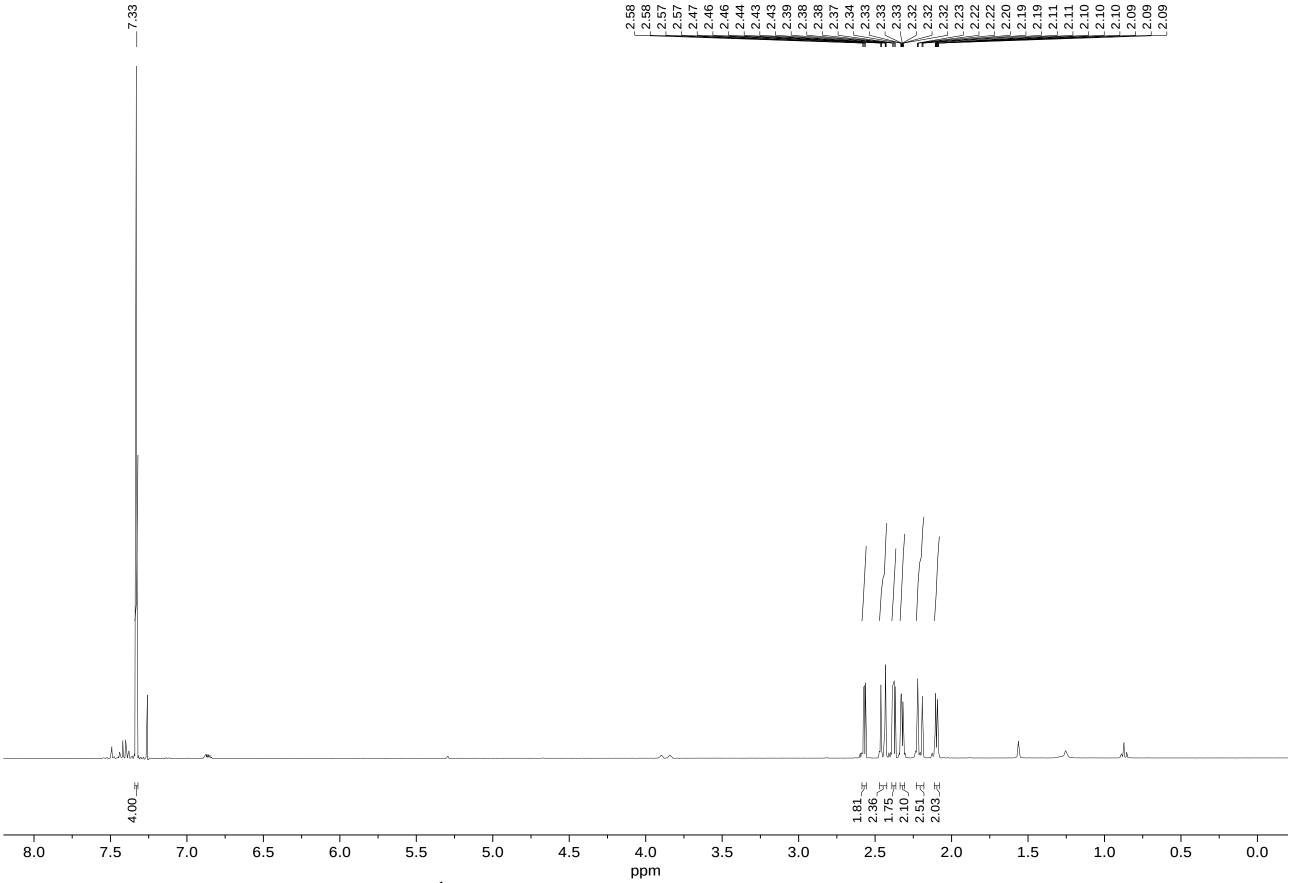

Supplementary Figure 27. NMR spectrum. ^{1}H NMR (400 MHz) of **$8_{QC\text{-}QC}$** in $CDCl_3$.

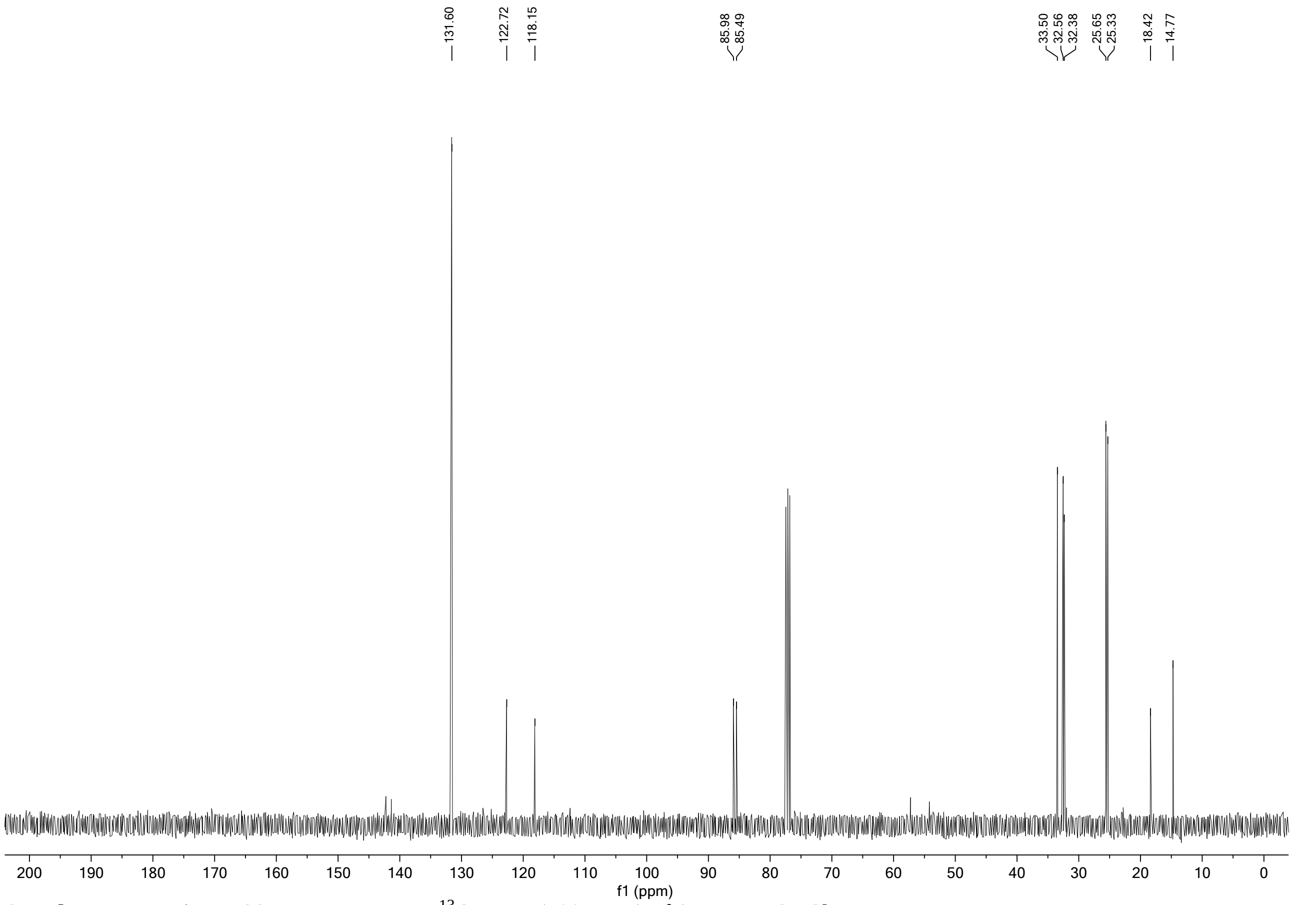

Supplementary Figure 28. NMR spectrum. ^{13}C NMR (100 MHz) of $\mathbf{8}_{\mathbf{QC\text{-}QC}}$ in $CDCl_3$.

Compound $9_{QC\text{-}QC}$

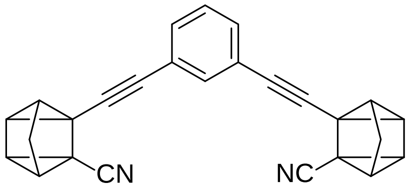

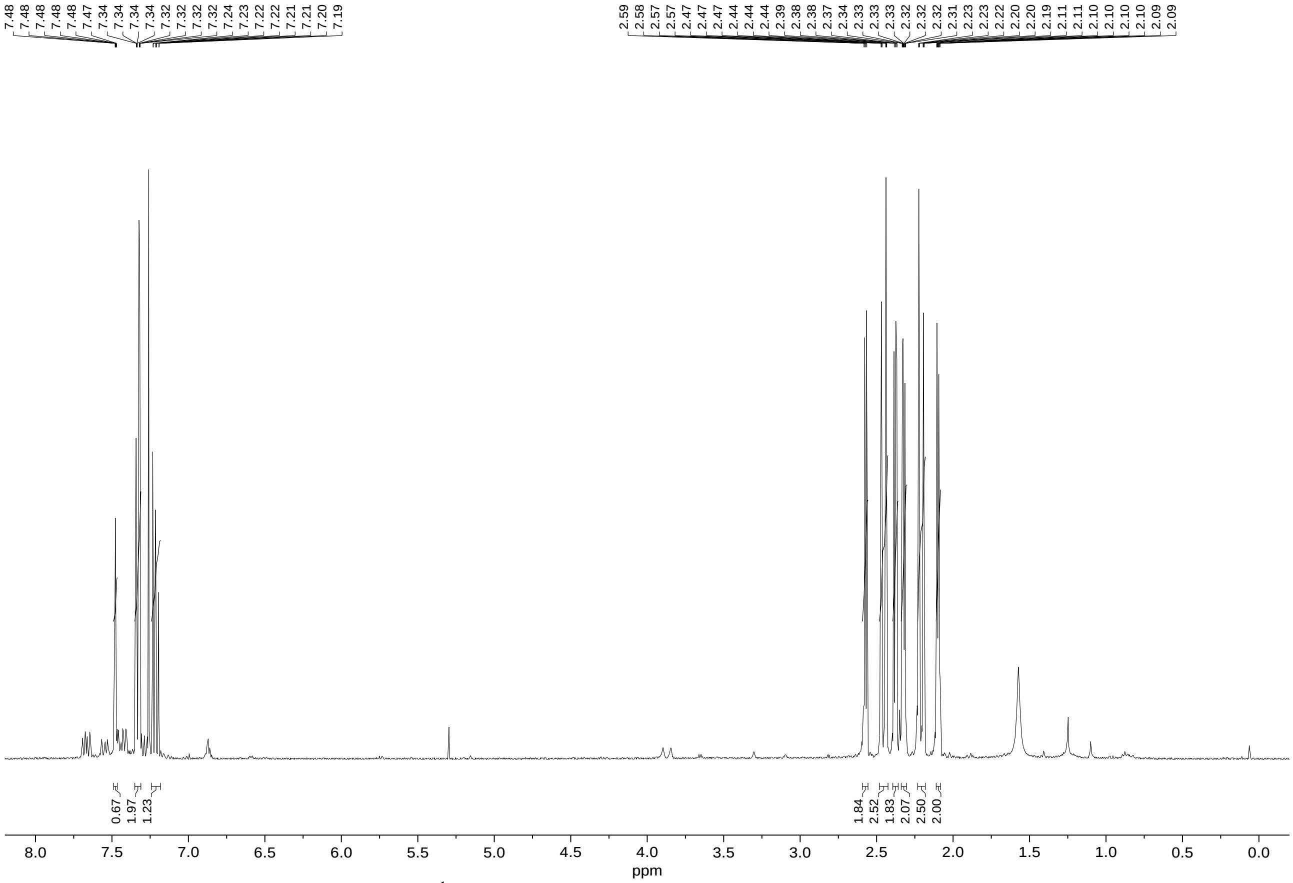

Supplementary Figure 29. NMR spectrum. ^{1}H NMR (400 MHz) of $\mathbf{9_{QC\text{-}QC}}$ in $CDCl_3$.

Compound $11_{QC\text{-}QC\text{-}QC}$

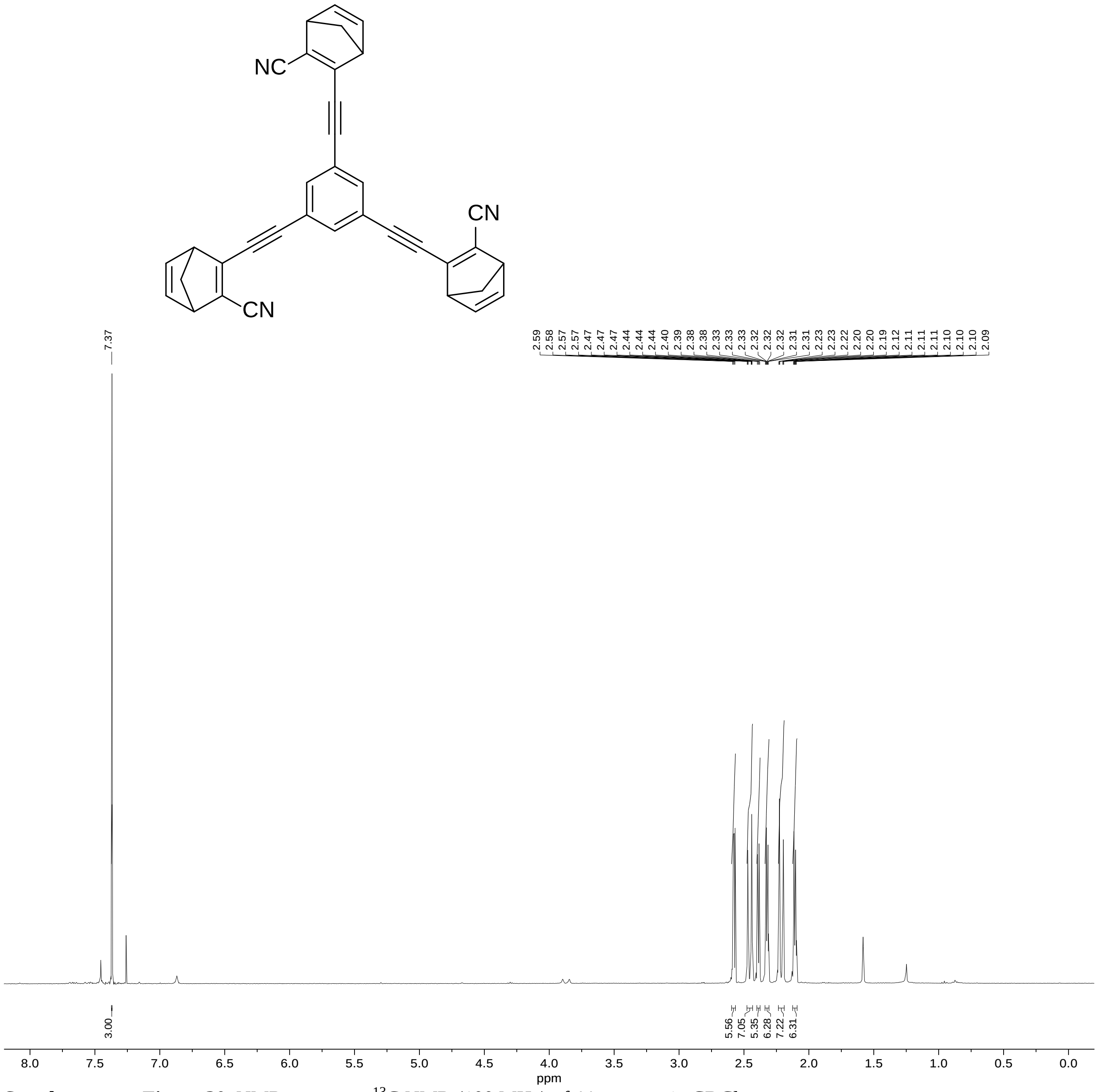

Supplementary Figure 30. NMR spectrum. ^{13}C NMR (100 MHz) of $\mathbf{11_{QC\text{-}QC\text{-}QC}}$ in $CDCl_3$.

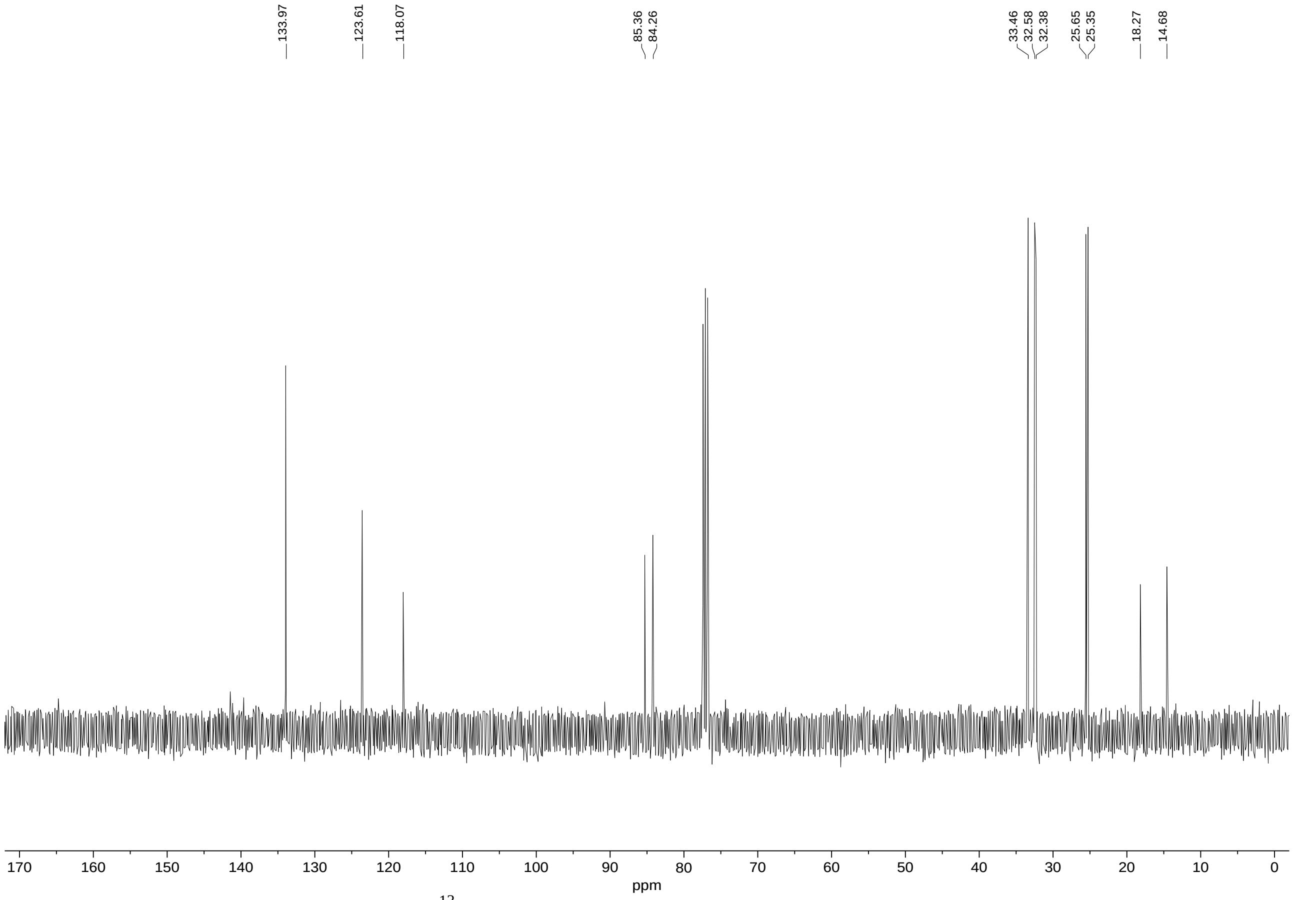

Supplementary Figure 31. NMR spectrum. ^{13}C NMR (100 MHz) of $\mathbf{11_{QC\text{-}QC\text{-}QC}}$ in $CDCl_3$.

Compound 14_{QC-QC}

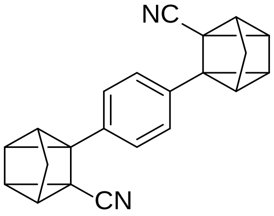

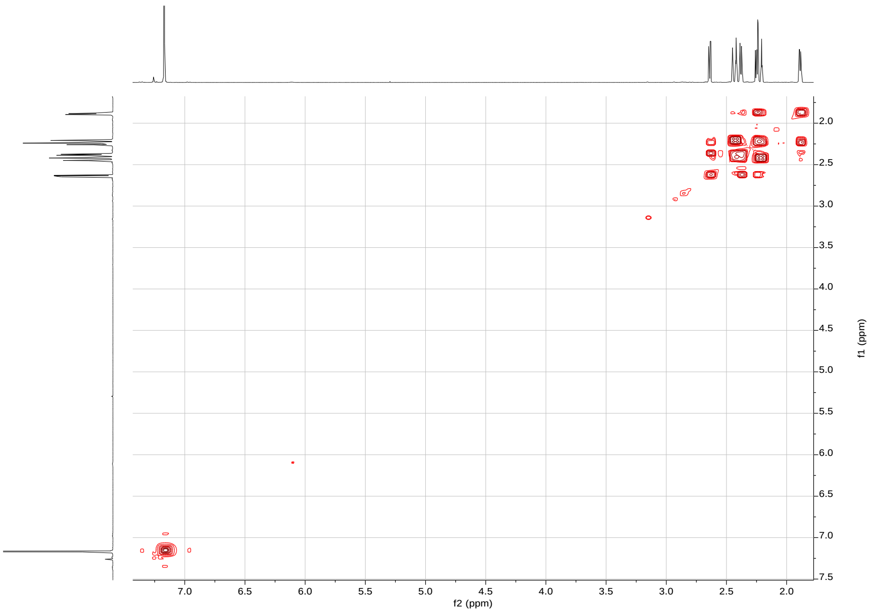

Supplementary Figure 32. NMR spectrum. COSY NMR (400MHz) of $\mathbf{14}_{\mathbf{QC\text{-}QC}}$ in $CDCl_3$.

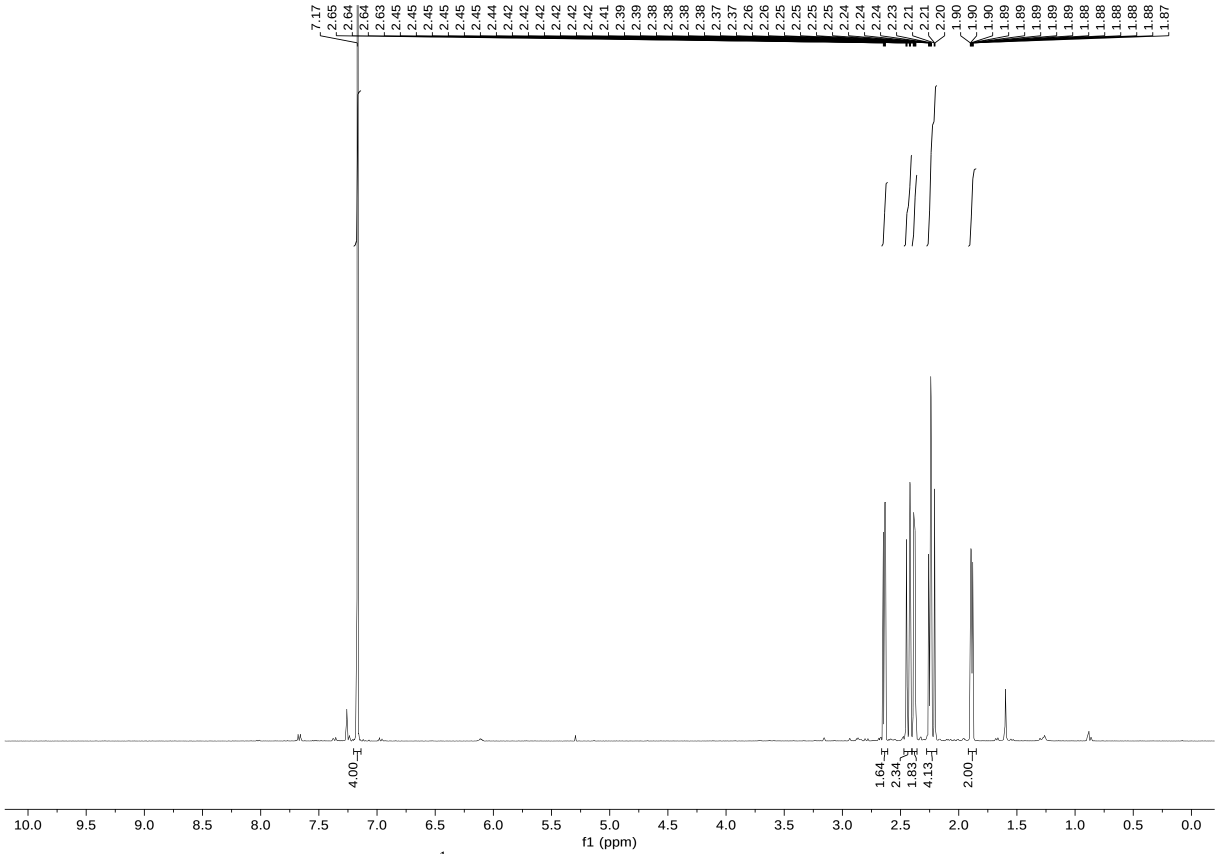

Supplementary Figure 33. NMR spectrum. ^{1}H NMR (400 MHz) of $\mathbf{14}_{\mathbf{QC\text{-}QC}}$ in $CDCl_3$.

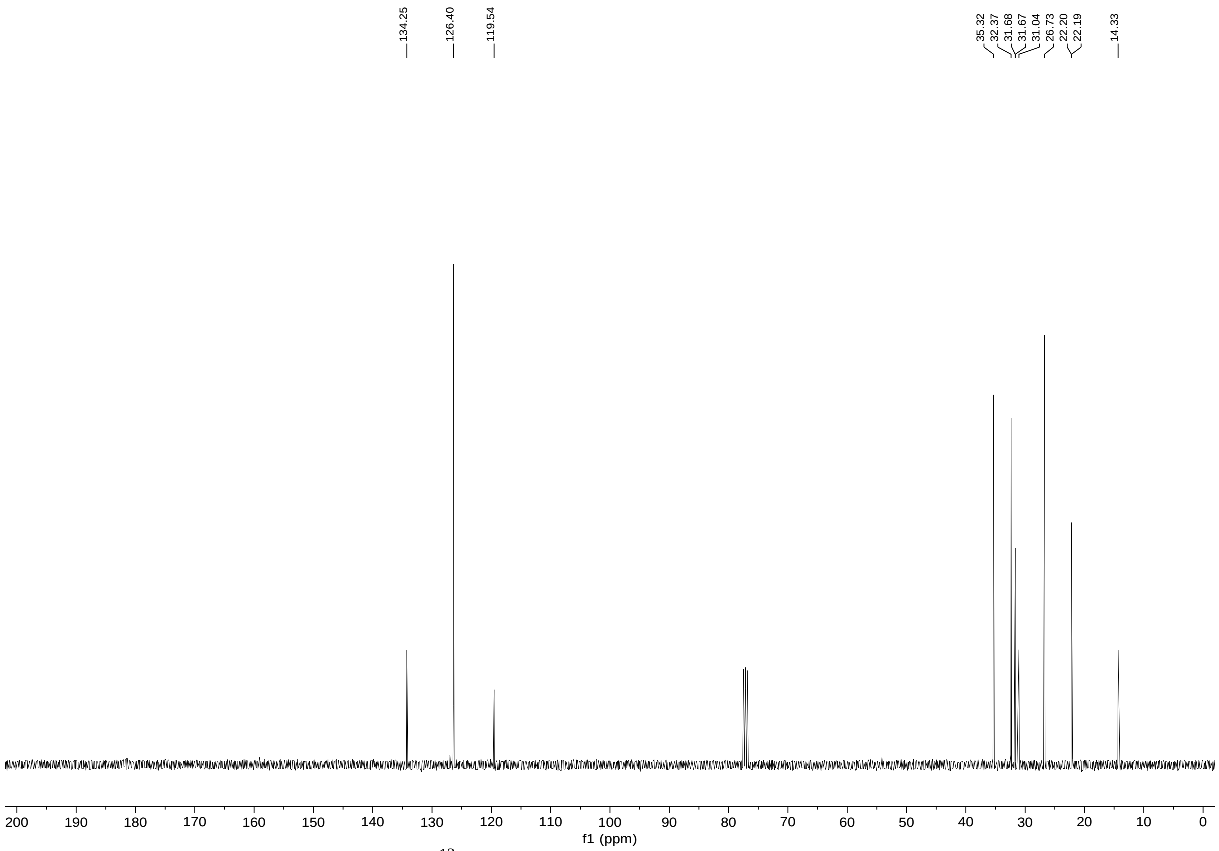

Supplementary Figure 34. NMR spectrum. ^{13}C NMR (100 MHz) of $\mathbf{14_{QC\text{-}QC}}$ in $CDCl_3$.

Compound $15_{QC\text{-}QC}$

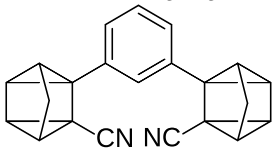

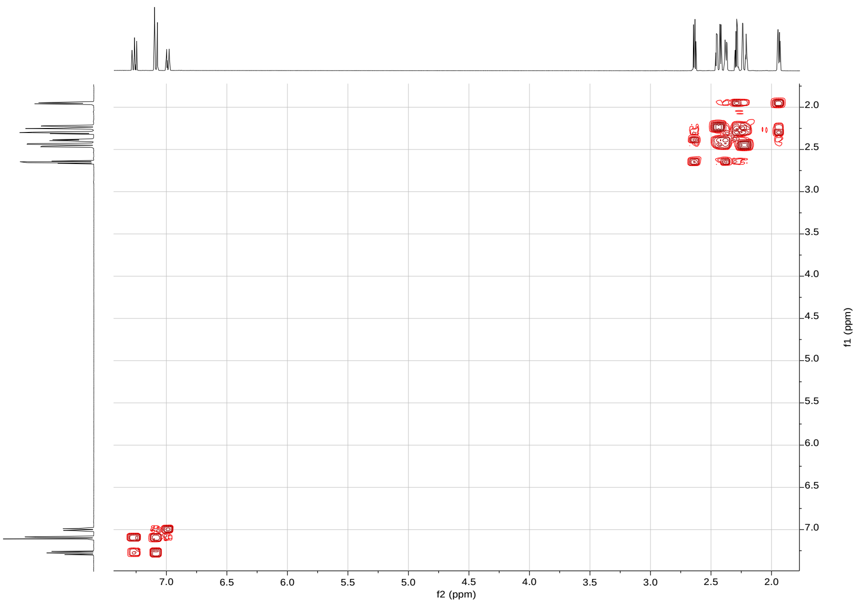

Supplementary Figure 35. NMR spectrum. COSY NMR (400 MHz) of $\mathbf{15_{QC\text{-}QC}}$ in $CDCl_3$.

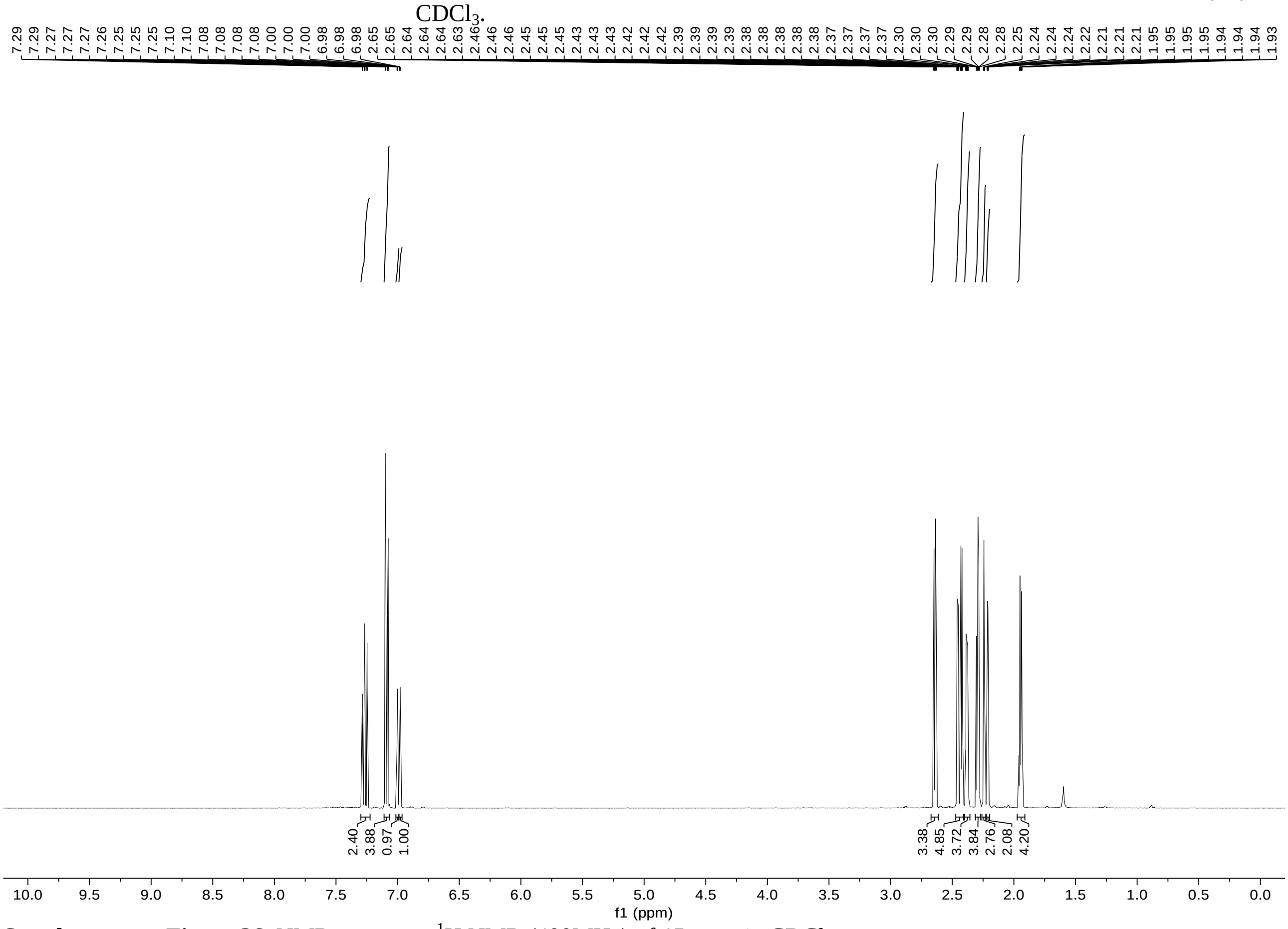

Supplementary Figure 36. NMR spectrum. ^{1}H NMR (400MHz) of $\mathbf{15_{QC\text{-}QC}}$ in $CDCl_3$.

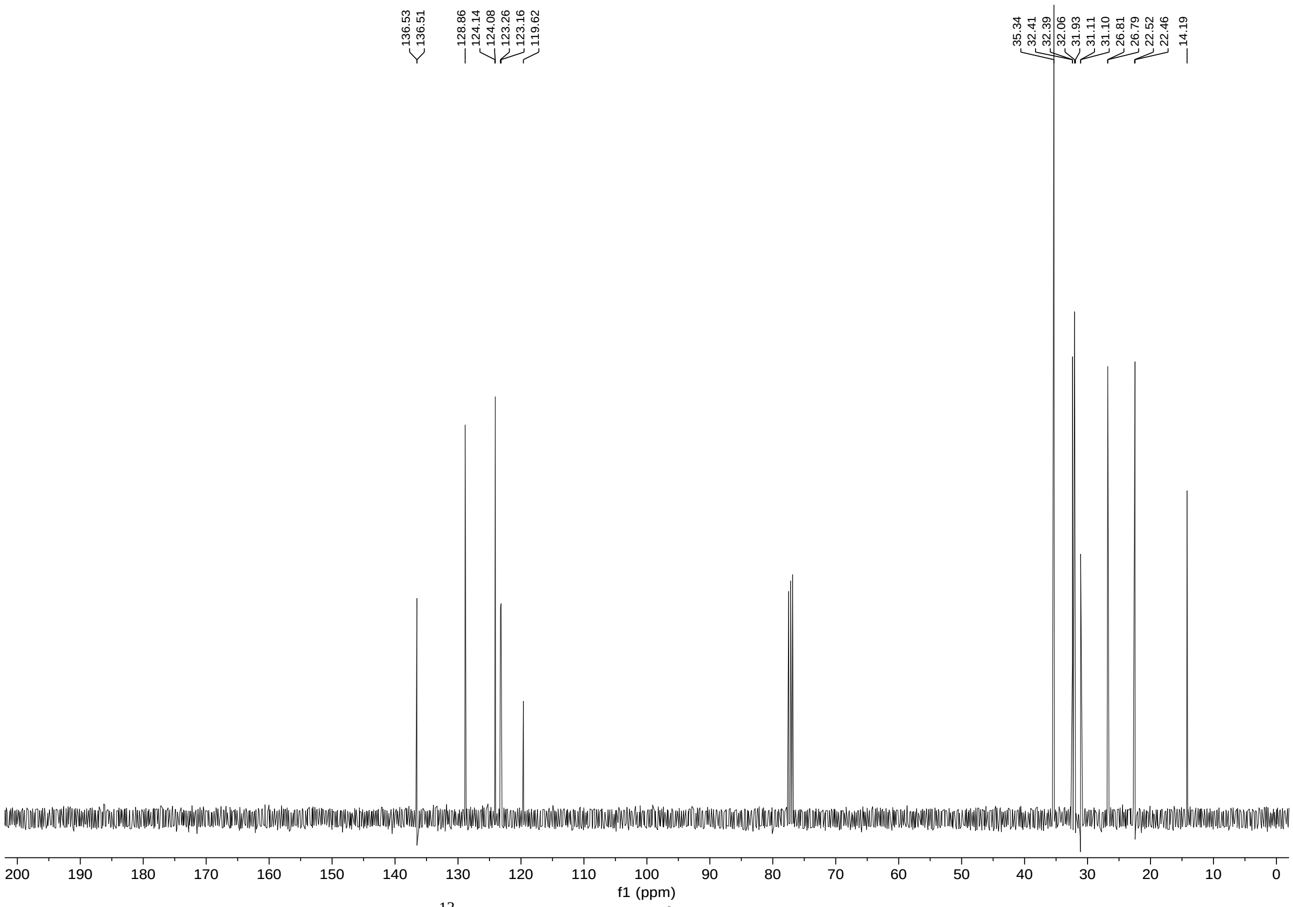

Supplementary Figure 37. NMR spectrum. ^{13}C NMR (100 MHz) of $\mathbf{15_{QC\text{-}QC}}$ in in $CDCl_3$.

UV-Vis Absorption and Switching Studies

Compound 5:

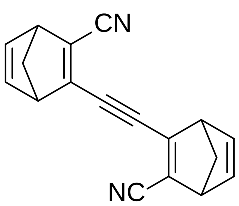

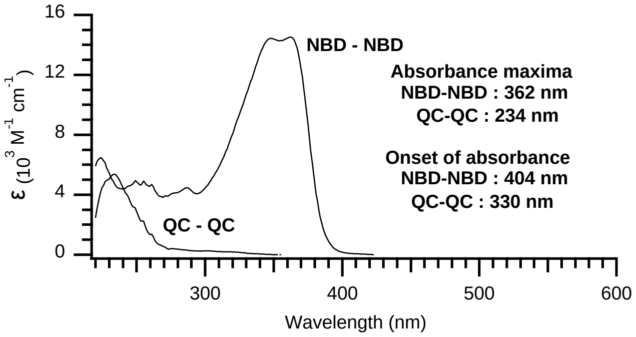

Supplementary Figure 38. UV-Vis spectra. UV-Vis spectra of **5** and $\mathbf{5}_{\mathbf{QC\text{-}QC}}$ in cyclohexane.

Supplementary Note 1. Extinction coefficient for the NBD at absorbance maximum (362 nm) calculated as the average of three solutions (15372, 14515 and 14441) giving 14776 $M^{-1}cm^{-1}$.

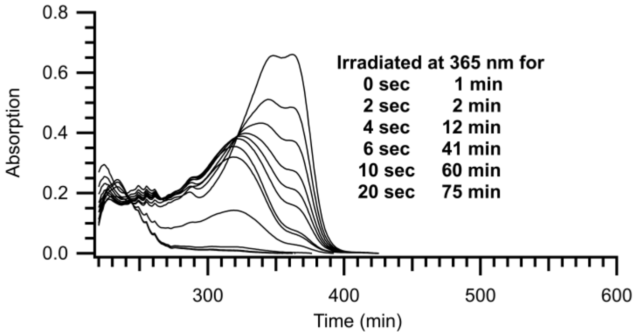

Supplementary Figure 39. UV-Vis spectra. UV-Vis spectra of **5** after irradiation after different irradiation times at 365 nm. Concentration in the cuvette: 3.73 x 10^{-5} M.

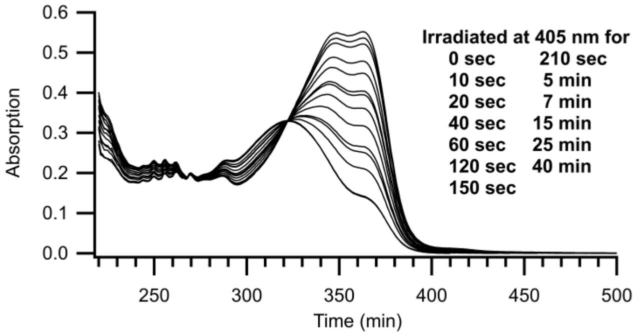

Supplementary Figure 40. UV-Vis spectra. UV-Vis spectra of **5** after irradiation after different irradiation times at 405 nm. Concentration in the cuvette: 4.47 x 10^{-5} M

.

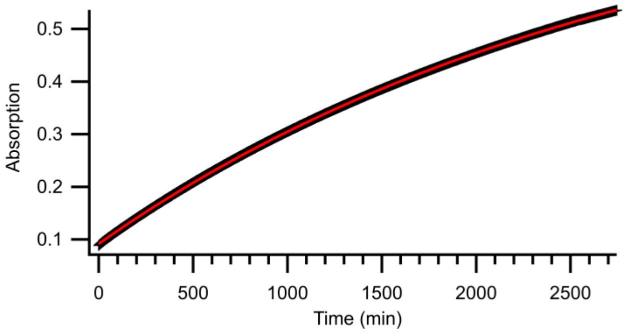

Supplementary Figure 41. Kinetics. Increase at absorbance maximum (362 nm) of **5** at 29.9 °C during the thermal backreaction.

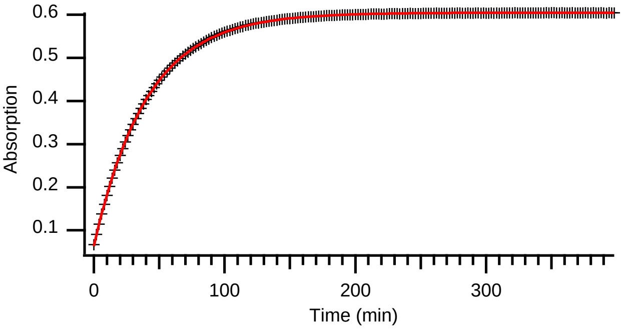

Supplementary Figure 42. Kinetics. Increase at absorbance maxium (362 nm) of NBD at 59.7 °C during the thermal backreaction.

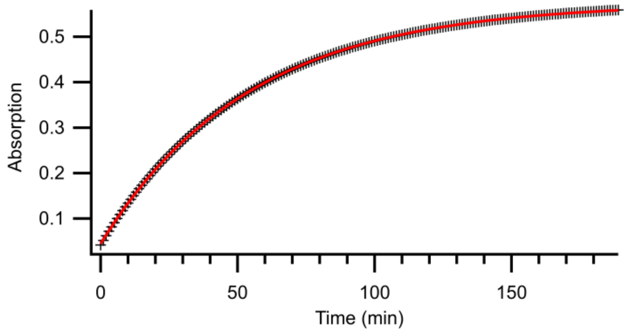

Supplementary Figure 43. Kinetics. Increase at absorbance maximum (362 nm) of NBD at 44.9 °C during the thermal backreaction.

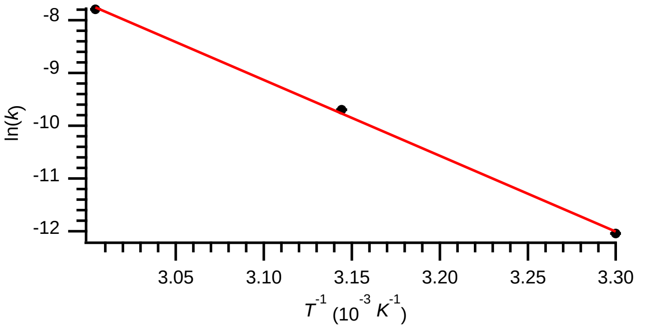

Supplementary Figure 44. Arrhenius plot. Arrhenius plot for **5** giving the values A = $\mathbf{2.599^{+7.131}_{-1.905}}$ x 10^{15} s^{-1} Ea = 119695 ± 3475 J mol^{-1}.

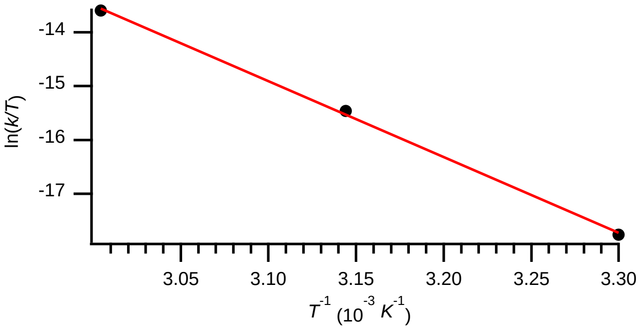

Supplementary Figure 45. Eyring plot. Eyring plot for **5** giving the values $\Delta H^{\ddagger}$ = 117 ± 3.51 kJ mol^{-1}, $\Delta S^{\ddagger}$ = 41.4 ± 11.1 J mol^{-1} K^{-1}.

Compound $5_{QC\text{-}NBD}$

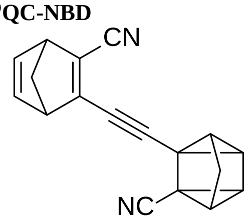

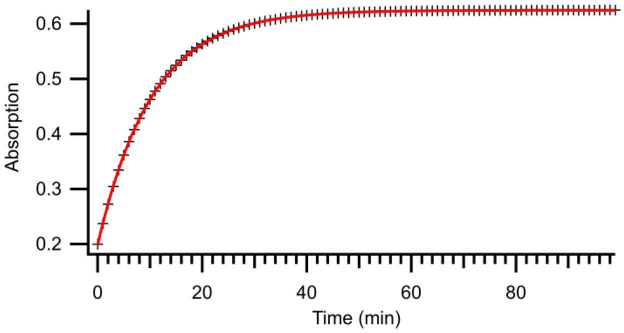

Supplementary Figure 46. Kinetics. Increase at absorbance maximum (362 nm) of **5** at 25.1 °C during the thermal backreaction, after 405 nm irradiation.

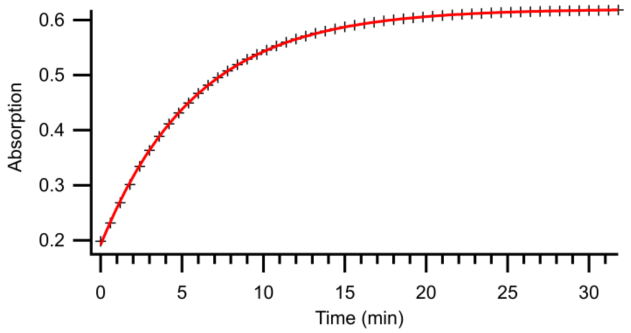

Supplementary Figure 47. Increase at absorbance maximum (362 nm) of **5** at 29.9 °C during the thermal backreaction, after 405 nm irradiation.

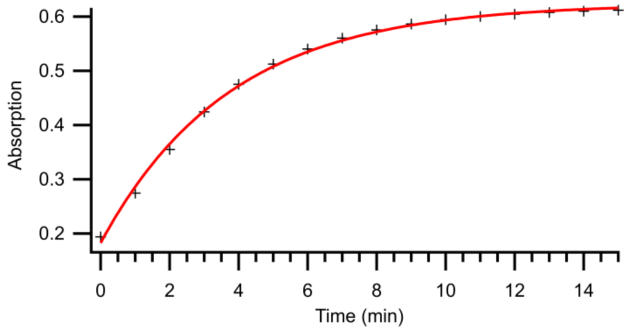

Supplementary Figure 48. Kinetics. Increase at absorbance maximum (362 nm) of **5** at 34.9 °C during the thermal backreaction, after 405 nm irradiation.

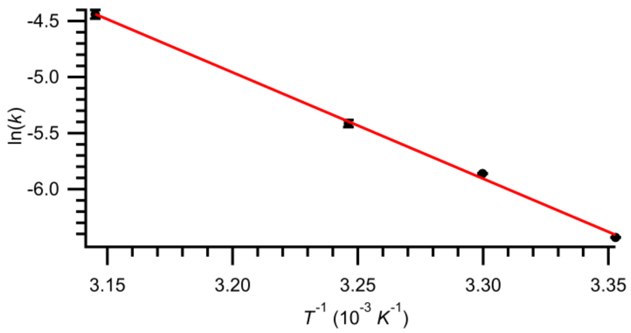

Supplementary Figure 49. Arrhenius plot. Arrhenius plot giving the values A = $\mathbf{1.069^{+1.303}_{-0.587}}$ x 10^{11} ± 2.219 s^{-1} Ea = 78864 ± 2029 J mol^{-1}.

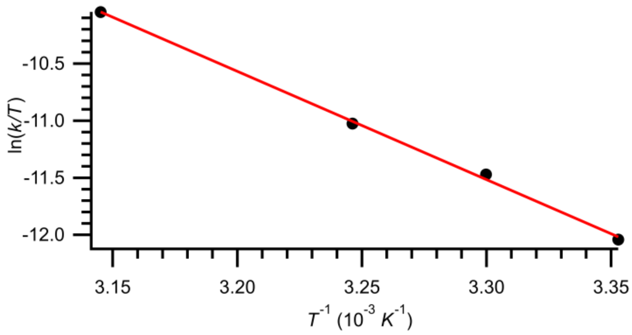

Supplementary Figure 50. Eyring plot. Eyring plot giving the values $\Delta H^{‡}$ = 76.3 ± 2.04 kJ mol^{-1}, $\Delta S^{‡}$ = -42.4 J ± 6.65 mol^{-1} K^{-1}.

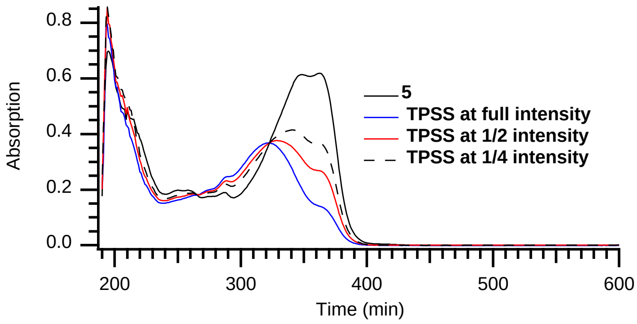

Supplementary Figure 51. Thermal-photostationary state. The different thermal-photostationary states (TPSS) of **5**, when irradiated with varying intensity of a 405 nm diode

Compound 8:

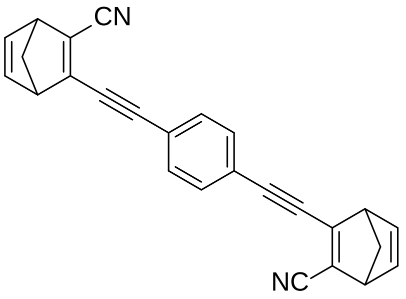

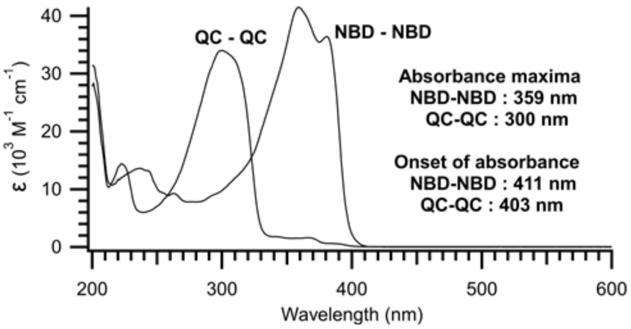

Supplementary Figure 52. UV-Vis spectra. UV-Vis spectra of **8** and $\mathbf{8}_{\mathbf{QC\text{-}QC}}$ in cyclohexane**.**

Supplementary Note 2. Extinction coefficient for the NBD at absorbance maximum (359 nm) calculated as the average of three solutions (41496, 40792 and 40726) giving 41005 $M^{-1}cm^{-1}$.

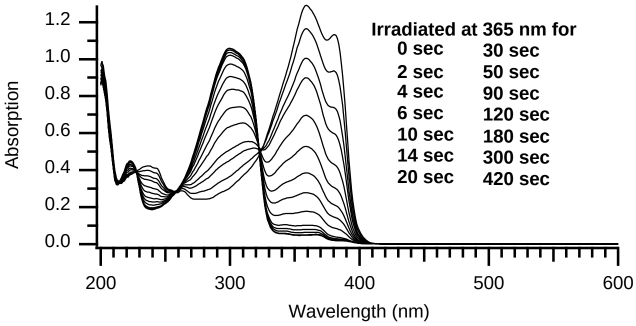

Supplementary Figure 53. UV-Vis spectra. UV-Vis spectra of **8** after irradiation at different irradiation times with 365 nm light. Concentration in the cuvette: 3.15 x 10^{-5} M.

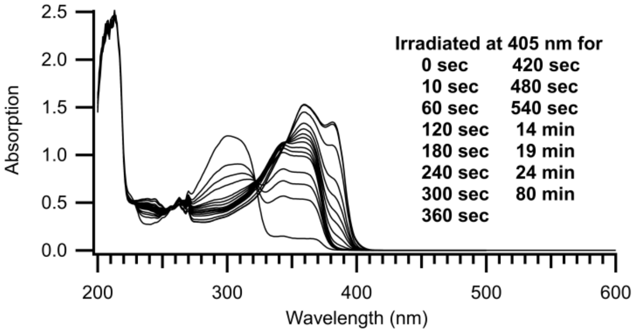

Supplementary Figure 54. UV-Vis spectra. UV-Vis spectra of **8** after irradiation at different irradiation times with 405 nm light. Concentration in the cuvette: 3.73 x 10^{-5} M.

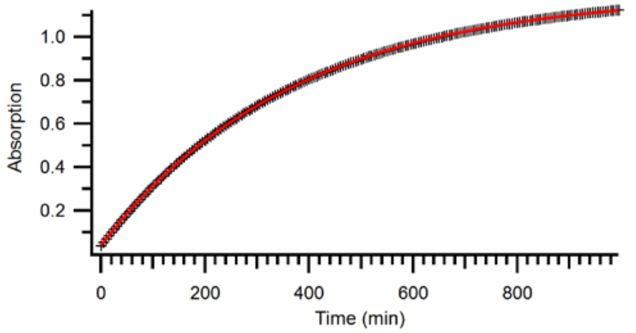

Supplementary Figure 55. Kinetics. Increase at absorbance maximum (362 nm) of **8** at 25 °C during the thermal backreaction.

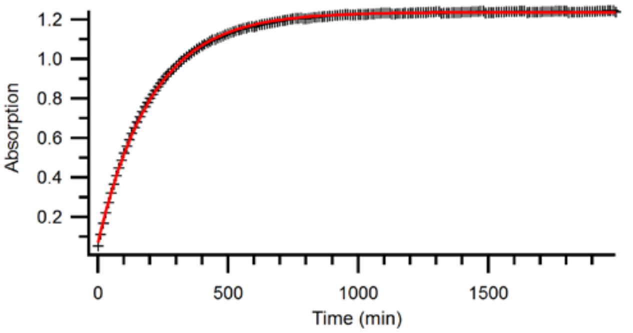

Supplementary Figure 56. Kinetics. Increase at absorbance maximum (362 nm) of **8** at 29.9 °C during the thermal backreaction.

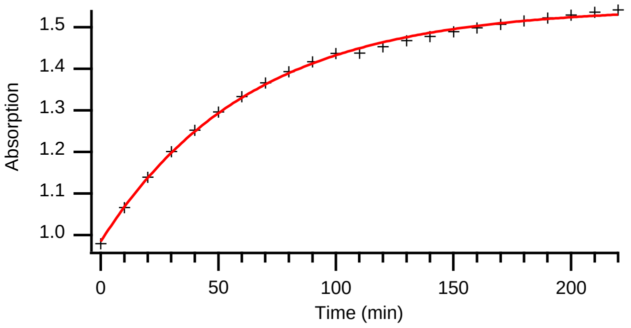

Supplementary Figure 57. Kinetics. Increase at absorbance maximum (362 nm) of **8** at 39.8 °C during the thermal backreaction.

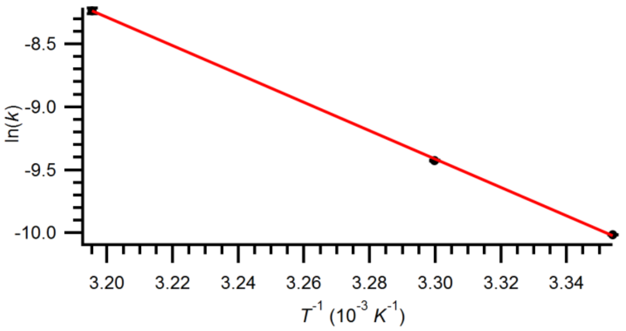

Supplementary Figure 58. Arrhenius plot. Arrhenius plot giving the values A = $\mathbf{9.960^{+4.824}_{-3.250}}$ x 10^{11} s^{-1} Ea = 93330 ± 998 J mol^{-1}**.**

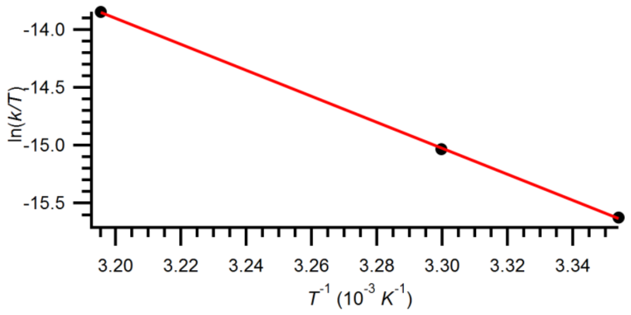

Supplementary Figure 59. Eyring plot. Eyring plot giving the values $\Delta H^{\ddagger}$ = 90.8 ± 0.989 kJ mol^{-1}, $\Delta S^{\ddagger}$ = -23.7 ± 3.23 J mol^{-1} K^{-1}.

Compound 11:

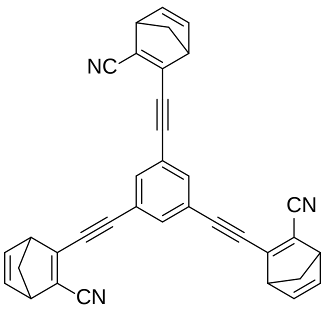

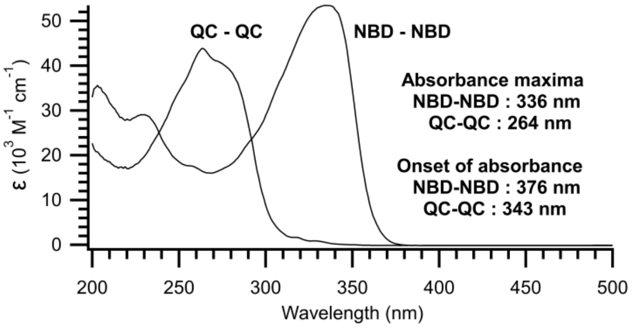

Supplementary Figure 60. UV-Vis spectra. UV-Vis spectra of **11** and $\mathbf{11}_{\mathbf{QC\text{-}QC\text{-}QC}}$.

Supplementary Note 3. Extinction coefficient for the NBD at absorbance maximum (336 nm) calculated as the average of three solutions (53703, 53430 and 52930) giving 53355 $M^{-1}cm^{-1}$.

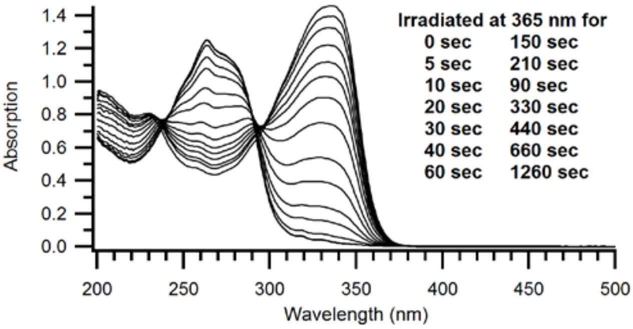

Supplementary Figure 61. UV-Vis spectra. UV-Vis spectra of **11** after irradiation at different irradiation times with 365 nm light. Concentration in the cuvette: 2.73 x 10-5 M.

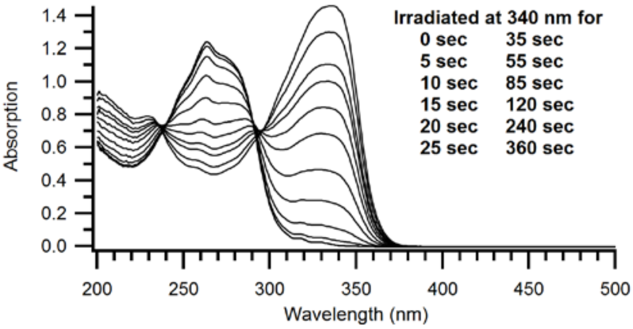

Supplementary Figure 62. UV-Vis spectra. UV-Vis spectra of **11** after irradiation at different irradiation times with 340 nm light. Concentration in the cuvette: 2.73 x 10^{-5} M.

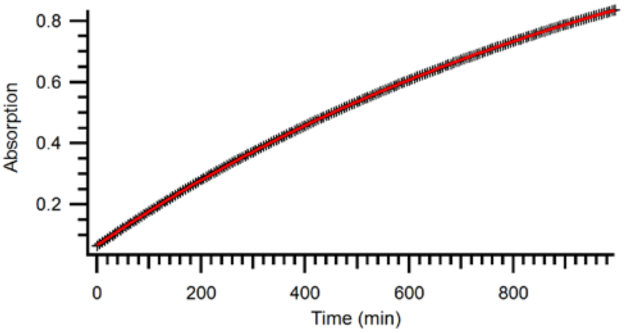

Supplementary Figure 63. Kinetics. Increase at absorbance maximum (336 nm) of **11** at 25 °C during the thermal backreaction.

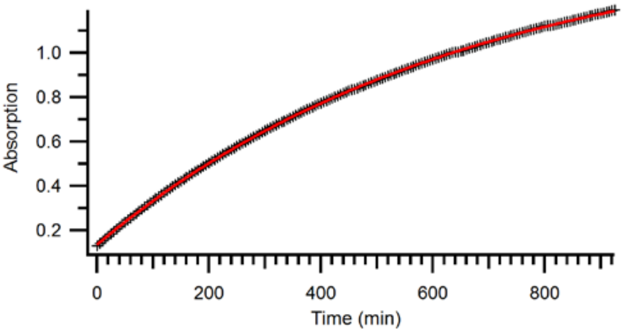

Supplementary Figure 64. Kinetics. Increase at absorbance maximum (336 nm) of **11** at 29.8 °C during the thermal backreaction.

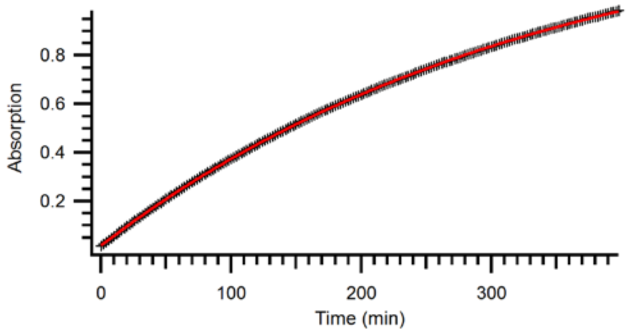

Supplementary Figure 65. Kinetics. Increase at absorbance maximum (336 nm) of **11** at 25 °C during the thermal backreaction.

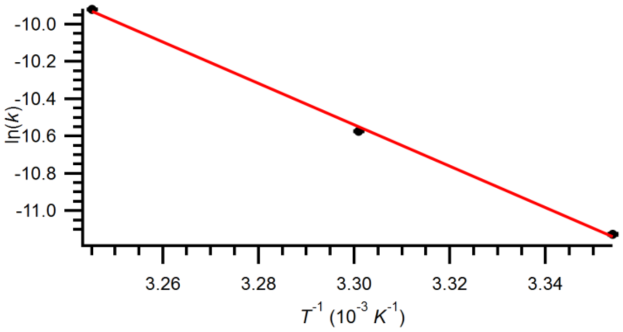

Supplementary Figure 66. Arrhenius plot. Arrhenius plot for **11** giving the values A = $\mathbf{2.12^{+5.44}_{-1.53}}$ x 10^{11} s^{-1} Ea = 92266 ± 3201 J mol^{-1}.

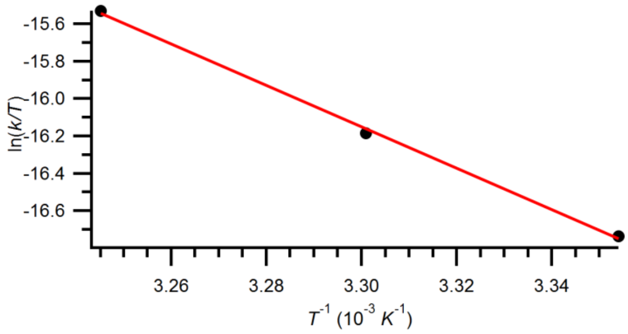

Supplementary Figure 67. Eyring plot. Eyring plot for **11** giving the values $\Delta H^{\ddagger}$ = 89.7 ± 3.18 kJ mol^{-1}, $\Delta S^{\ddagger}$ = -36.5 ± 10.6 J mol^{-1} K^{-1}

Compound 9:

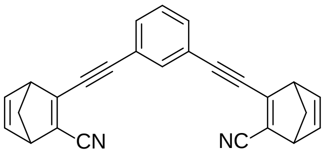

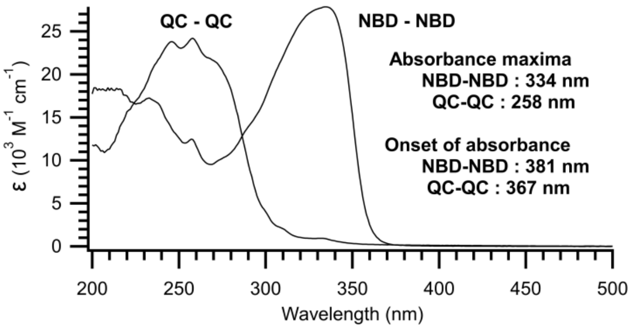

Supplementary Figure 68. UV-Vis spectra. UV-Vis spectra of **9** and **9**$_{\mathbf{QC\text{-}QC}}$.

Supplementary Note 4. Extinction coefficient for the NBD at absorbance maximum (334 nm) calculated as the average of three solutions (28389, 28161 and 27842) giving 28131 $M^{-1}cm^{-1}$.

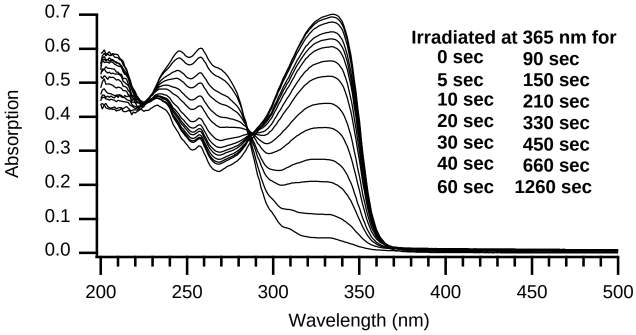

Supplementary Figure 69. UV-vis spectra. UV-Vis spectra of **9** after irradiation at different irradiation times with 365 nm light. Concentration in the cuvette: 2.49 x 10^{-5} M.

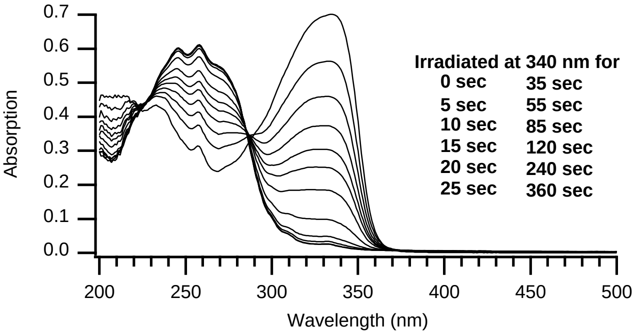

Supplementary Figure 70. UV-Vis spectra. UV-Vis spectra of **9** after irradiation at different irradiation times with 340 nm light. Concentration in the cuvette: 2.49 x 10^{-5} M.

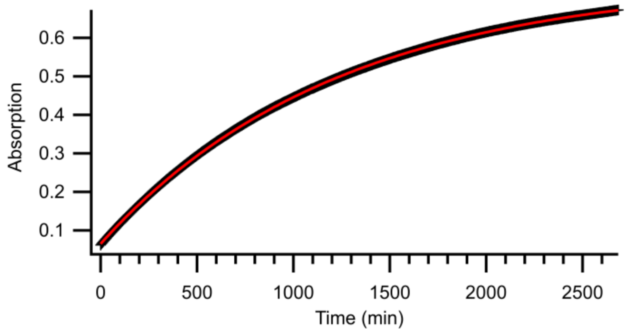

Supplementary Figure 71. Kinetics. Increase at absorbance maximum (334 nm) of **9** at 25 °C during the thermal backreaction.

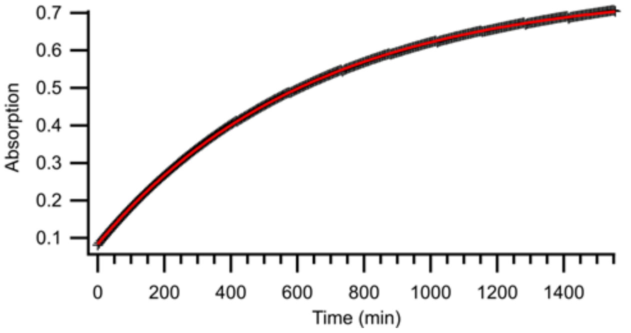

Supplementary Figure 72. Kinetics. Increase at absorbance maximum (334 nm) of **9** at 29.9 °C during the thermal backreaction.

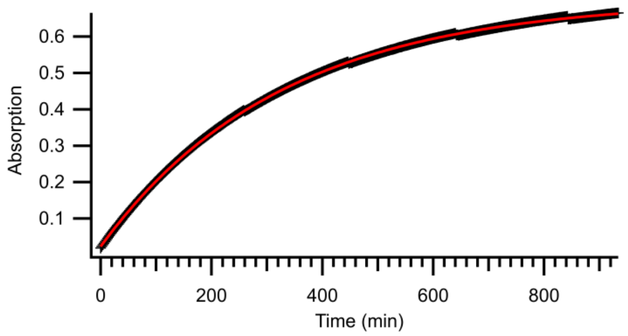

Supplementary Figure 73. Kinetics. Increase at absorbance maximum (334 nm) of **9** at 35 °C during the thermal backreaction.

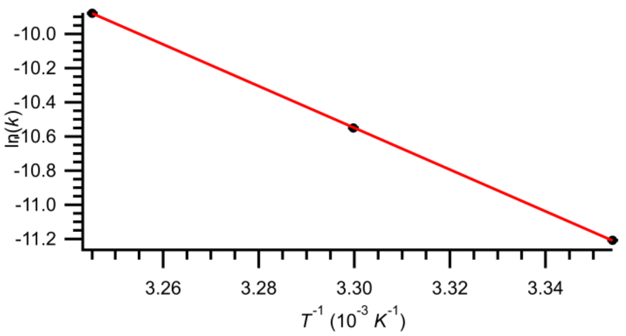

Supplementary Figure 74. Arrhenius plot. Arrhenius plot for **9** giving the values A = $\mathbf{8.42}^{+1.35}_{-1.17}$ x 10^{12} s^{-1} Ea = 101569 ± 375 J mol^{-1}.

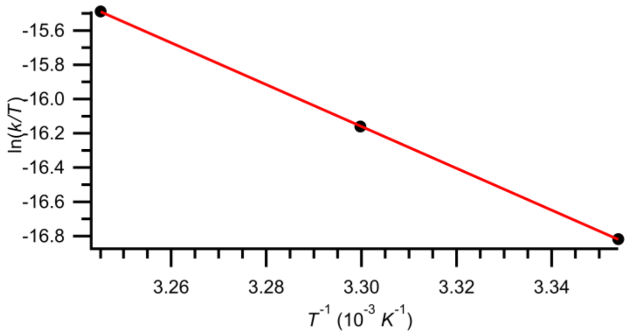

Supplementary Figure 75. Eyring plot. Eyring plot for **9** giving the values $\Delta H^{‡}$ = 99.0 ± 0.363 kJ mol^{-1}, $\Delta S^{‡}$ = -5.93 ± 1.20 J mol^{-1} K^{-1}.

Compound 14

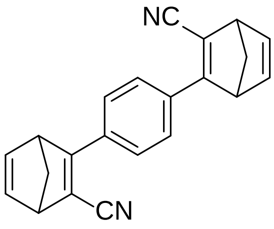

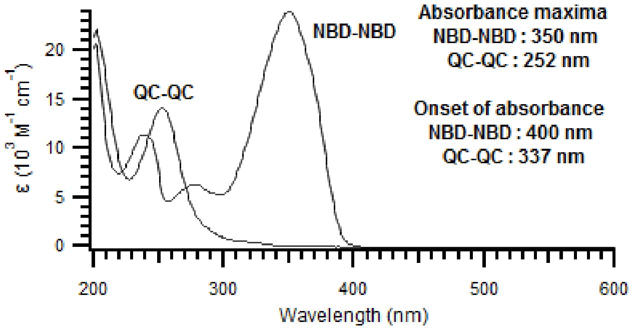

Supplementary Figure 76. UV-Vis spectra. UV-Vis spectra of **14** and **14**$_{\mathbf{QC\text{-}QC}}$.

Absorbance spectrum

Supplementary Note 5. Extinction coefficient for the NBD at absorbance maximum calculated as the average of three solutions (23366, 23367, 24000) gives 23578 $M^{-1}cm^{-1}$.

Irradiated at 340 nm

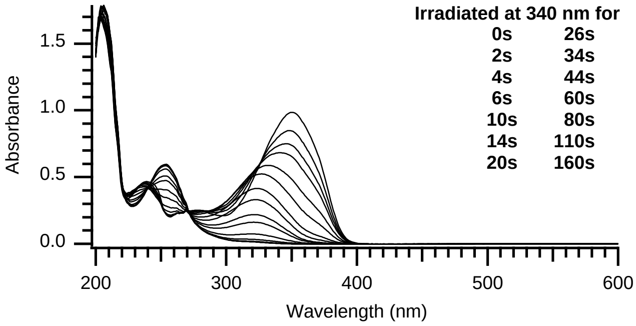

Supplementary Figure 77. UV-Vis spectra. UV-Vis spectra of **14** after irradiation at different irradiation times with 340 nm light. Concentration in the cuvette: 4.18 x 10^{-5} M.

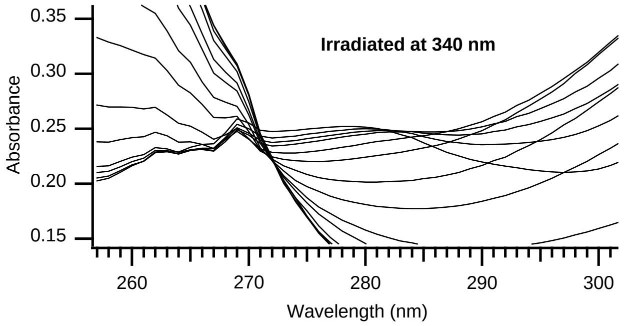

Supplementary Figure 78. UV-Vis spectra. Zoom of UV-Vis spectra of **14** after irradiation at different irradiation times with 340 nm light.

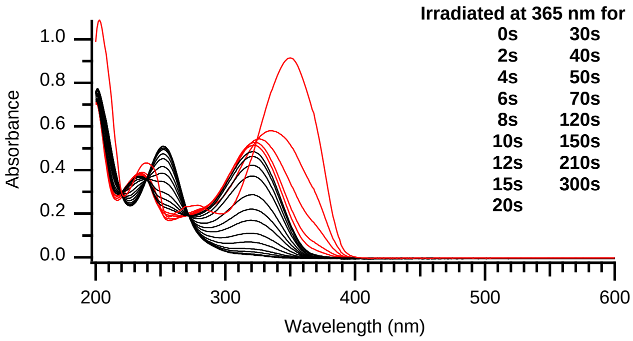

Supplementary Figure 79. UV-Vis spectra. UV-Vis spectra of **14** after irradiation at different irradiation times with 365 nm light. Concentration in the cuvette: 3.88 x 10^{-5} M.

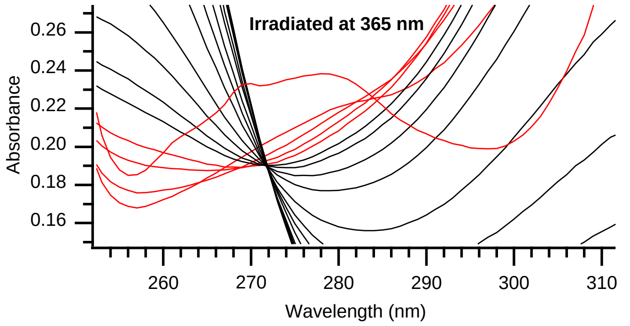

Supplementary Figure 80. UV-Vis spectra. Zoom of UV-Vis spectra of **14** after irradiation at different irradiation times with 365 nm light.

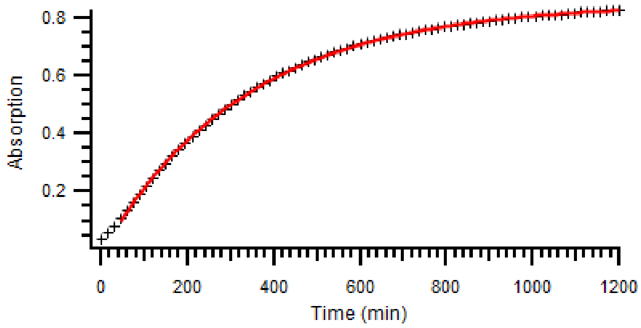

Supplementary Figure 81. Kinetics. Increase at absorbance maximum (350 nm) of **14** at 57.1 °C during the thermal backreaction.

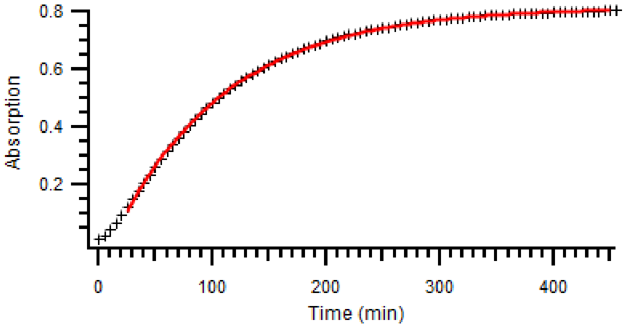

Supplementary Figure 82. Kinetics. Increase at absorbance maximum (350 nm) of **14** at 67.8 °C during the thermal backreaction.

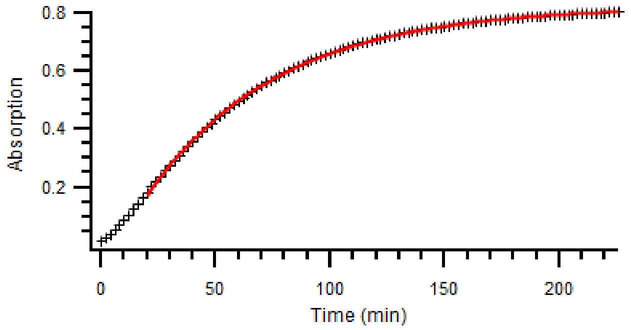

Supplementary Figure 83. Kinetics. Increase at absorbance maximum (350 nm) of **14** at 72.7 °C during the thermal backreaction.

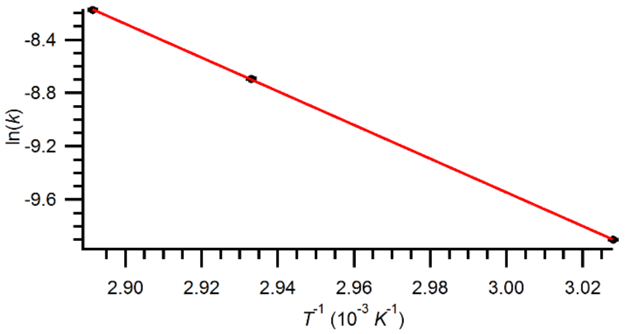

Supplementary Figure 84. Arrhenius plot. Arrhenius plot for **14** giving the values A = $3.55^{+0.257}_{-0.240}$ x 10^{12} s^{-1}, Ea = 106533 ± 197 J mol^{-1}.

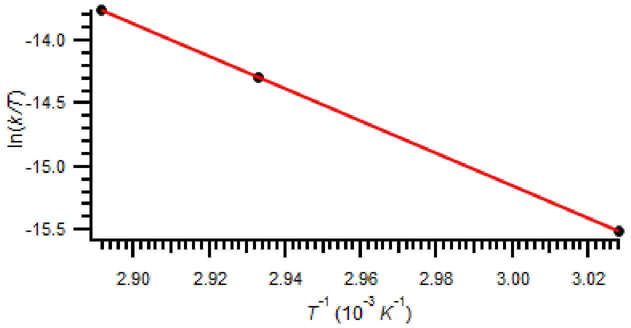

Supplementary Figure 85. Eyring plot. Eyring plot giving the values $\Delta H^{‡}$ = 104 ± 0.182 kJ mol^{-1} $\Delta S^{‡}$ = -3.92 ± 0.536 J mol^{-1} K^{-1}.

Compound 14_{QC-NBD}

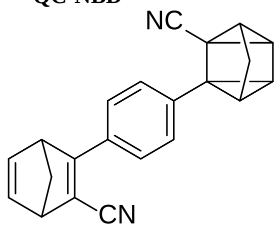

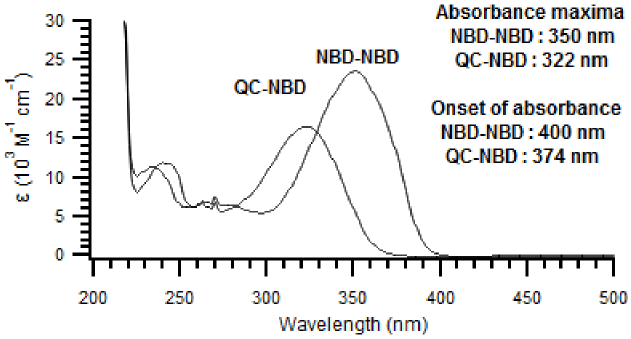

Supplementary Figure 86. UV-Vis spectra. UV-Vis spectra of **14** and **14_{QC-NBD}**.

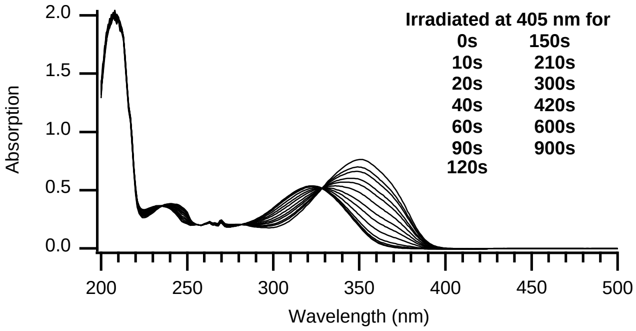

Supplementary Figure 87. UV-Vis spectra. UV-Vis spectra of **14_{QC-NBD}** after irradiation at different irradiation times with 340 nm light. Concentration in the cuvette: 3.24 x 10^{-5} M.

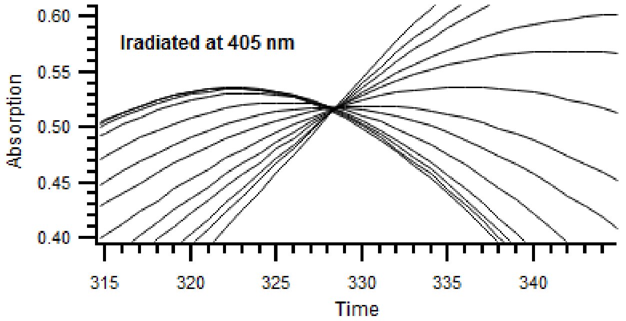

Supplementary Figure 88. UV-Vis spectra. Zoom of UV-Vis spectra of **14_{QC-NBD}** after irradiation at different irradiation times with 340 nm light. Concentration in the cuvette: 3.24 x 10^{-5} M.

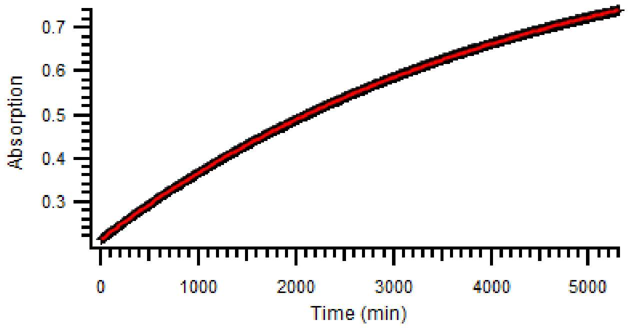

Supplementary Figure 89. Kinetics. Increase at absorbance maximum (350 nm) of **14** at 25.0 °C during the thermal backreaction.

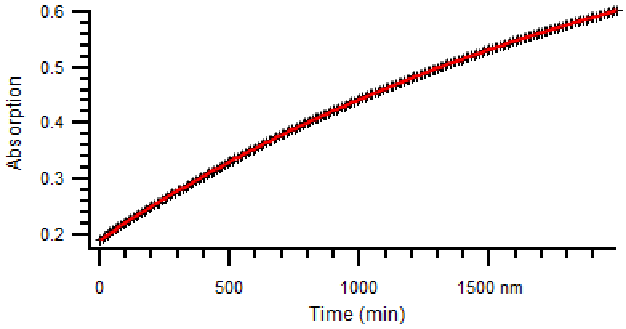

Supplementary Figure 90. Kinetics. Increase at absorbance maximum (350 nm) of **14** at 29.9 °C during the thermal backreaction.

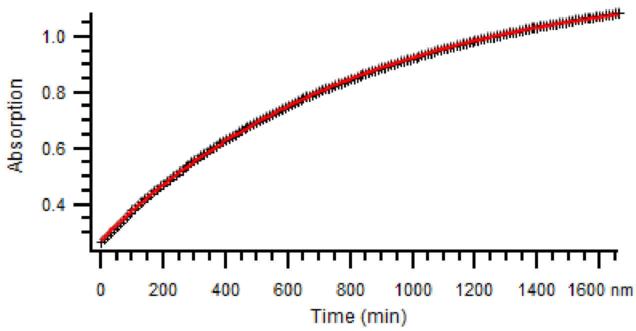

Supplementary Figure 91. Kinetics. Increase at absorbance maximum (350 nm) of **14** at 39.8 °C during the thermal backreaction.

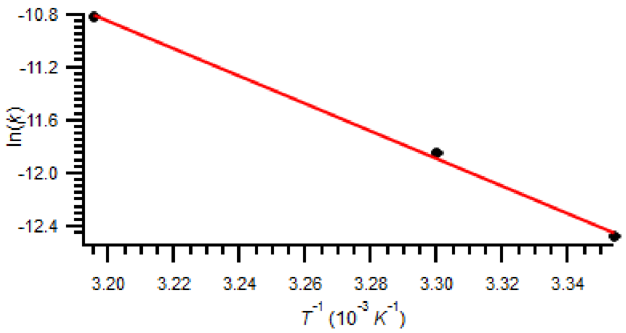

Supplementary Figure 92. Arrhenius plot. Arrhenius plot giving the values A = $\mathbf{4.42^{+14.1}_{-3.36}}$ x 10^9 s^{-1}, Ea = 85913 ± 3633 J mol^{-1}.

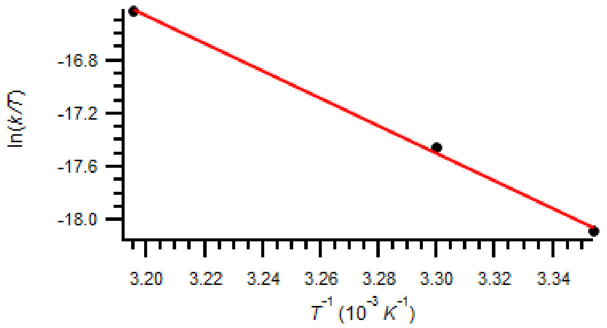

Supplementary Figure 93. Eyring plot. Eyring plot giving the values $\Delta H^{\ddagger}$ = 83.4 ± 3.64 kJ mol^{-1} $\Delta S^{\ddagger}$ = -68.8 ± 12.0 J mol^{-1} K^{-1}.

Compound 15

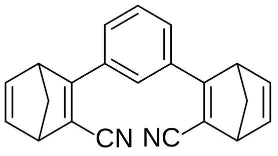

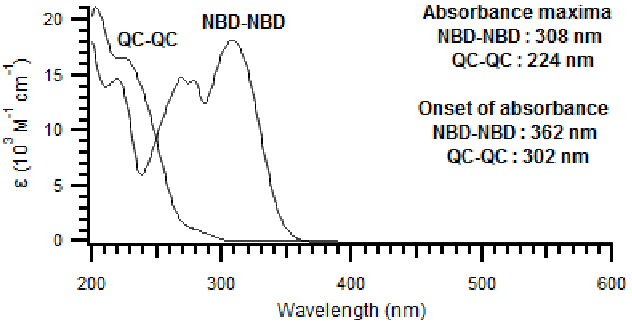

Supplementary Figure 94. UV-Vis spectra. UV-Vis spectra of **15** and $\mathbf{15_{QC\text{-}QC}}$

Supplementary Note 6. Extinction coefficient for the NBD calculated as the average of three solutions (18316, 18189, 18443) gives 18316 $M^{-1}cm^{-1}$.

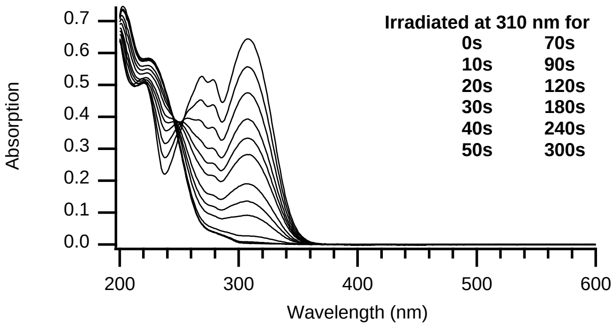

Supplementary Figure 95. UV-Vis spectra. UV-Vis spectra of **15** after irradiation at different irradiation times with 310 nm light. Concentration in the cuvette: 3.52 x 10^{-5} M.

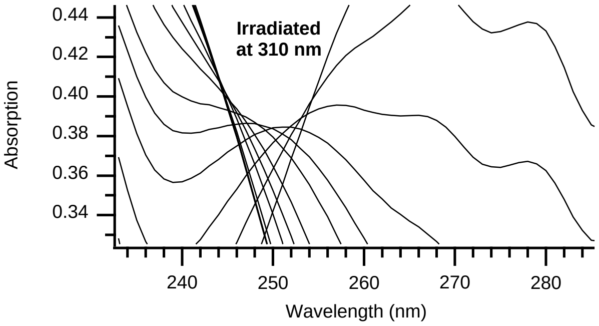

Supplementary Figure 96. UV-Vis spectra. Zoom of UV-Vis spectra of **15** after irradiation at different irradiation times with 310 nm light. Concentration in the cuvette: 3.52 x 10^{-5} M.

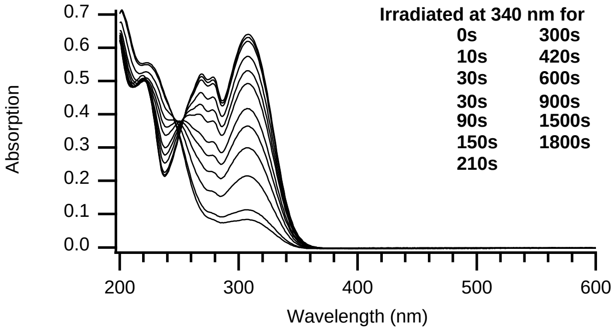

Supplementary Figure 97. UV-Vis spectra. UV-Vis spectra of **15** after irradiation at different irradiation times with 340 nm light. Concentration in the cuvette: 3.49 x 10^{-5} M.

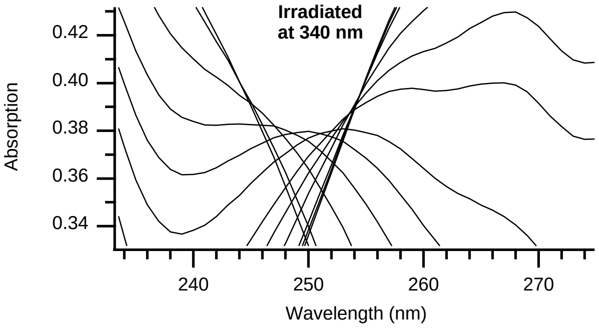

Supplementary Figure 98. UV-Vis spectra. Zoom of UV-Vis spectra of **15** after irradiation at different irradiation times with 340 nm light. Concentration in the cuvette: 3.49 x 10^{-5} M.

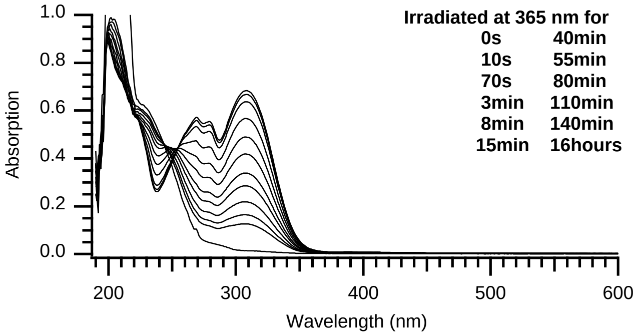

Supplementary Figure 99. UV-Vis spectra. UV-Vis spectra of **15** after irradiation at different irradiation times with 365 nm light. Concentration in the cuvette: 3.64 x 10^{-5} M.

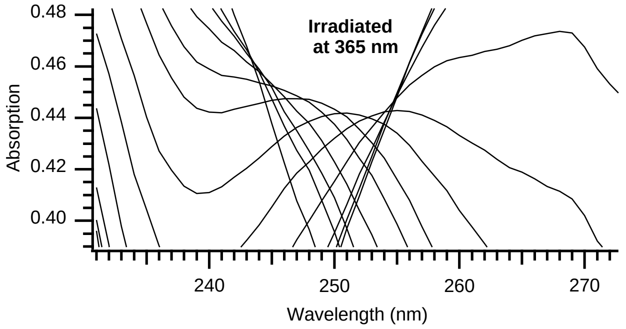

Supplementary Figure 100. UV-Vis spectra. UV-Vis spectra of **15** after irradiation at different irradiation times with 365 nm light.

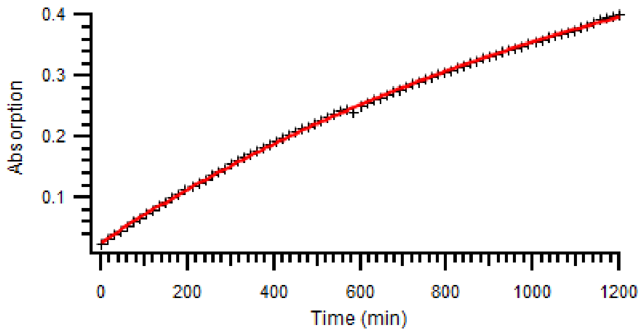

Supplementary Figure 101. Kinetics. Increase at absorbance maximum (308 nm) of **15** at 57.1 °C during the thermal backreaction.

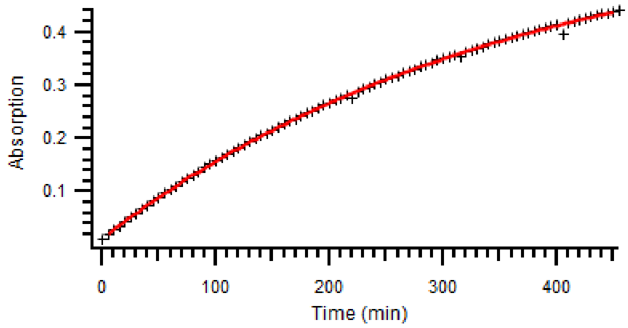

Supplementary Figure 102. Kinetics. Increase at absorbance maximum (308 nm) of **15** at 67.8 °C during the thermal backreaction.

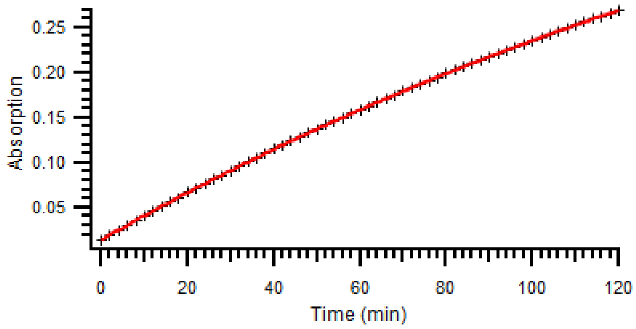

Supplementary Figure 103. Kinetics. Increase at absorbance maximum (308 nm) of **15** at 72.7 °C during the thermal backreaction.

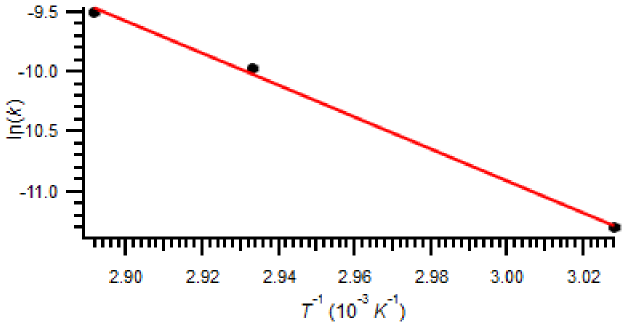

Supplementary Figure 104. Arrhenius plot. Arrhenius plot for **15** giving the values A = $\mathbf{3.29^{+17.0}_{-2.75}}$ x 10^{12} s^{-1}, Ea = 110100 ± 5122 J mol^{-1}.

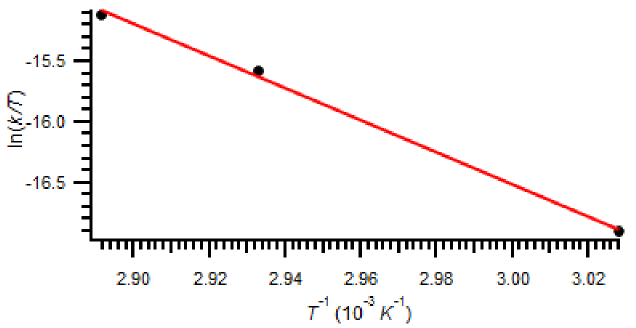

Supplementary Figure 105. Eyring plot. Eyring plot giving the values $\Delta H^{‡}$= 107 ± 15.1 kJ mol^{-1} $\Delta S^{‡}$ = -14. 6 ± 15.13 J mol^{-1} K^{-1}.

Heat release (DSC)

Compound 8

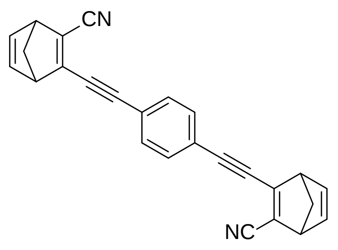

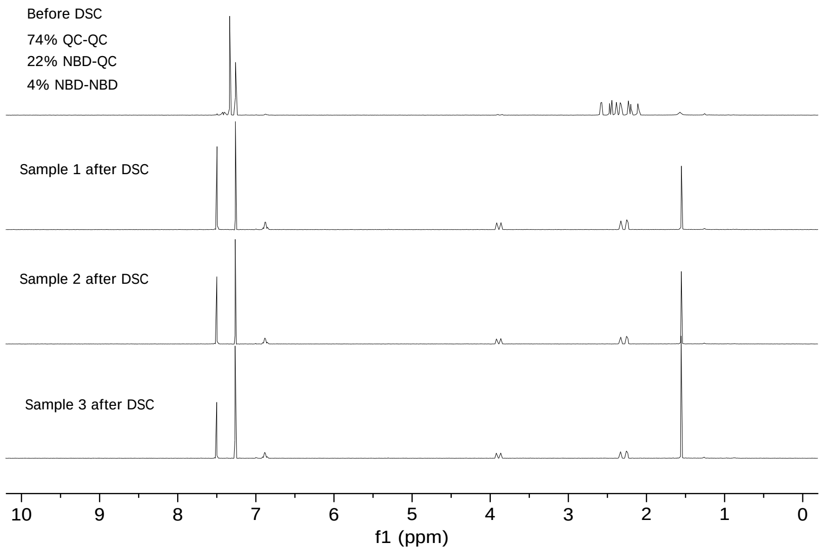

Supplementary Figure 106. NMR spectra. NMR spectra of $\mathbf{8_{QC\text{-}QC}}$ before DSC and of samples 1-3 after heat release.

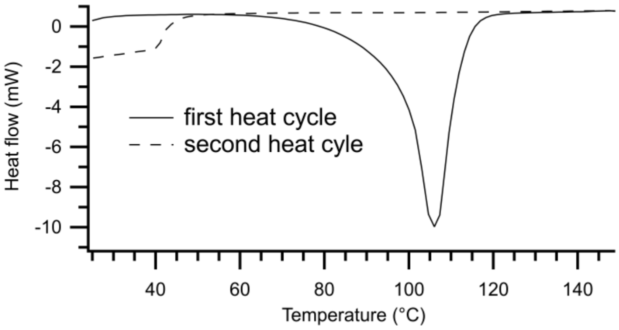

Supplementary Figure 107. DSC spectrum. DSC spectrum of the heat release for $\mathbf{8_{QC\text{-}QC}}$. Heating rate 20 °C min^{-1}. Amount of sample: 1.50 mg. Integration area: 50.18–144.49 °C. Heat release: 439.94 kJ kg^{-1}. Corrected heat release: 519.35 kJ kg^{-1}

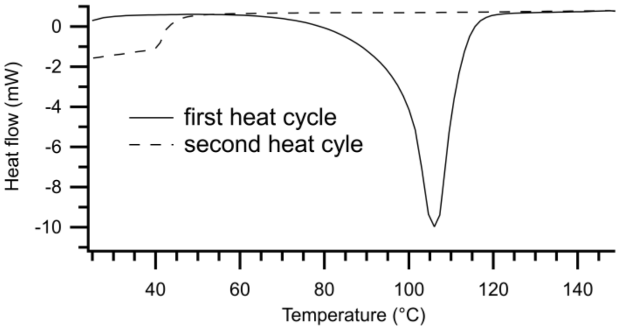

Supplementary Figure 108. DSC spectrum. DSC spectrum of the heat release for $\mathbf{8_{QC\text{-}QC}}$. Heating rate 20 °C min^{-1}. Amount 1.40 mg. Integration area: 54.89–127.56 °C. Heat release: 427.01 kJ kg^{-1}. Corrected heat release: 504.08 kJ kg^{-1}.

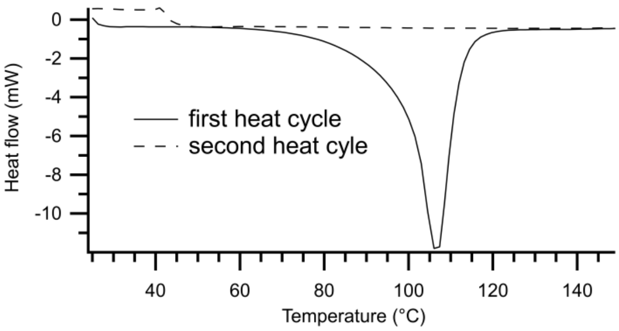

Supplementary Figure 109. DSC spectrum. DSC spectrum of the heat release for $\mathbf{8_{QC\text{-}QC}}$. Heating rate 20 °C min^{-1}. Amount 1.27 mg. Integration area: 51.75–137.59 °C. Heat release: 439.77 kJ kg^{-1}. Corrected heat release: 519.15 kJ mol^{-1}.

Compound 9

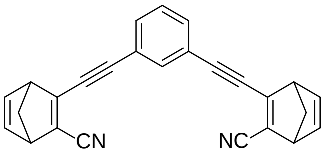

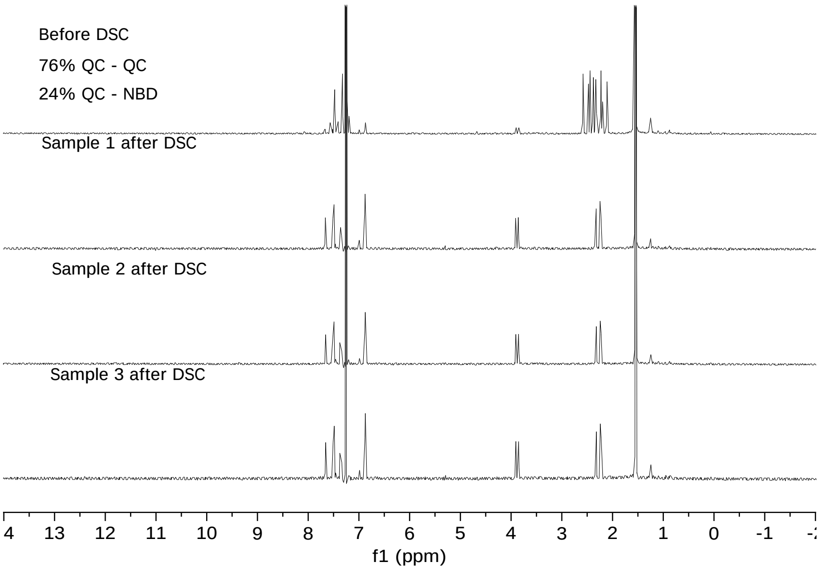

Supplementary Figure 110. NMR spectra. NMR spectra of $\mathbf{9}_{\mathbf{QC\text{-}QC}}$ before DSC and of samples 1-3 after heat release.

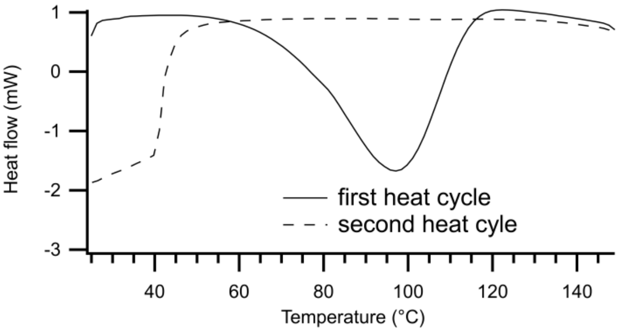

Supplementary Figure 111. DSC spectrum. DSC spectrum of the heat release for $\mathbf{9}_{\mathbf{QC\text{-}QC}}$. Heating rate 20 °C min^{-1}. Amount 1.03 mg. Integration area: 46.33–124.72 °C. Heat release: 230.11 kJ kg^{-1}. Corrected heat release: 263.13 kJ kg^{-1}.

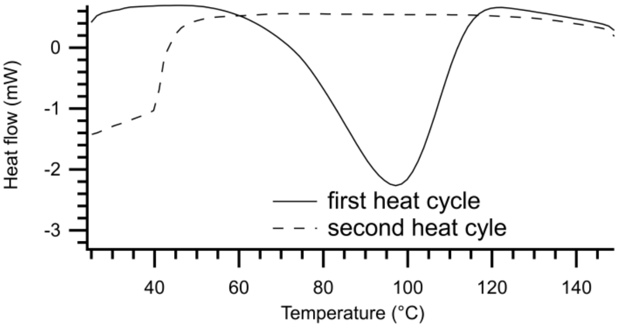

Supplementary Figure 112. DSC spectrum. DSC spectrum of the heat release for $\mathbf{9}_{\mathbf{QC\text{-}QC}}$. Heating rate 20 °C min^{-1}. Amount 1.16 mg. Integration area: 47.34–121.88 °C. Heat release: 251.94 kJ kg^{-1}. Corrected heat release: 288.10 kJ kg^{-1}.

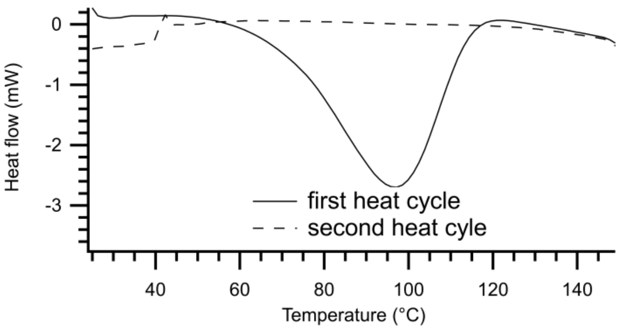

Supplementary Figure 113. DSC spectrum. DSC spectrum of the heat release for $\mathbf{9_{QC\text{-}QC}}$. Heating rate 20 °C min^{-1}. Amount 1.07 mg. Integration area: 43.22–122.91 °C. Heat release: 244.24 kJ kg^{-1}. Corrected heat release: 279.29 kJ kg^{-1}.

Compound 15

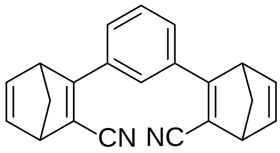

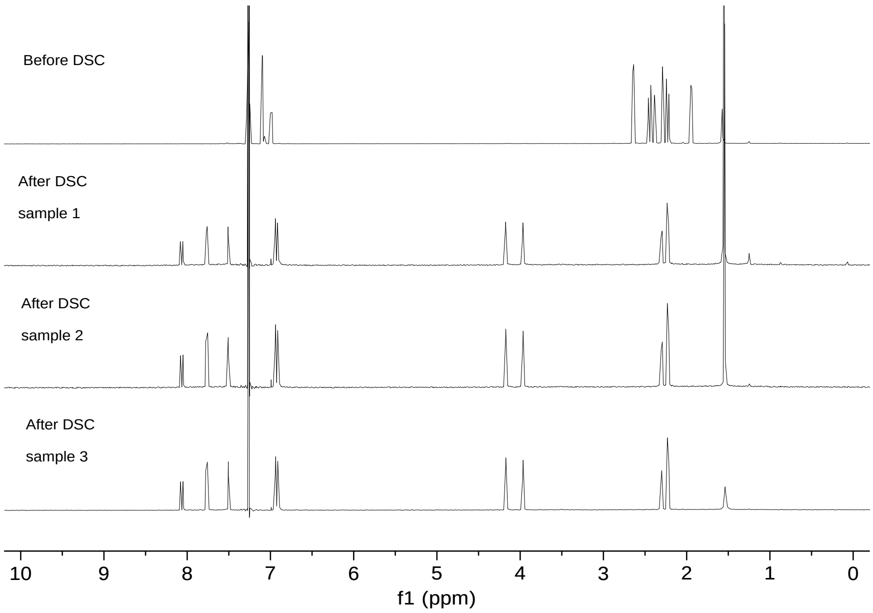

Supplementary Figure 114. NMR spectra. NMR spectra of $\mathbf{15_{QC\text{-}QC}}$ before DSC and of samples 1-3 after heat release.

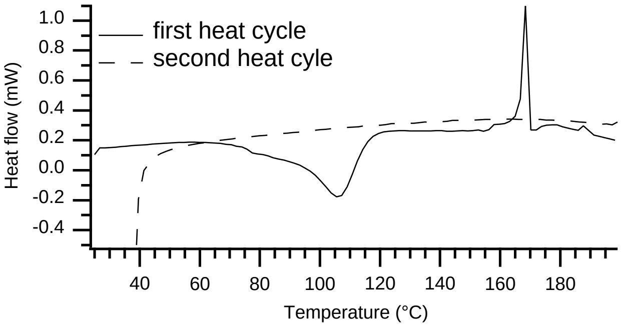

Supplementary Figure 115. DSC spectrum. DSC spectrum of the heat release for $\mathbf{15}_{\mathbf{QC\text{-}QC}}$. Heating rate 1 °C min^{-1}. Amount 1.60 mg. Integration area: 49.99–128.21 °C. Heat release: 559.52 kJ kg^{-1}.

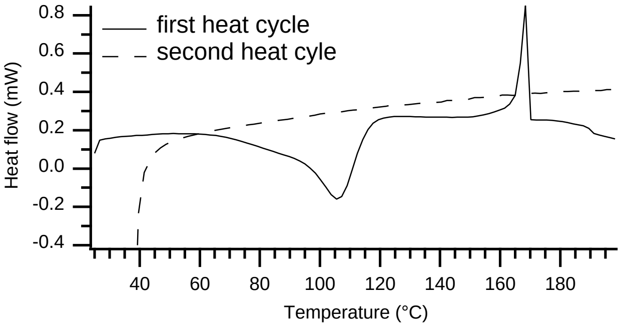

Supplementary Figure 116. DSC spectrum. DSC spectrum of the heat release for $15_{QC\text{-}QC}$. Heating rate 1 °C min^{-1}. Amount 1.27 mg. Integration area: 49.59–125.80 °C. Heat release: 563.11 kJ kg^{-1}.

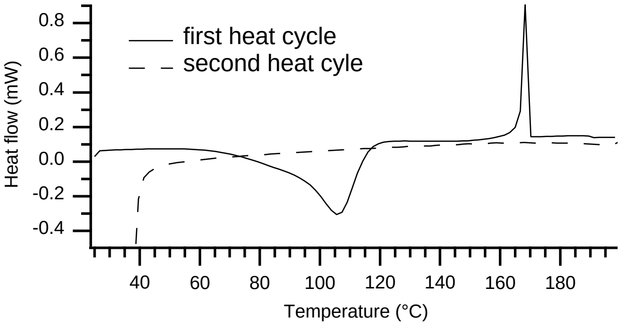

Supplementary Figure 117. DSC spectrum. DSC spectrum of the heat release for $\mathbf{15}_{\mathbf{QC\text{-}QC}}$. Heating rate 1 °C min^{-1}. Amount 2.74 mg. Integration area: 50.80–125.60 °C. Heat release: 555.31 kJ kg^{-1}.

NMR studies of photo conversion

Compound 5:

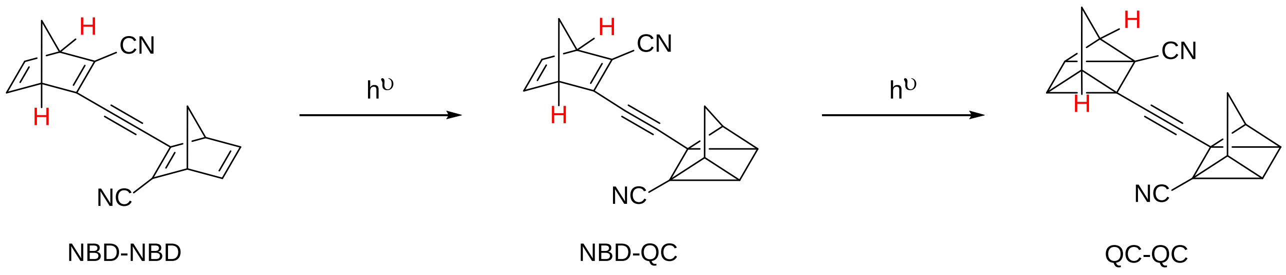

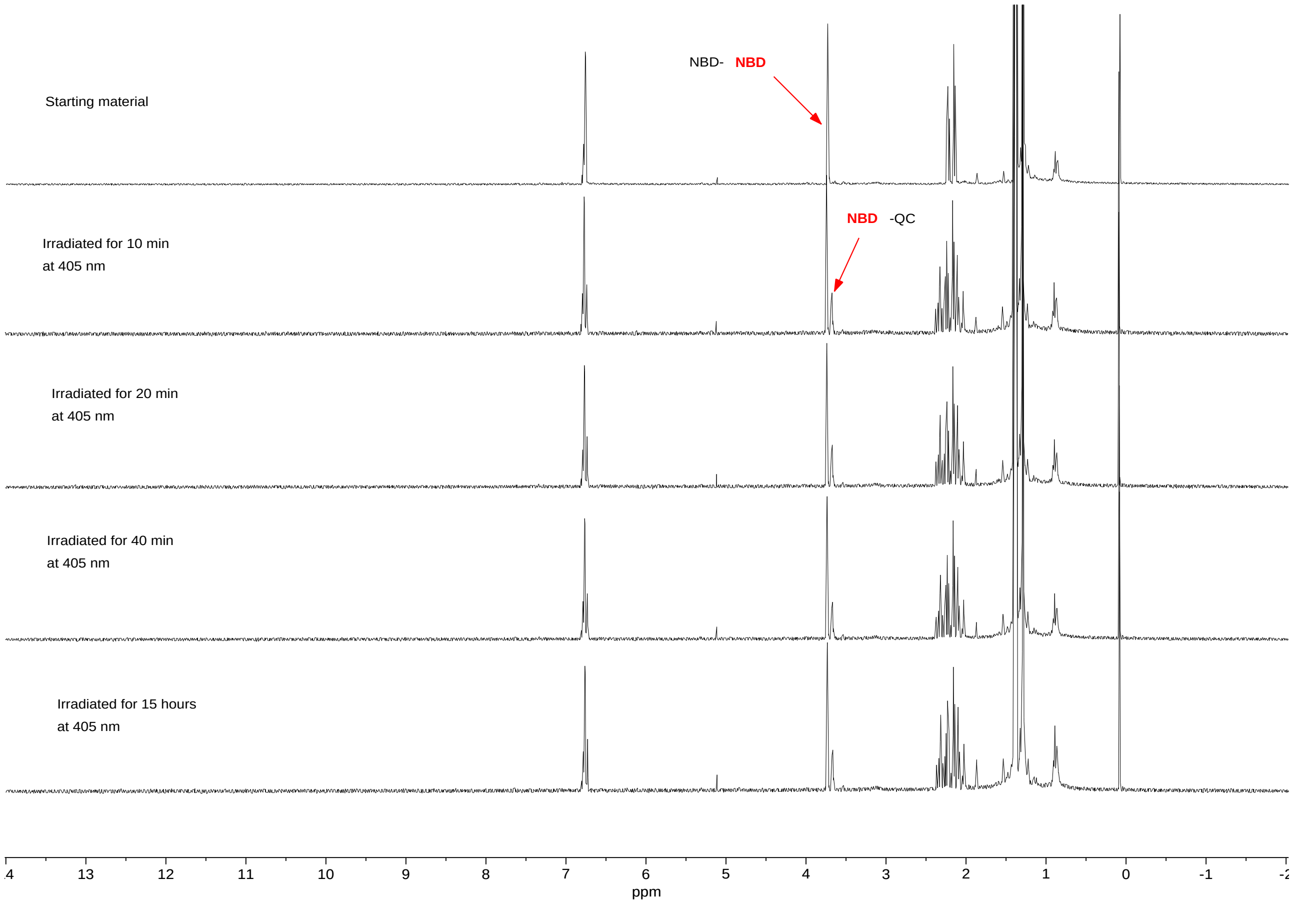

Supplementary Figure 118. NMR spectra. ^{1}H NMR spectra of **5** cyclohexane-*d12* after varying irradiation times with a 405 nm diode.

Compound 9:

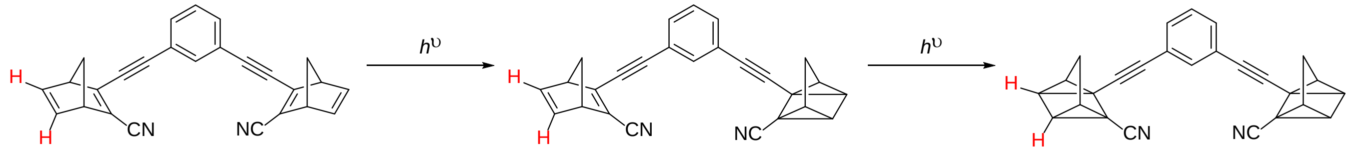

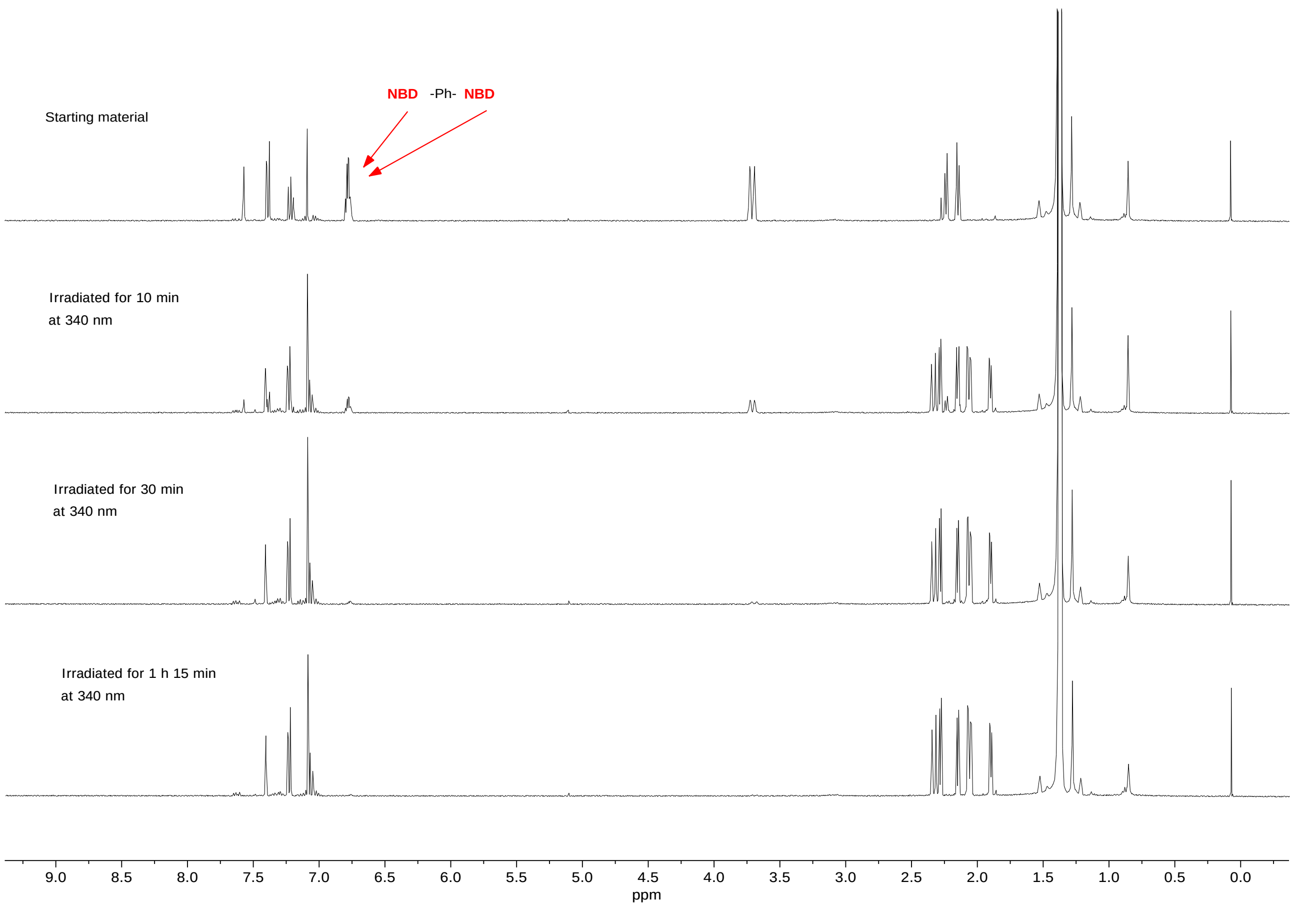

Supplementary Figure 119. NMR spectra. ^{1}H NMR spectra in cyclohexane-*d12* of **9** after varying irradiation times with a 340 nm diode.

Compound 8:

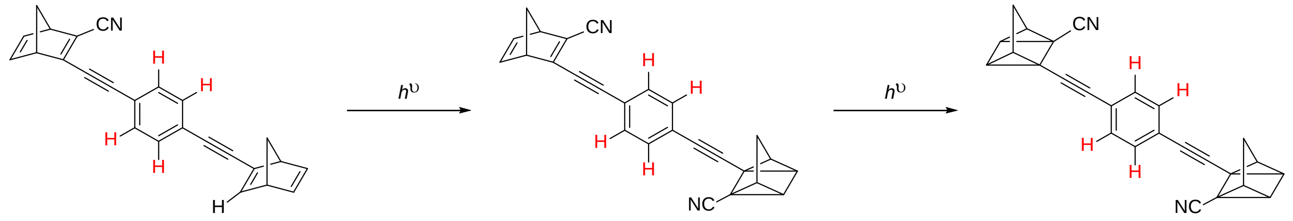

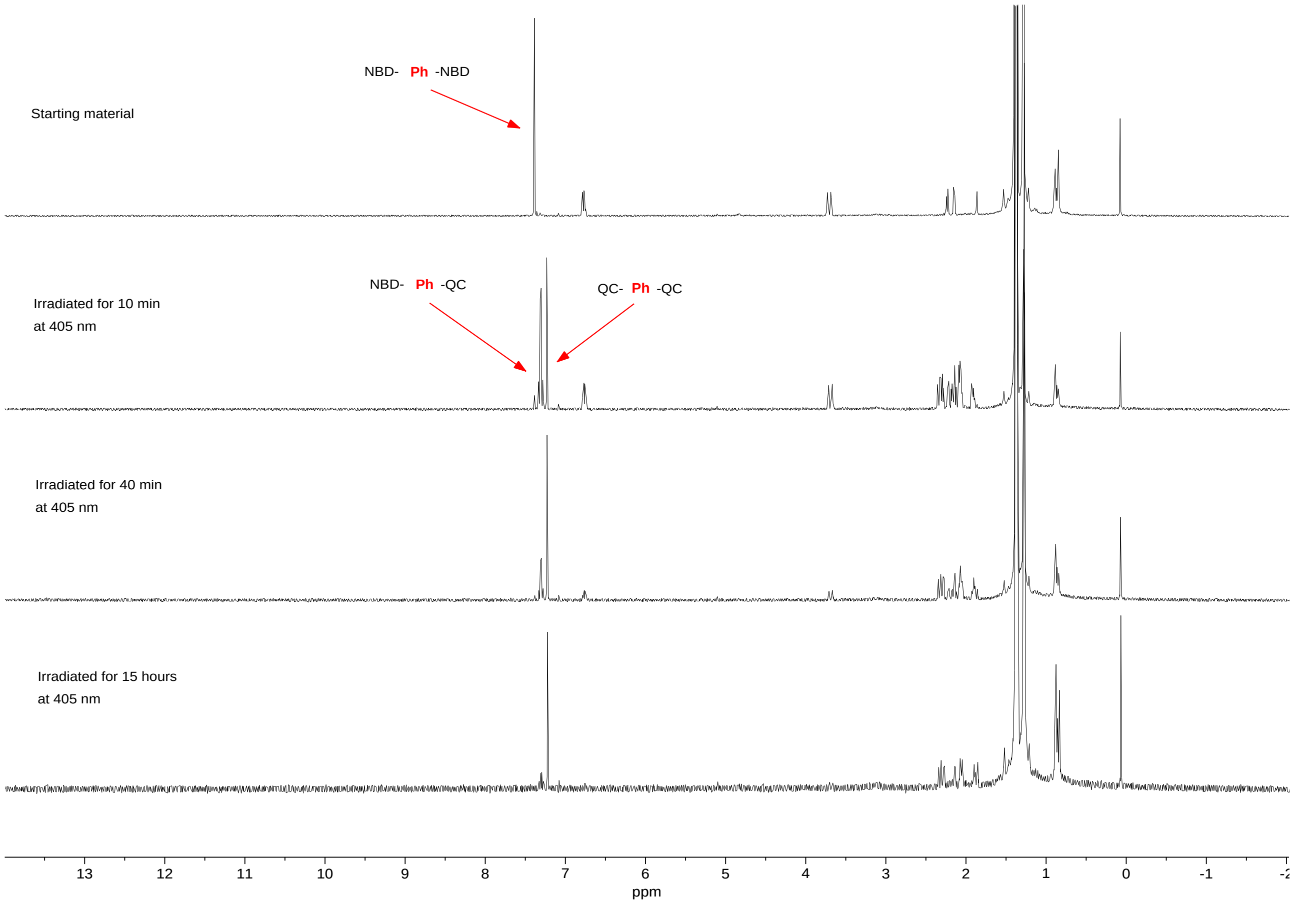

Supplementary Figure 120. NMR spectra. ^{1}H NMR spectra in cyclohexane-*d12* of **8** after varying irradiation times with a 405 nm diode.

Compound 14

Irradiation

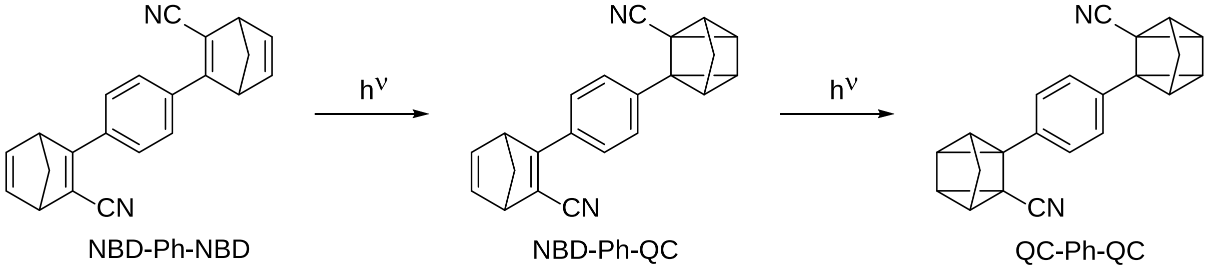

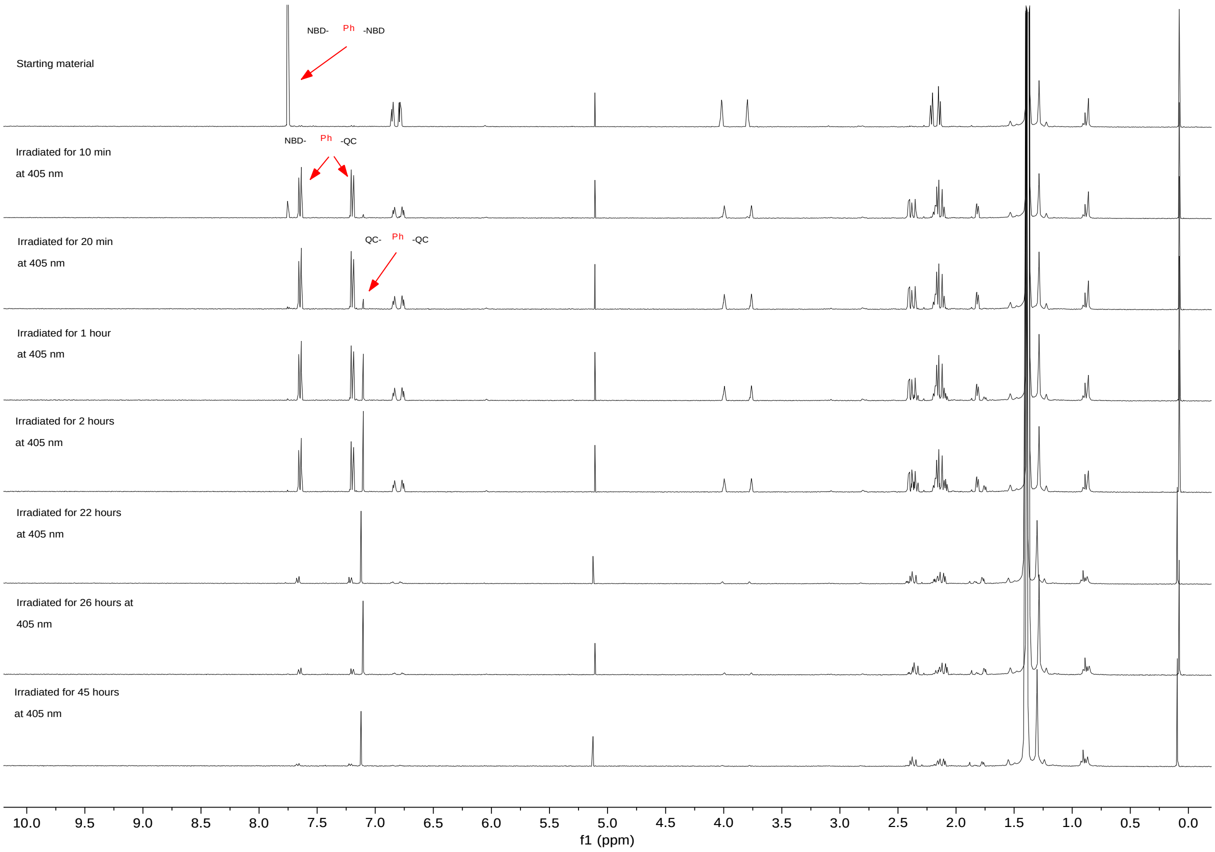

Supplementary Figure 121. NMR spectra. ^{1}H NMR spectra in cyclohexane-*d12* of **14** after varying irradiation times with a 405 nm diode.

Back conversion

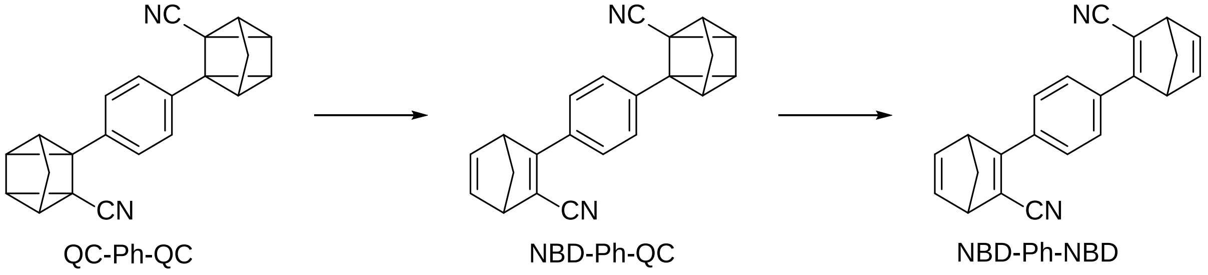

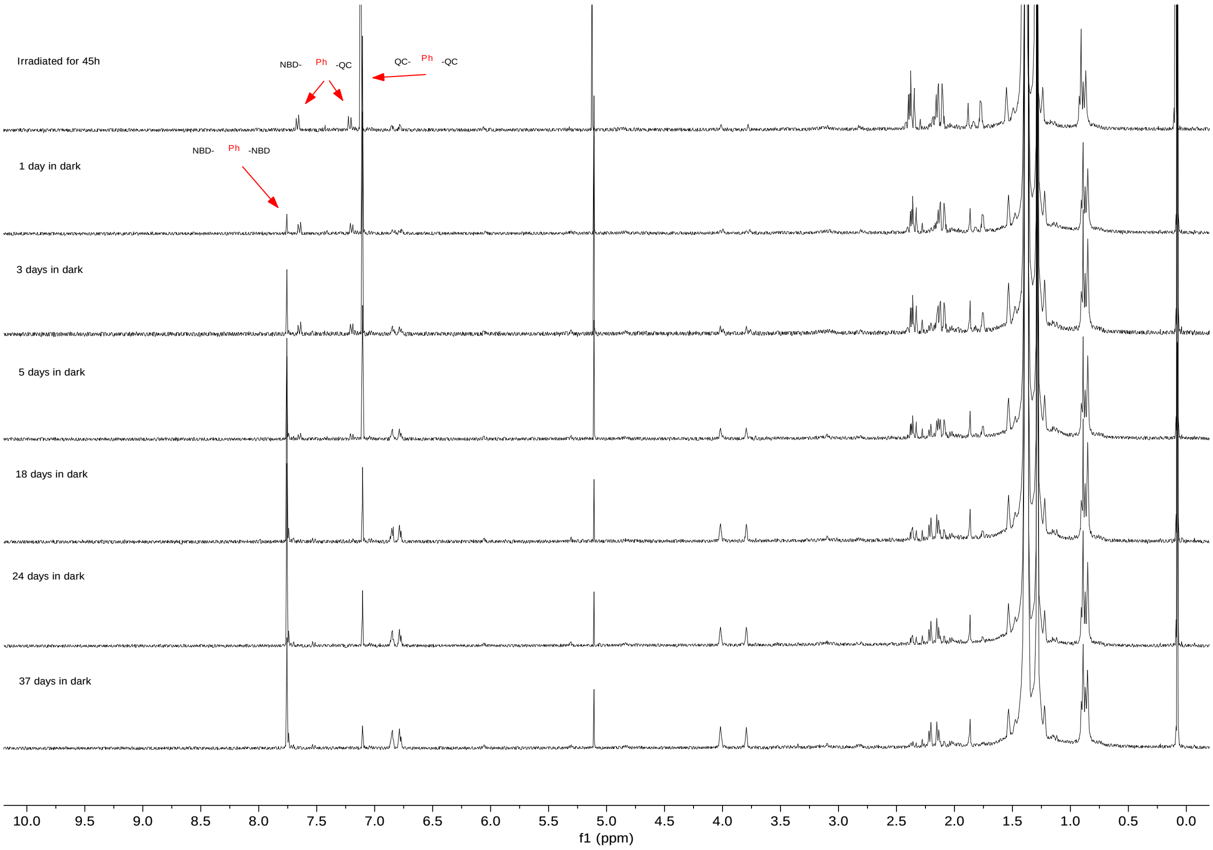

Supplementary Figure 122. NMR spectra. ^{1}H NMR spectra in cyclohexane-*d12* following the backreaction from $\mathbf{14_{QC\text{-}QC}}$ to **14** at room temperature.

Compound 15

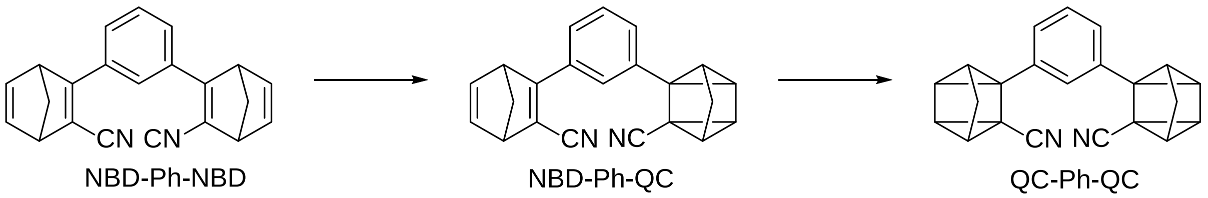

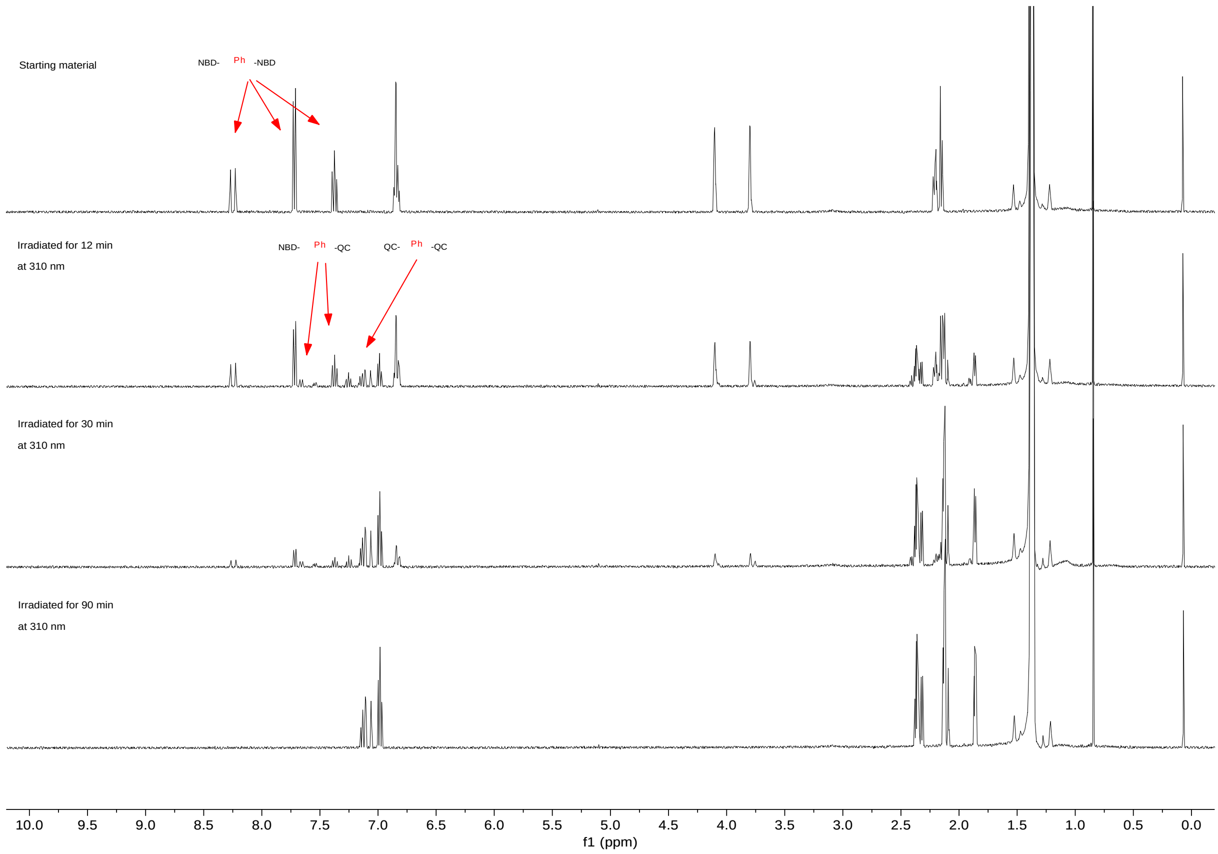

Supplementary Figure 123. NMR spectra. ^{1}H NMR spectra in cyclohexane-*d12* of **15** after varying irradiation times with a 310 nm diode.

Photoisomerisation Quantum Yields

Photon flux

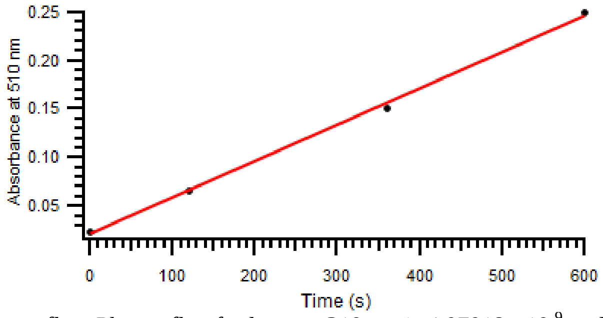

Supplementary Figure 124. Photon flux. Photon flux for lamp at 310 nm is 4.07018 · 10^{-9} mol s^{-1}. Used for **15**.

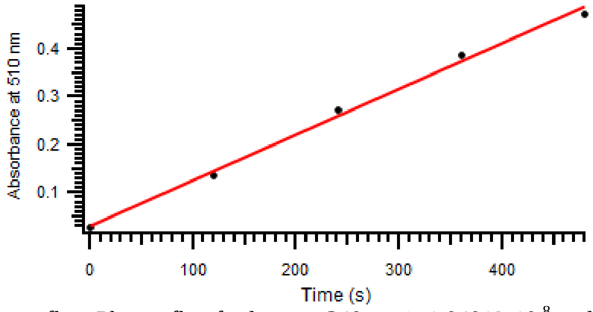

Supplementary Figure 125. Photon flux. Photon flux for lamp at 340 nm is 1.04012·10^{-8} mol s^{-1}. Used for **15**.

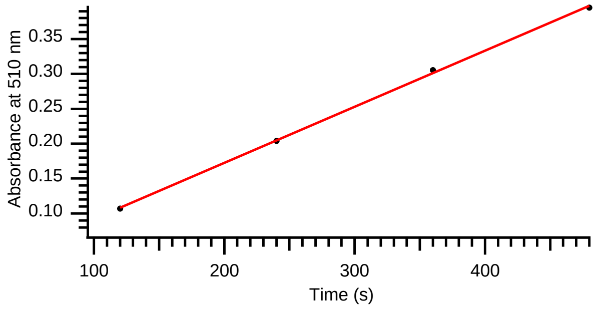

Supplementary Figure 126. Photon flux. Photon flux for lamp at 340 nm is 8.76646·10^{-9} mol s^{-1}. Used for **14**, **5**, **8**, **9** and **11**.

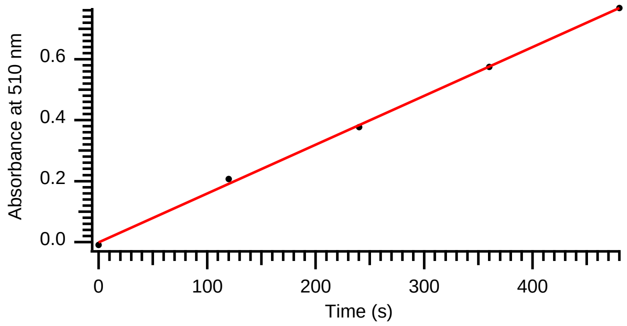

Supplementary Figure 127. Photon flux. Photon flux for lamp at 365 nm is 1.78881·10^{-8} mol s^{-1}. Used for **14**, **5** and **8**.

Compound 14

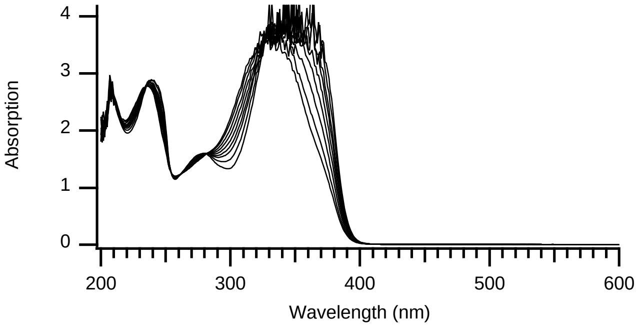

Supplementary Figure 128. UV-Vis spectra. UV-Vis spectra of **14,** sample 1, after varying irradiation times with a 365 nm diode. The concentration of NBD-NBD determined at 385 nm, where the concentration of the QC-NBD is negligible.

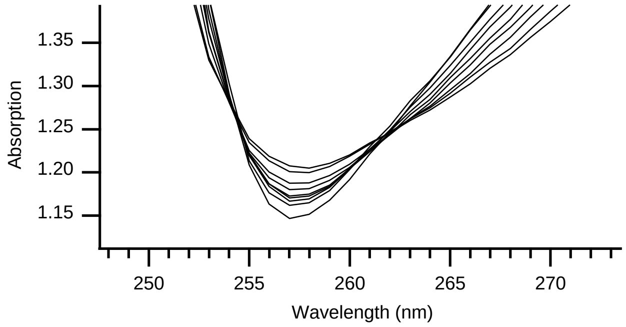

Supplementary Figure 129. UV-Vis spectra. Zoom of the UV-Vis spectra of **14,** sample 1, after varying irradiation times.

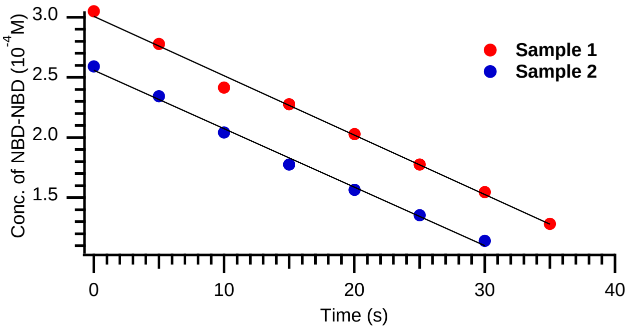

Supplementary Figure 130. Quantum yield plot. Plot of the quantum yield measurements for compound **14** at 365 nm.Volume of sample 1: 2.618 mL. Volume of sample 2: 2.701 mL. $\Phi_{\text{sample 1}}$ = 72%. $\Phi_{\text{sample 2}}$ = 73%.

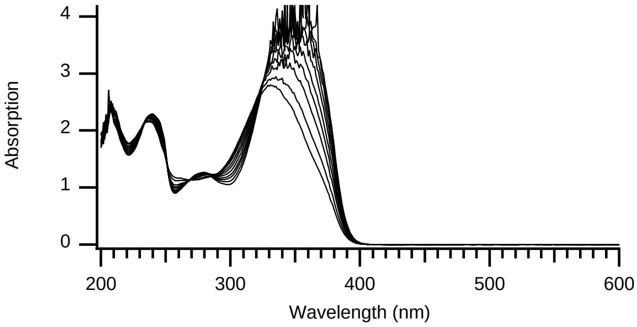

Supplementary Figure 131. UV-Vis spectra. UV-Vis spectra of **14,** sample 1, after varying irradiation times with a 340 nm diode. The concentration of NBD-NBD determined at 385 nm, where the concentration of the QC-NBD is negligible.

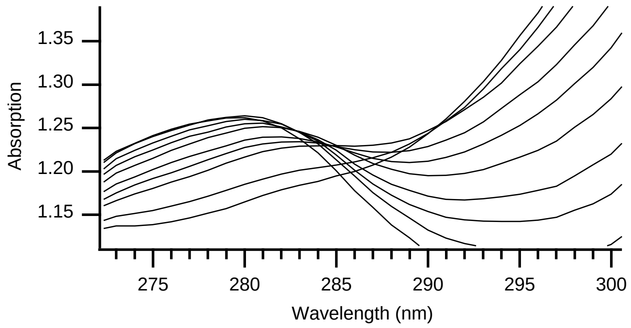

Supplementary Figure 132. UV-Vis spectra. Zoom of the UV-Vis spectra of **14,** sample 1, after varying irradiation times with a 340 nm diode.

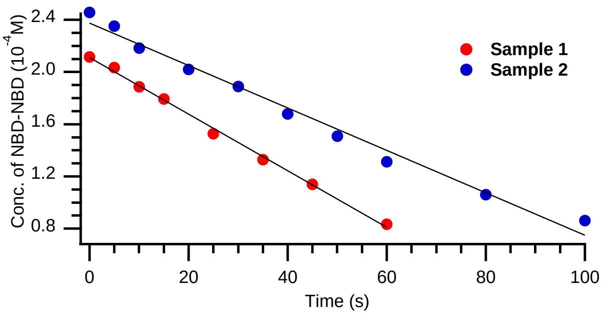

Supplementary Figure 133. Quantum yield plot. Plot of the quantum yield measurements for compound **14** at 340 nm.Volume of sample 1: 2.608 mL. Volume of sample 2: 3.010 mL. $\Phi_{sample\ 1}$ = 65%. $\Phi_{sample\ 2}$ = 56%.

Compound $14_{QC\text{-}NBD}$

Supplementary Note 7. The Quantum yield was measured by irradiating for 40 min at 405 nm, there by converting the **NBD-NBD** to the **NBD-QC** before the measurement at 340 nm was performed.

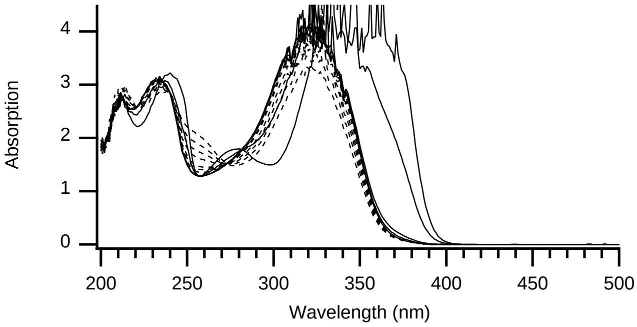

Supplementary Figure 134. UV-Vis spectra. UV-Vis spectra of $\mathbf{14}_{QC\text{-}NBD}$, sample 1, after varying irradiation times with a 365 nm diode.

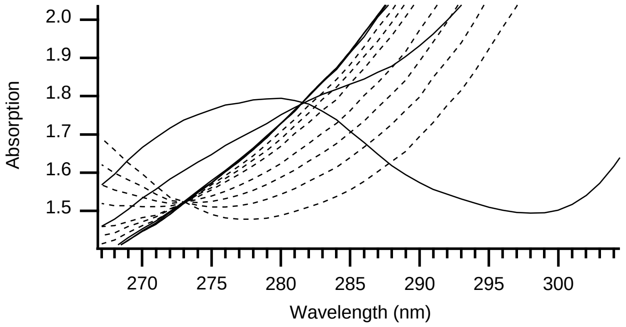

Supplementary Figure 135. UV-Vis spectra. Zoom of the UV-Vis spectra of $\mathbf{14}_{QC\text{-}NBD}$, sample 1, after varying irradiation times with a 365 nm diode.

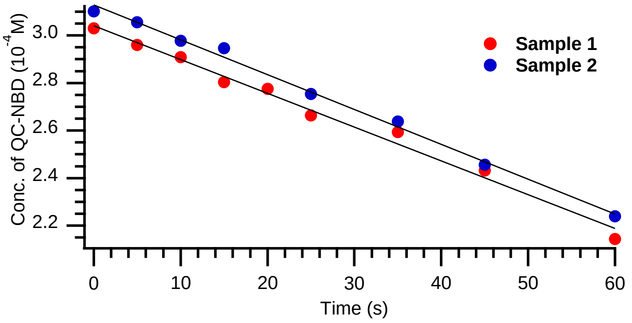

Supplementary Figure 136. Quantum yield plot. Plot of the quantum yield measurements for compound $\mathbf{14}_{QC\text{-}NBD}$ at 340 nm. Volume of sample 1: 3.114 mL. Volume of sample 2: 3.032 mL. $\Phi_{sample\ 1}$: 50%. $\Phi_{sample\ 2}$: 51%.

Supplementary Note 8. The extinction coefficient of QC-NBD was calculated based on the initial concentration of NBD-NBD, assuming that all NBD-NBD has been isomerized to QC-NBD. From the extinction coefficient the concentrations after varying irradiation times could be determined.

Compound 15

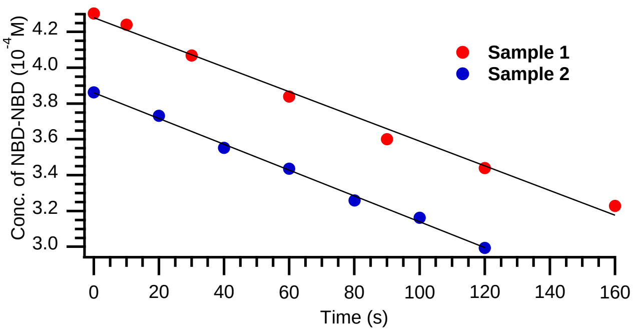

Supplementary Figure 137. Quantum yield plot. Plot of the quantum yield measurements for compound **15** at 310 nm. Volume of sample 1: 3.024 mL. Volume of sample 2: 3.045 mL. $\Phi_{sample\ 1}$: 51%. $\Phi_{sample\ 2}$: 54%.

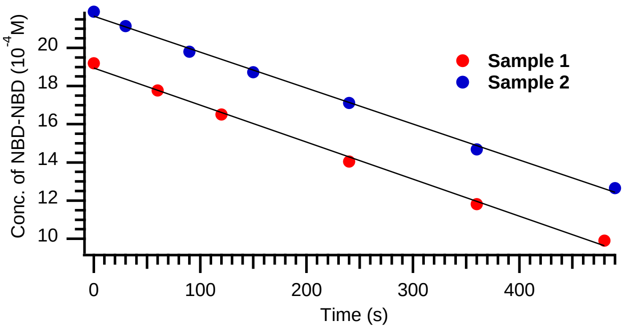

Supplementary Figure 138. Quantum yield plot. Plot of the quantum yield measurements for compound **15** at 340 nm. Volume of sample 1: 2.272 mL. Volume of sample 2: 2.981 mL. $\Phi_{sample\ 1}$: 49%. $\Phi_{sample\ 2}$: 54%.

Compound 5

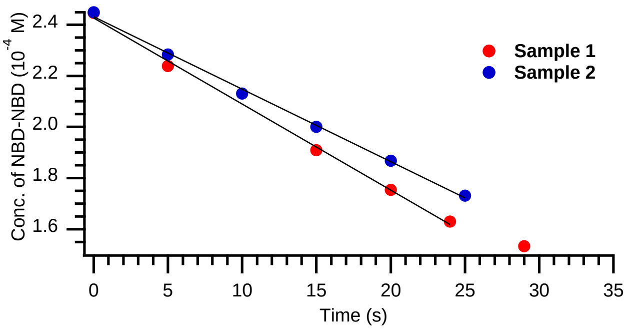

Supplementary Figure 139. Quantum yield plot. Plot of the quantum yield measurements for compound **5** at 365 nm. Volume of sample 1: 2.645 mL. Volume of sample 2: 2.947 mL. $\Phi_{sample\ 1}$: 47%. $\Phi_{sample\ 2}$: 47%.

Supplementary Note 9. It is assumed that the first process (**NBD-NBD** to **QC-NBD**) is dominating here. The measurement is done having an isosbestic point, and the measurement was stopped when deviation from the isosbestic point was seen, meaning that the process of **QC-NBD** to **QC-QC** is starting to compete. The concentration was determined at 381 nm, where the concentration of **QC-NBD** can be negligible.

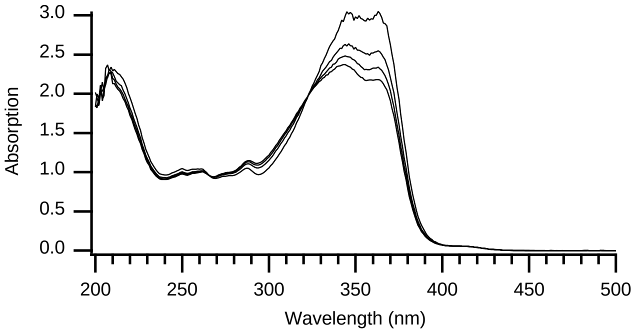

Supplementary Figure 140. UV-Vis spectra. UV-Vis spectra of **5**, sample 1, after varying irradiation times with a 365 nm diode.

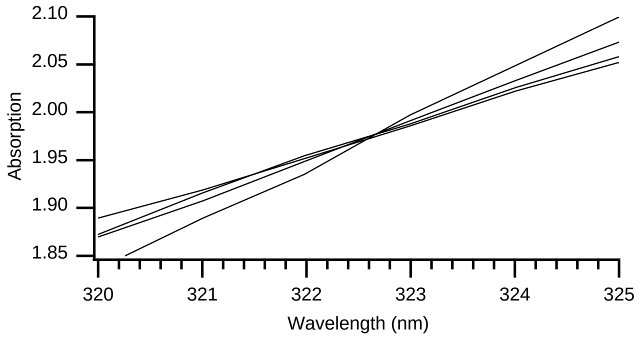

Supplementary Figure 141. UV-Vis spectra. Zoom of UV-Vis spectra of **5**, sample 1, after varying irradiation times with a 365 nm diode.

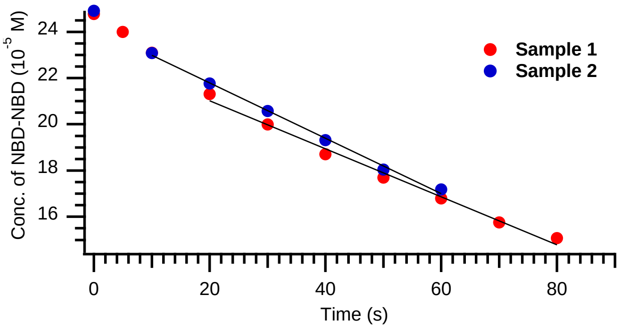

Supplementary Figure 142. Quantum yield plot. Plot of the quantum yield measurements for compound **5** at 340 nm. Volume of sample 1: 2.816 mL. Volume of sample 2: 2.891 mL. $\Phi_{sample\ 1}$: 33% $\Phi_{sample\ 2}$: 40%. Do not give goot fits due to two different quantum yields.

Supplementary Note 10. Here there are two competing quantum yields, the process of **NBD-NBD** to **QC-NBD** and **QC-NBD** to **QC-QC**, this is clear from the absence of an isosbestic point, which also explains the difference in the two measurements due to the bad fit of the data.

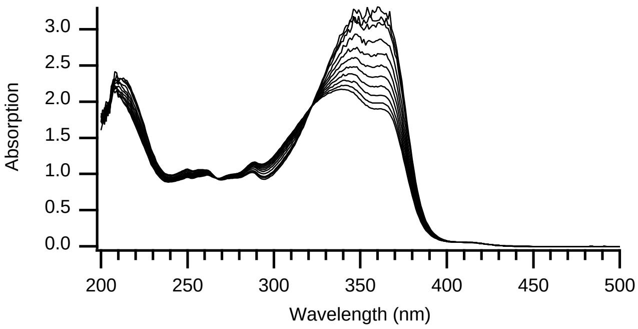

Supplementary Figure 143. UV-Vis spectra. UV-Vis spectra of **5**, sample 1, after varying irradiation times with a 340 nm diode.

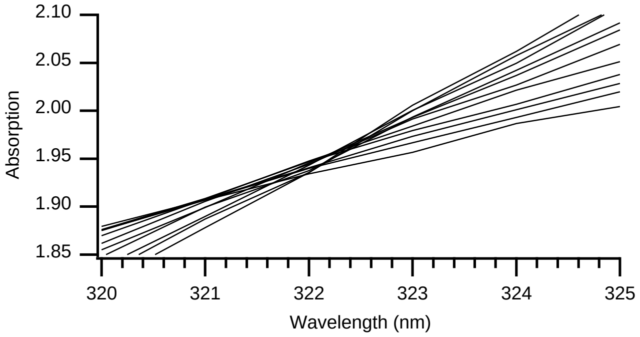

Supplementary Figure 144. UV-Vis spectra. Zoom of the UV-Vis spectra of **5**, sample 1, after varying irradiation times with a 340 nm diode.

Compound 8

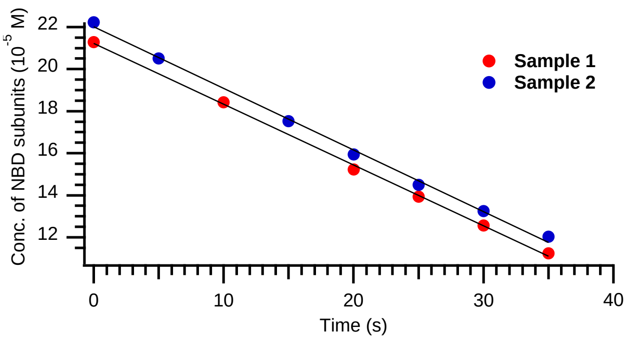

Supplementary Figure 145. Quantum yield plot. Plot of the quantum yield measurements for compound **8** at 340 nm. Volume of sample 1: 2.773 mL. Volume of sample 2: 2.882 mL. $\Phi_{sample\ 1}$: 92% $\Phi_{sample\ 2}$: 96%.

Supplementary Note 11. Example of quantum yield calculation of **8** sample 1:
The formal concentration of **8** was multiplied by 2, to get the concentration of NBD subunits.
Slope: -2.8932 x 10^{-6} M/s
Weight of the solution (cuvette and solution minus cuvette weight): 2.160 g
Density of cyclohexane: 0.779 g/mL
Volume in cuvette (determined from the weight): $V = \frac{2.160\ g}{0.779\ g/mL} = 2.773$ mL

$$\Phi = \frac{\Delta C}{\Delta t} * \frac{V}{I} = slope * \frac{V}{I} = -2.8932 \text{ x } 10^{-6}\ M/s * \frac{2.773 * 10^{-3}\ L}{8.76646 * 10^{-9}\ mol/s} = 0.92$$

Supplementary Note 12. When examining the spectra from this measurement it is evident that there is no isosbestic point, and that we have both photochemical reactions occuring (**NBD-NBD** to **QC-NBD** and **QC-NBD** to **QC-QC**). Thus, when the results from the quantum yield measurement are giving a straight line, it is assumed that the quantum yields for the two isomerisations are the same or very close.

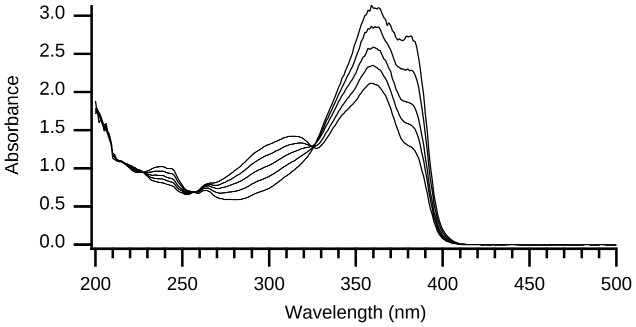

Supplementary Figure 146. UV-Vis spectra. UV-Vis spectra of **8**, sample 1, after varying irradiation times with a 340 nm diode.

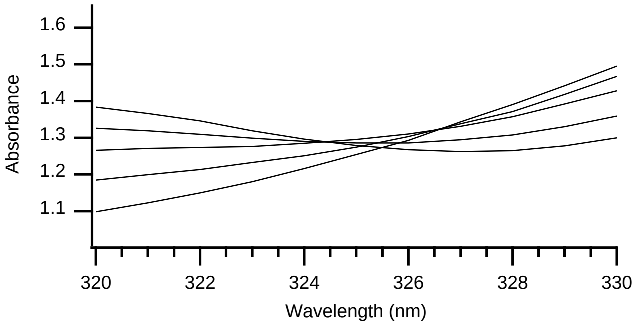

Supplementary Figure 147. UV-Vis spectra. Zoom of the UV-Vis spectra of **8**, sample 1, after varying irradiation times with a 340 nm diode.

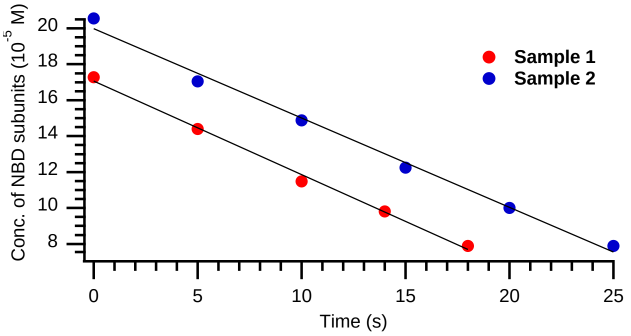

Supplementary Figure 148. Quantum yield plot. Plot of the quantum yield measurements for compound **8** at 365 nm. Volume of sample 1: 2.916 mL. Volume of sample 2: 3.007 mL. $\Phi_{sample\ 1}$: 85% $\Phi_{sample\ 2}$: 84%.

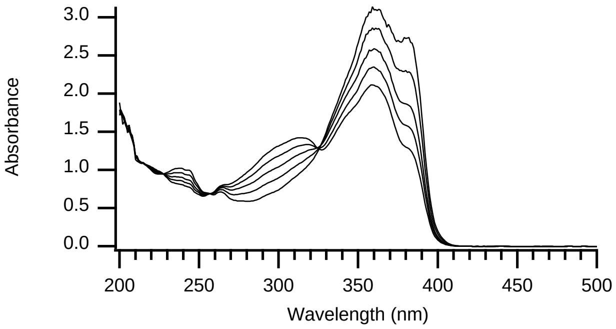

Supplementary Figure 149. UV-Vis spectra. UV-Vis spectra of **8**, sample 1, after varying irradiation times with a 365 nm diode.

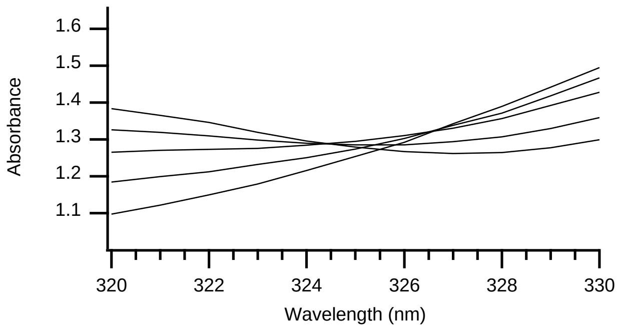

Supplementary Figure 150. UV-Vis spectra. UV-Vis spectra of **8**, sample 1, after varying irradiation times with a 365 nm diode.

Compound 9

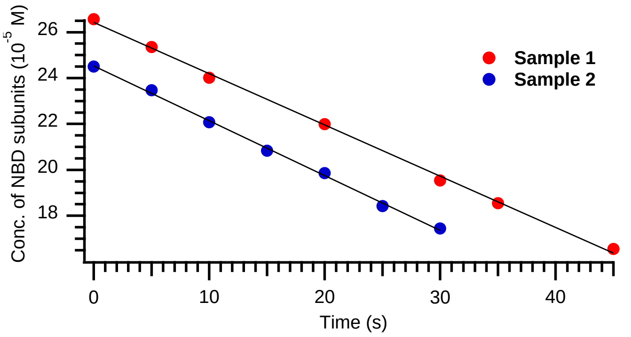

Supplementary Figure 151. Quantum yield plot. Plot of the quantum yield measurements for compound **9** at 340 nm. Volume of sample 1: 3.229 mL. Volume of sample 2: 3.083 mL. $\Phi_{sample\ 1}$: 82%. $\Phi_{sample\ 2}$: 84%.

Supplementary Note 13. When examining the spectra from this measurement it is evident that there is no isosbestic point, and that we have both photochemical reactions occuring (**NBD-NBD** to **QC-NBD** and **QC-NBD** to **QC-QC**). Thus, when the results from the quantum yield measurement are giving a straight line, it is assumed that the quantum yields for the two isomerisations are the same or very close.

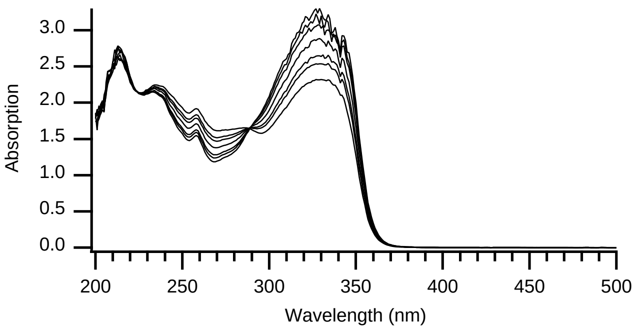

Supplementary Figure 152. UV-Vis spectra. UV-Vis spectra of **9**, sample 1, after varying irradiation times with a 340 nm diode.

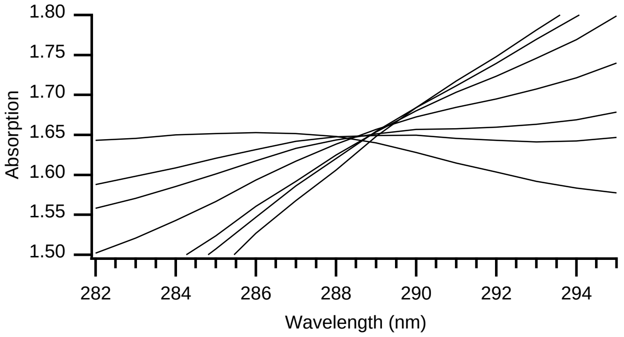

Supplementary Figure 153. UV-Vis spectra. Zoom of the UV-Vis spectra of **9**, sample 1, after varying irradiation times with a 340 nm diode.

Compound 11

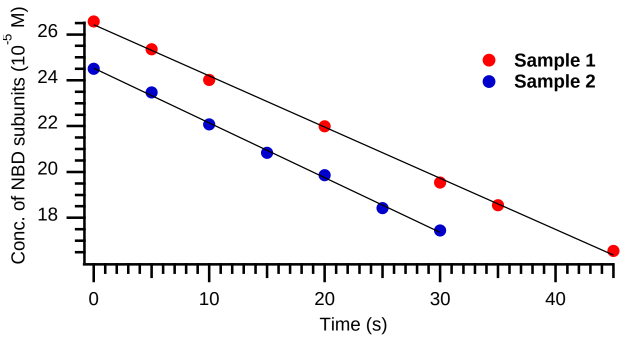

Supplementary Figure 154. Quantum yield plot. Plot of the quantum yield measurements for compound **11** at 340 nm. Volume of sample 1: 2.822 mL. Volume of sample 2: 3.052 mL. $\Phi_{\text{sample 1}}$: 72%. $\Phi_{\text{sample 2}}$: 69%.

Supplementary Note 14. When examining the spectra from this measurement it is evident that there is no isosbestic point, and that we have all the photochemical reactions occuring (**NBD-NBD-NBD** to **QC-NBD-NBD**, **QC-NBD-NBD** to **QC-QC-NBD** and **QC-QC-NBD** to **QC-QC-QC**). Thus, when the results from the quantum yield measurement are giving a straight line, it is assumed that the quantum yields for the three isomerisations are the same or very close.

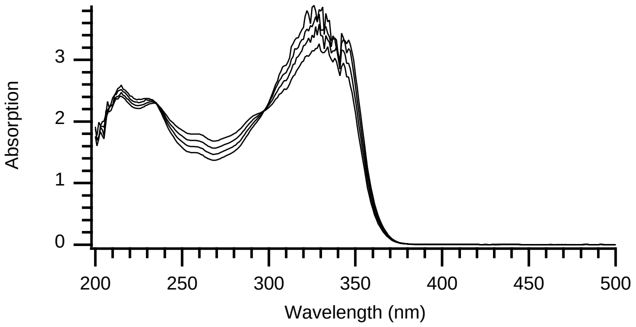

Supplementary Figure 155. UV-Vis spectra. UV-Vis spectra of **11**, sample 1, after varying irradiation times with a 340 nm diode.

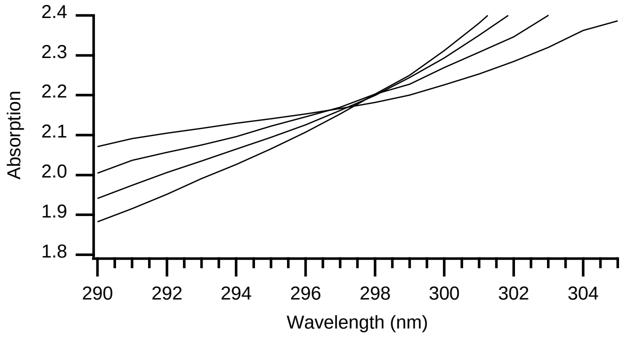

Supplementary Figure 156. UV-Vis spectra. Zoom of the UV-Vis spectra of **11**, sample 1, after varying irradiation times with a 340 nm diode.

Cyclability test

Compound 8

Supplementary Note 15. The measurement was done at 50 °C for 71 cycles in cyclohexane. One cycle consisted of irradiating for 600s at 365 nm after which the sample was kept in the dark for 5100s. Five points of the NBD-NBD form and five points of the QC-QC form were measured during the measurement to give the graph showed in the article. The figure below shows the spectrum before and after the measurement, the spectrum after 71 cycles is lower, which is presumed to be decomposition

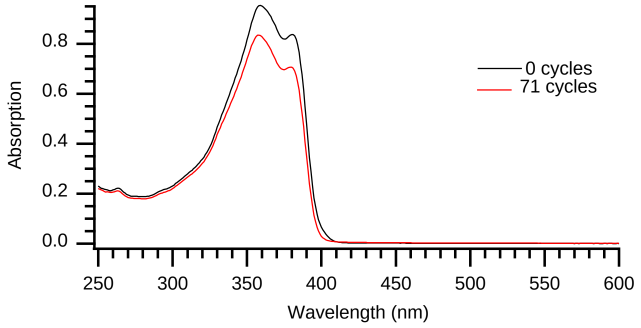

Supplementary Figure 157. UV-Vis spectra. UV-Vis spectra of **8**. Black line: before cyclability test. Red line: after 71 cycles.

Compound 14

Supplementary Note 16. The measurement was done at 70 °C for 13 cycles in cyclohexane. One cycle consisted of irradiating for 300s at 340 nm after which the sample was kept in the dark for 6h. Six points of the NBD-NBD form and six points of the QC-QC form were measured during the measurement to give the graph showed in the article. The figure below shows the spectrum before and after the measurement, which shows an increase in absorbance. This is not surprising, as a drop in the solvent level was observed during the experiment.

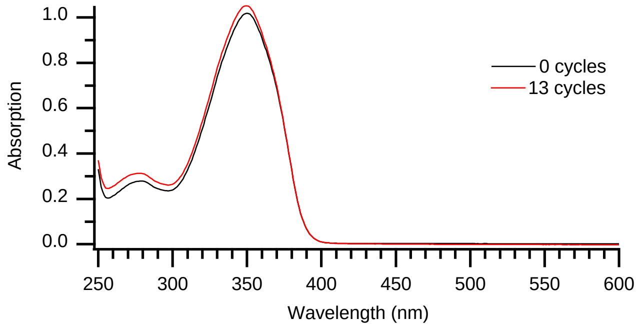

Supplementary Figure 158. UV-Vis spectra. UV-Vis spectra of **14**. Black line: before cyclability test. Red line: after 13 cycles.

Coordinates of all compounds by DFT calculations

Compound	x, y, z coordinates
5: NBD-NBD	C -0.537628 -4.394438 -1.239450<br>C -0.127824 4.313166 0.129223<br>C -0.807178 2.932367 0.283471<br>N -3.334092 2.594201 0.576725<br>C -2.191570 2.728690 0.441250<br>H 2.395799 2.184607 0.131442<br>C -0.133415 -2.001873 0.202152<br>H 1.937669 3.175855 -2.293747<br>C -1.486089 -2.757030 0.097772<br>C -1.488644 -3.459794 -1.270869<br>C -1.138534 -3.976641 1.000104<br>C 0.832006 -2.960312 0.238812<br>H -1.902620 -4.755265 0.973344<br>H -2.359325 -2.157179 0.343876<br>C 0.118483 -4.330132 0.152388<br>H 0.722687 -5.184175 0.450871<br>H -0.905708 -3.704310 2.032019<br>N 3.380508 -2.671194 0.322113<br>C 2.228218 -2.782223 0.281324<br>C 0.054115 0.600667 0.191008<br>C -0.001564 -0.613989 0.198195<br>C 0.410902 4.377108 -1.312199<br>C 1.494504 2.766907 -0.046117<br>C 1.200671 3.988180 0.873237<br>H -0.720515 5.159281 0.470358<br>H 1.946025 4.779522 0.778264<br>H 1.058078 3.720816 1.922711<br>C 1.372480 3.458560 -1.415149<br>H 0.008146 5.016230 -2.087332<br>C 0.168657 1.989971 0.174235<br>H -0.210710 -5.045676 -2.039765<br>H -2.118925 -3.172830 -2.102586
5: QC-QC	C -0.053927 -3.909049 -1.150511<br>C 0.073895 4.362268 0.570521<br>C -0.658249 3.167719 -0.045588<br>N -3.212501 2.906528 -0.226266<br>C -2.065705 3.025861 -0.140531<br>H -1.535065 -2.436127 -2.240516<br>C -0.380157 -1.943514 -0.249719<br>H 0.400504 -4.498140 -1.934681<br>C -1.710030 -2.633759 0.055782<br>C -1.094778 -2.782990 -1.316243<br>C -1.479984 -3.881666 0.885907<br>C 0.673534 -3.090335 -0.079480<br>H -2.263173 -4.630654 0.729932<br>H -2.582809 -2.005202 0.181552

	C -0.155008 -4.321335 0.295361<br>H 0.373782 -5.202438 0.638279<br>H -1.409001 -3.665777 1.955647<br>N 3.201160 -2.804565 0.313622<br>C 2.065517 -2.936253 0.141082<br>C 0.131940 0.627542 -0.120196<br>C -0.118011 -0.553533 -0.181284<br>C 0.286632 4.033608 -0.884726<br>C 1.638109 2.666415 0.573872<br>C 1.238226 3.868886 1.406212<br>H -0.513435 5.230896 0.842529<br>H 2.039036 4.612986 1.466999<br>H 0.936895 3.593785 2.420819<br>C 1.334738 2.901987 -0.887251<br>H 1.963603 2.600630 -1.713137<br>C 0.403329 2.015550 -0.050963<br>H 0.014683 4.672758 -1.713020<br>H 2.460682 2.017853 0.848349
8: NBD-NBD	C -0.229388 6.683009 -0.686482<br>C -1.433041 7.227479 0.103457<br>H -1.794328 -7.525271 1.795653<br>H 0.199202 -8.397039 0.174335<br>N 1.908653 4.937154 3.324770<br>C 0.573025 7.605282 1.198839<br>C -0.432006 -7.521748 0.036212<br>H 1.737294 -1.245794 0.508857<br>C -0.957690 7.775118 1.222798<br>H -1.724849 -0.872118 -2.014010<br>C -0.502884 -5.199941 -0.261047<br>C 0.363682 5.520101 0.151824<br>H 1.833133 7.534383 -0.637369<br>H 0.533110 8.770082 -0.694386<br>C 0.852734 6.086545 1.287498<br>C -2.489592 -6.659773 -0.582889<br>H -0.396518 6.448245 -1.735579<br>H 1.145560 8.220743 1.889605<br>C -0.812865 0.966670 -1.392440<br>C -1.081098 -6.869868 1.292409<br>H -2.465880 7.107891 -0.197302<br>C 0.196007 1.576028 -0.622148<br>H 1.915076 1.214816 0.629819<br>C 0.299546 -6.266816 -0.502955<br>C 1.126734 0.754904 0.045202<br>H -0.351515 -6.479715 2.005822<br>C -1.771041 -5.764852 0.439462<br>C 1.024995 -0.623322 -0.020167<br>H -2.374542 -5.016516 0.948677<br>H -1.845866 -8.512072 -1.527486

	C -0.925896 -0.413969 -1.441995<br>C -0.022689 -1.232279 -0.738117<br>C -1.695777 -7.704623 -0.822543<br>C 0.814918 7.781276 -0.328402<br>N 2.571461 -6.355539 -1.693985<br>C 1.545109 -6.291702 -1.161464<br>C 1.428419 5.438858 2.397658<br>C -0.306601 -3.840871 -0.536157<br>C -0.184962 -2.642724 -0.681683<br>C 0.256058 2.982543 -0.452873<br>C 0.314018 4.166086 -0.190621<br>H -1.513480 8.204231 2.046549<br>H -3.436936 -6.418175 -1.047308<br>H -1.523672 1.587707 -1.925897
8: QC-QC	C 1.360939 6.211017 -1.010121<br>C -0.091196 6.518440 -1.288229<br>H -1.811239 -8.412147 0.830711<br>H 0.876527 -8.437905 0.594827<br>N -1.348422 5.909808 3.092877<br>C 0.750222 7.682888 0.655981<br>C 0.161966 -7.691436 0.270140<br>H 1.714600 -1.388959 -0.176582<br>C -0.496662 7.501697 -0.171220<br>H -2.566242 -1.022560 -0.304288<br>C -0.570879 -5.423762 -0.292773<br>C 0.273614 5.453989 -0.244782<br>H 2.776689 6.889839 0.504135<br>H 2.377115 8.117103 -0.719949<br>C -0.136030 6.457194 0.889067<br>C -1.159049 -6.420469 -1.304971<br>H 1.935552 5.654940 -1.740366<br>H 0.774304 8.443095 1.427295<br>C -1.466493 0.818282 -0.273250<br>C -1.191432 -7.515069 0.930239<br>H -1.705500 -6.188340 -2.208158<br>C -0.201638 1.432846 -0.235447<br>H 1.921791 1.071907 -0.161759<br>C 0.700668 -6.329469 -0.171458<br>C 0.940862 0.611518 -0.197409<br>H -1.109186 -7.260544 1.990501<br>C -1.715315 -6.362179 0.096283<br>C 0.825882 -0.768720 -0.205416<br>H -2.688628 -5.918881 0.265856<br>H 0.618395 -7.810742 -1.987153<br>C -1.584209 -0.563525 -0.277331<br>C -0.440516 -1.382670 -0.247257<br>C 0.097807 -7.305995 -1.185480<br>C 1.979862 7.281820 -0.134182

	N 3.138254 -5.538441 0.071415<br>C 2.043286 -5.893833 -0.034247<br>C -0.800572 6.151482 2.103945<br>C -0.584754 -4.010245 -0.274695<br>C -0.541330 -2.800904 -0.259161<br>C -0.067098 2.850337 -0.238717<br>C 0.084855 4.050939 -0.248935<br>H -1.350528 8.164499 -0.163559<br>H -2.356178 1.437568 -0.297452<br>H -0.594289 6.367027 -2.232921
8: NBD-QC	C 1.557782 6.221095 -0.185279<br>C 1.192456 7.024991 -1.445344<br>H -1.779359 -8.395727 0.783798<br>H 0.850969 -8.571911 0.203976<br>N -3.075235 5.955317 1.296580<br>C 0.004328 7.728293 0.413834<br>C 0.151410 -7.769811 0.002909<br>H 1.864228 -1.380238 -0.692587<br>C 0.271065 7.921709 -1.090034<br>H -2.289312 -1.083365 0.379240<br>C -0.495867 -5.437999 -0.371193<br>C 0.292392 5.411701 0.208017<br>H 1.493278 7.000640 1.903813<br>H 2.165241 8.160947 0.710972<br>C -0.641705 6.330624 0.574083<br>C -1.271039 -6.349762 -1.337971<br>H 2.474865 5.637084 -0.225276<br>H -0.511146 8.536221 0.928948<br>C -1.222584 0.769164 0.240582<br>C -1.093730 -7.543929 0.837910<br>H -1.909481 -6.041394 -2.153765<br>C 0.008519 1.402185 -0.023756<br>H 2.071392 1.076937 -0.567139<br>C 0.718638 -6.422764 -0.450821<br>C 1.118262 0.602619 -0.361888<br>H -0.862179 -7.345312 1.887942<br>C -1.642010 -6.324027 0.123791<br>C 1.002916 -0.775441 -0.432855<br>H -2.555984 -5.832571 0.433136<br>H 0.311459 -7.809002 -2.297570<br>C -1.337081 -0.608958 0.170576<br>C -0.227506 -1.407877 -0.167238<br>C -0.069612 -7.312871 -1.416108<br>C 1.444481 7.357216 0.872444<br>N 3.212529 -5.786993 -0.496017<br>C 2.093142 -6.074470 -0.471532<br>C -1.975283 6.104699 0.964388<br>C -0.428302 -4.028331 -0.296281

	C -0.340837 -2.822475 -0.236983<br>C 0.121033 2.812094 0.052425<br>C 0.186843 4.022108 0.127945<br>H 1.583069 6.822378 -2.434193<br>H -2.082998 1.374061 0.503140<br>H -0.263068 8.619069 -1.722547
9: NBD-NBD	C 0.480125 6.115607 -0.719809<br>C -0.599866 6.390040 -1.781792<br>H -3.587217 -6.061148 -0.277102<br>H -1.594885 -7.595330 0.736574<br>N -2.657473 3.850416 2.238666<br>C -1.384830 6.729676 0.370430<br>C -1.496728 -6.697335 0.130353<br>H 3.489516 -2.241643 -0.194980<br>C -1.707064 6.757063 -1.134956<br>H -0.057160 0.175808 0.052363<br>C -0.594933 -4.541997 -0.034986<br>C 0.007471 4.868837 0.075993<br>H 0.618423 7.121718 1.266549<br>H 0.195985 8.224708 -0.084293<br>C -1.116358 5.249540 0.737398<br>C -1.610920 -5.717925 -1.965239<br>H 1.509370 6.068107 -1.069114<br>H -2.075201 7.250802 1.030267<br>C 1.798428 1.259260 -0.103993<br>C -2.660910 -5.664047 0.141747<br>H -0.464873 6.234638 -2.844383<br>C 3.196079 1.144772 -0.222339<br>H 4.873094 -0.190169 -0.330714<br>C -0.353411 -5.742604 0.552235<br>C 3.794246 -0.110138 -0.250130<br>H -2.843196 -5.224095 1.124672<br>C -1.913226 -4.713895 -0.837649<br>C 3.022211 -1.263984 -0.174862<br>H -2.393124 -3.781815 -1.127987<br>H 3.800243 2.042803 -0.281211<br>C 1.019225 0.094147 -0.036847<br>C 1.620555 -1.172098 -0.079437<br>C -1.364672 -6.896249 -1.390865<br>C 0.093523 7.213268 0.312983<br>N 1.633437 -6.454687 2.012634<br>C 0.747997 -6.115516 1.347629<br>C -1.951502 4.463700 1.555381<br>C 0.176367 -3.374692 -0.019068<br>C 0.827062 -2.351745 -0.040338<br>C 1.176281 2.536478 -0.046229<br>C 0.624067 3.614185 0.023101<br>H -2.684219 6.971905 -1.548083

	H -1.553624 -5.459626 -3.014760<br>H -1.059071 -7.821001 -1.863257
9: QC-QC	C 0.644798 6.221714 -0.691247<br>C -0.600683 5.853502 -1.460364<br>H -3.252260 -6.799352 -0.821559<br>H -1.418682 -7.817560 0.878914<br>N -2.802705 4.333489 2.279542<br>C -1.086097 7.087141 0.561037<br>C -1.349292 -6.961832 0.218312<br>H 3.219378 -2.192863 -0.005895<br>C -1.756615 6.432042 -0.618956<br>H -0.387548 0.121144 -0.334771<br>C -0.518167 -4.766342 -0.484823<br>C -0.152870 4.982218 -0.277954<br>H 0.999137 7.330377 1.154250<br>H 0.506409 8.345118 -0.220973<br>C -1.326489 5.575138 0.574963<br>C -0.618951 -5.695721 -1.704695<br>H 1.615793 6.060805 -1.142992<br>H -1.670631 7.698807 1.237573<br>C 1.434097 1.258793 -0.151050<br>C -2.566552 -6.179454 -0.234720<br>H -2.768302 6.625918 -0.946498<br>C 2.832399 1.181794 -0.012173<br>H -0.634344 5.576523 -2.504674<br>C -0.165891 -6.009445 0.403285<br>C 3.466316 -0.054140 0.039087<br>H -3.116904 -5.742724 0.603145<br>C -1.875141 -5.131441 -1.085235<br>C 2.727289 -1.227993 -0.047543<br>H -2.420948 -4.352832 -1.603415<br>H 3.407530 2.098102 0.057712<br>C 0.690222 0.071605 -0.234286<br>C 1.327590 -1.177624 -0.186116<br>C -0.266591 -6.913706 -0.828597<br>C 0.372027 7.370253 0.259133<br>N 1.533074 -6.112687 2.334318<br>C 0.765487 -6.060973 1.471384<br>C -2.134552 4.888596 1.516886<br>H 0.393551 -7.730369 -1.084819<br>C 0.595448 -2.398178 -0.282867<br>C 0.807562 2.539649 -0.204712<br>C 0.352929 3.660094 -0.241395<br>H -0.267737 -5.494900 -2.706965<br>C 0.055079 -3.476185 -0.380298<br>H 4.544612 -0.103466 0.149289
11: NBD-NBD-NBD	C 0.534077 6.411902 -0.319988<br>C 0.268160 7.008285 -1.713847

	H -7.673836 -3.637257 0.827799<br>C 7.011617 -3.597551 -1.118490<br>N -4.156575 5.297140 0.366623<br>C -1.363816 7.606987 -0.183260<br>C -5.993997 -4.901064 0.104171<br>H -2.251422 1.029801 0.140837<br>C -0.857362 7.719180 -1.633136<br>H 2.029974 1.435621 0.151582<br>C -4.404887 -3.182794 0.185743<br>C -0.606298 5.395079 -0.048447<br>H -0.072609 7.341959 1.615223<br>H 0.578795 8.506977 0.416065<br>C -1.749830 6.124627 0.037268<br>C -6.331457 -3.148827 -1.372485<br>H 1.536131 6.032167 -0.132635<br>H -2.112155 8.330546 0.133029<br>C -0.127455 1.397812 0.143117<br>C -6.587095 -3.703438 0.902148<br>H 0.874205 6.809535 -2.588138<br>C 1.146403 0.809671 0.173296<br>H -6.661754 -5.168929 -2.111904<br>C -4.496495 -4.537411 0.248156<br>C 1.286032 -0.586533 0.230342<br>H -6.272699 -3.679224 1.947970<br>C -5.855414 -2.660542 0.007431<br>C 0.138839 -1.394703 0.253889<br>H -5.987871 -1.603108 0.225874<br>H 5.297035 -4.788648 -1.734797<br>C -1.267413 0.578109 0.166638<br>C -1.141108 -0.818560 0.217479<br>C -6.415461 -4.478658 -1.315432<br>C 0.007363 7.587540 0.553858<br>N -2.608530 -6.265631 0.435789<br>C -3.447742 -5.472012 0.348683<br>C -3.064669 5.653341 0.216511<br>H -6.493435 -2.503000 -2.225738<br>H 7.689943 -3.617618 -1.961724<br>C 4.963139 -2.236221 0.270953<br>H -6.255239 -5.910706 0.414009<br>H 0.239671 -2.472252 0.294626<br>C 5.230097 -3.762743 0.353542<br>C 5.817990 -4.181777 -1.005626<br>C 6.544370 -3.721269 1.186718<br>C 6.182252 -1.645386 0.160600<br>H 7.028207 -4.696432 1.263601<br>H 4.394668 -4.350005 0.728506<br>C 7.239535 -2.775715 0.163856<br>H 8.258755 -2.452957 0.365516

	H 6.421086 -3.275821 2.176480<br>N 6.737750 0.843189 -0.131807<br>C 6.468793 -0.275993 -0.002047<br>C -0.438505 4.009539 0.023693<br>C -0.273190 2.809808 0.081217<br>C -2.296049 -1.645642 0.219889<br>C -3.266638 -2.372128 0.207870<br>C 2.579545 -1.173286 0.253601<br>C 3.693231 -1.652499 0.260515<br>H -1.383107 8.233964 -2.426942
11: QC-QC-QC	C -0.721005 6.525707 -0.979617<br>C -2.153619 6.332692 -1.417502<br>H -6.697586 -5.683772 0.272286<br>C 6.892785 -2.042185 -0.578782<br>N -3.557611 5.059743 2.770787<br>C -1.971405 7.592886 0.637217<br>C -4.596346 -6.216658 0.430906<br>H 0.380807 -2.293881 -0.218905<br>C -2.987002 7.045528 -0.332652<br>H -2.656011 0.734892 -0.495058<br>C -3.753712 -4.070893 -0.394767<br>C -1.548443 5.396831 -0.362992<br>H 0.207892 7.552355 0.706694<br>H -0.463308 8.642147 -0.527647<br>C -2.393720 6.125722 0.737569<br>C -4.479850 -4.809410 -1.530326<br>H 0.082420 6.245527 -1.649067<br>H -2.291483 8.265549 1.424147<br>C -0.625331 1.453016 -0.399294<br>C -5.744255 -5.235461 0.570546<br>H -4.013767 7.373437 -0.409558<br>C 0.726316 1.087875 -0.306185<br>H -2.473386 6.075722 -2.417558<br>C -3.294560 -5.453581 0.181991<br>C 1.098247 -0.262378 -0.238356<br>H -5.840413 -4.846536 1.588082<br>C -5.278009 -4.179922 -0.413771<br>C 0.103336 -1.248637 -0.275290<br>H -5.844754 -3.277674 -0.607514<br>H 1.487919 1.857479 -0.273776<br>C -1.611594 0.454746 -0.432611<br>C -1.252020 -0.899157 -0.371763<br>C -4.020949 -6.167443 -0.961374<br>C -0.605032 7.686927 -0.012118<br>N -1.010509 -5.969717 1.251283<br>C -2.039369 -5.744346 0.774435<br>C -3.028529 5.534892 1.859416<br>H -3.667621 -7.022711 -1.520421

	H 5.158577 -3.636304 -0.566471<br>C 4.949049 -1.424608 0.207864<br>H 7.502357 -2.285171 -1.437589<br>H -4.523777 -4.524062 -2.571917<br>C 5.446185 -2.346250 1.327267<br>C 5.615820 -2.769792 -0.109861<br>C 6.752467 -1.828286 1.894390<br>C 6.247742 -0.689551 -0.262336<br>H 7.366472 -2.631385 2.315006<br>H 4.697291 -2.830902 1.941114<br>C 7.356538 -1.261986 0.624364<br>H 8.323094 -0.773655 0.605020<br>H 6.600665 -1.063728 2.661443<br>N 6.400084 1.523189 -1.568266<br>C 6.327998 0.530761 -0.979938<br>C -1.243086 4.015605 -0.413754<br>C -0.982887 2.834562 -0.424822<br>C -2.222853 -1.942474 -0.394130<br>C -2.966735 -2.895870 -0.410790<br>C 2.465365 -0.642230 -0.108887<br>C 3.612788 -0.999046 0.026273<br>H -4.543836 -7.137114 0.999449
14: NBD-NBD	C -1.502545 -3.655027 0.402772<br>C -1.806237 -4.385564 -0.919265<br>H -0.743363 6.064862 0.300979<br>C -0.965969 -4.859111 1.228536<br>H 2.327853 3.093923 0.808410<br>C 0.074601 -5.221636 0.134547<br>C 0.852471 5.318283 -1.097595<br>H -2.606577 -4.118665 -1.597330<br>C -0.867133 -5.319162 -1.078358<br>H 0.698142 5.985472 -1.935827<br>H 2.588173 4.118014 -1.630501<br>C -0.172362 -2.880221 0.205016<br>C 0.972481 4.864570 1.208940<br>H -2.320299 -3.083919 0.835356<br>C 0.776820 -3.841809 0.045012<br>C 1.235413 0.627030 0.188009<br>H -1.717776 -5.635431 1.382534<br>H -0.514113 -4.569698 2.180285<br>C -0.071127 -1.425744 0.173218<br>C 1.794626 4.386966 -0.945130<br>H -0.722856 -5.990085 -1.915539<br>C -1.230706 -0.625962 0.138332<br>C 0.075394 1.427610 0.167324<br>H -2.209022 -1.089425 0.107791<br>C 1.167699 -0.755997 0.191634<br>H 0.740216 -6.064737 0.308715

	H -2.087705 1.318285 0.115761<br>C -1.162986 0.756904 0.134897<br>C -0.077522 5.222798 0.123419<br>N 3.289640 -3.677306 -0.448693<br>C -0.778120 3.841768 0.042040<br>C 1.504497 3.660674 0.381491<br>N -3.293315 3.678949 -0.441315<br>H 2.092160 -1.317654 0.220137<br>C 0.173935 2.881878 0.196321<br>H 2.214460 1.089342 0.202411<br>H 1.723899 5.642605 1.355604<br>H 0.529134 4.575902 2.164735<br>C 2.151857 -3.722826 -0.232774<br>C -2.155006 3.723981 -0.227355
14: QC-QC	C -1.477461 -3.619490 0.686171<br>C -1.264234 -3.746638 -0.814548<br>H -0.941234 6.012624 0.672117<br>C -0.976252 -4.858706 1.402128<br>H 2.381824 3.183123 0.996897<br>C 0.154054 -5.232712 0.463910<br>C 0.070778 4.820279 -0.943468<br>H -0.238303 5.376667 -1.817704<br>C -0.169721 -4.825469 -0.949574<br>H 1.956562 3.572407 -1.605597<br>H -1.985671 -3.456753 -1.566564<br>C -0.343779 -2.867333 0.013598<br>C 0.928711 4.903466 1.388708<br>H -2.326626 -3.050200 1.045247<br>C 0.780975 -3.960051 -0.114969<br>C 0.196065 0.682996 -1.192029<br>H -1.736028 -5.645017 1.455725<br>H -0.621592 -4.646437 2.415268<br>C -0.139131 -1.398187 0.010058<br>C 1.233565 3.811497 -0.837301<br>C -2.179517 3.587329 -0.224396<br>C -0.072074 -0.682119 1.211626<br>C 0.252158 1.399769 0.007819<br>H -0.177994 -1.209682 2.154675<br>C 0.005430 -0.695512 -1.190921<br>H 0.082087 -5.398900 -1.831157<br>H 0.162364 1.233143 2.153070<br>C 0.120144 0.695316 1.210777<br>C -0.244611 5.205684 0.478674<br>N 3.303477 -3.559580 -0.436049<br>C -0.802750 3.897072 -0.087732<br>C 1.490224 3.697940 0.658757<br>N -3.300345 3.327140 -0.341631<br>H -0.040232 -1.232823 -2.132987

	C 0.391019 2.876432 0.009040<br>H 0.297338 1.211525 -2.134621<br>H 1.638598 5.735925 1.425802<br>H 0.612567 4.668755 2.409625<br>C 2.171030 -3.741171 -0.286423<br>H 0.801046 -6.083454 0.640053
14: NBD-QC	C 1.548752 3.561499 0.032371<br>C 1.589570 4.342625 -1.294696<br>H -2.191812 -5.769394 0.050492<br>H 1.795527 -1.374023 -0.998318<br>N -3.314939 3.689533 0.152161<br>C -0.008395 5.166547 0.160639<br>C -0.101004 -5.274012 0.379835<br>H 2.222448 4.090574 -2.136192<br>C 0.662063 5.297993 -1.218347<br>H -1.954227 1.281078 0.836634<br>C -0.323784 -2.921118 -0.291356<br>C 0.186067 2.823621 0.090005<br>H 0.978755 4.418001 2.010889<br>H 2.016418 5.494602 1.018781<br>C -0.750370 3.804574 0.168169<br>C -0.530384 -3.826273 -1.506442<br>H 2.419078 2.951220 0.263307<br>H -0.599224 6.014449 0.501474<br>C 0.041755 1.373578 0.007621<br>C -1.580703 -4.955415 0.453226<br>H 0.363158 6.004299 -1.982263<br>C 1.091411 0.581406 -0.494036<br>H 1.219448 -5.410366 -1.501760<br>C 0.698812 -3.970804 0.276517<br>C 0.968375 -0.797202 -0.597714<br>H -1.909325 -4.742453 1.474626<br>C -1.600785 -3.725427 -0.434257<br>C -0.203474 -1.449123 -0.190116<br>H -2.520870 -3.203811 -0.668880<br>H 2.011620 1.048680 -0.825210<br>C -1.130113 0.714260 0.421622<br>C -1.243587 -0.665967 0.328066<br>C 0.469672 -4.859417 -0.950870<br>C 1.228431 4.739318 0.997118<br>N 2.900540 -3.487104 1.519354<br>C 1.907383 -3.706187 0.968768<br>C -2.156224 3.712884 0.154137<br>H 0.346376 -6.099976 0.919487<br>H -0.660108 -3.538865 -2.540937<br>H -2.152992 -1.145414 0.674335
15: NBD-NBD	C 1.112546 3.923949 0.310253<br>C 0.900813 4.676303 -1.017033

	H -2.785594 -2.182795 -1.493571<br>H 4.057746 -0.161095 -0.121877<br>N -3.103381 1.538465 -0.157813<br>C -1.050686 4.490956 0.207433<br>C -1.013798 -4.515930 0.187863<br>H 1.666912 4.824193 -1.767474<br>C -0.387711 5.014934 -1.076845<br>H -0.849164 0.153238 0.292735<br>C -0.018121 -2.382694 0.230731<br>C 0.334778 2.586354 0.201568<br>H -0.049214 4.266637 2.183409<br>H 0.394469 5.769952 1.309926<br>C -0.976130 2.943238 0.143761<br>C -2.219176 -2.829258 -0.836032<br>H 2.138756 3.847117 0.660404<br>H -2.030416 4.894104 0.455414<br>C 0.974702 1.272198 0.136687<br>C -1.690992 -3.669707 1.299060<br>H -0.916272 5.503799 -1.884989<br>C 2.370354 1.160447 0.016702<br>H -2.144926 -4.762915 -1.834036<br>C 0.295780 -3.694541 0.064058<br>C 2.978937 -0.091509 -0.030168<br>H 2.725498 -2.210326 0.012669<br>C -1.551459 -2.360952 0.470784<br>C 2.223329 -1.252412 0.042155<br>H -1.955398 -1.451889 0.907830<br>H 2.988354 2.047540 -0.046296<br>C 0.224025 0.084230 0.201906<br>C 0.818457 -1.183602 0.155751<br>C -1.900847 -4.113368 -1.003123<br>C 0.118295 4.719269 1.203374<br>N 2.515701 -4.854806 -0.497899<br>C 1.525740 -4.305313 -0.249933<br>C -2.127223 2.150059 -0.026443<br>H -0.872200 -5.581382 0.358602<br>H -2.731539 -3.946348 1.478007<br>H -1.131337 -3.655589 2.237519
15: QC-QC	C 1.294061 3.962463 -0.194146<br>C 0.580264 3.431323 -1.423590<br>H -0.735585 0.329479 -0.547243<br>H 2.757456 2.035304 1.273607<br>N -3.024387 1.947144 0.994090<br>C -0.863936 4.747848 -0.002138<br>C -0.911313 -4.612111 0.016377<br>H -1.208126 -4.231420 2.143514<br>C -0.861930 3.956133 -1.283303<br>H 2.156468 -2.207049 1.354029

	C -0.260203 -2.243967 0.104444<br>C 0.476944 2.689359 -0.088720<br>H 0.669628 5.235429 1.468057<br>H 0.840595 6.094482 -0.079449<br>C -0.994182 3.221897 0.056070<br>C -1.539924 -2.387405 -0.703060<br>H 2.374241 3.895236 -0.152622<br>H 1.068405 3.106650 -2.332292<br>C 0.953001 1.345301 0.315629<br>C -1.679221 -4.017474 1.179679<br>H -1.172833 -4.151956 -2.225865<br>C 2.156335 1.172436 1.008161<br>H -2.086097 -1.639837 -1.262143<br>C 0.207494 -3.650369 -0.401152<br>C 2.585999 -0.104454 1.364066<br>H 3.525955 -0.228527 1.891953<br>C -1.589108 -2.552910 0.800105<br>C 1.813190 -1.220385 1.061969<br>H -2.032604 -1.768206 1.400914<br>H -1.612165 4.031647 -2.058355<br>C 0.191767 0.211342 0.002902<br>C 0.595783 -1.071035 0.382734<br>C -1.083896 -3.768383 -1.219012<br>C 0.545372 5.148141 0.384851<br>N 2.582588 -4.390094 -1.065509<br>C 1.516628 -4.055361 -0.766073<br>C -2.112852 2.527616 0.581219<br>H -0.729047 -5.675296 -0.082761<br>H -2.713407 -4.375834 1.209149<br>H -1.732748 5.340041 0.258841

References

1. Stranius, K., Börjesson, K. Determining the Photoisomerization Quantum Yield of Photoswitchable Molecules in Solution and in the Solid State. *Sci. Rep.* 7, 41145 (2017).
2. Quant, M., Lennartsson, A., Dreos, A., Kuisma, M., Erhart, P., Börjesson, K. & Moth-Poulsen, K. Low Molecular Weight Norbornadiene Derivatives for Molecular Solar-Thermal Energy Storage. *Chem. Eur. J.* **22**, 13265–13274 (2016).
3. Sharma, K., Ram, S. & Chandaka, N. Transition Metal-Free Approach to Propynenitriles and 3-Chloropropenenitriles. *Adv. Synth. Catal.* **358**, 894–899 (2016).
4. Kuisma, M. J., Lundin, A. M., Moth-Poulsen, K., Hyldgaard, P. & Erhart, P. Comparative Ab-Initio Study of Substituted Norbornadiene-Quadricyclane Compounds for Solar Thermal Storage. *J. Phys. Chem. C* **120**, 3635–3645 (2016).
5. Valiev, M., Bylaska, E. J., Govind, N., Kowalski, K., Straatsma, T. P., van Dam, H. J. J., Wang, D., Nieplocha, J., Apra, E., Windus, T. L. & de Jong, W. A. NWChem: A comprehensive and scalable open-source solution for large scale molecular simulations. *Comput. Phys. Commun.* **181**, 1477-1489 (2010).
